# Single‐cell profiling and zebrafish avatars reveal *LGALS1* as immunomodulating target in glioblastoma

**DOI:** 10.15252/emmm.202318144

**Published:** 2023-10-04

**Authors:** Lise Finotto, Basiel Cole, Wolfgang Giese, Elisabeth Baumann, Annelies Claeys, Maxime Vanmechelen, Brecht Decraene, Marleen Derweduwe, Nikolina Dubroja Lakic, Gautam Shankar, Madhu Nagathihalli Kantharaju, Jan Philipp Albrecht, Ilse Geudens, Fabio Stanchi, Keith L Ligon, Bram Boeckx, Diether Lambrechts, Kyle Harrington, Ludo Van Den Bosch, Steven De Vleeschouwer, Frederik De Smet, Holger Gerhardt

**Affiliations:** ^1^ Max Delbrück Center for Molecular Medicine in the Helmholtz Association Berlin Germany; ^2^ VIB ‐ KU Leuven Center for Cancer Biology VIB ‐ KU Leuven Leuven Belgium; ^3^ The Laboratory for Precision Cancer Medicine, Translational Cell and Tissue Research Unit, Department of Imaging & Pathology KU Leuven Leuven Belgium; ^4^ KU Leuven Institute for Single Cell Omics (LISCO) KU Leuven Leuven Belgium; ^5^ DZHK (German Center for Cardiovascular Research), Partner Site Berlin Berlin Germany; ^6^ Charité ‐ Universitätsmedizin Berlin Berlin Germany; ^7^ Department of Medical Oncology University Hospitals Leuven Leuven Belgium; ^8^ Laboratory of Experimental Neurosurgery and Neuroanatomy, Department of Neurosciences, KU Leuven & Leuven Brain Institute (LBI) KU Leuven Leuven Belgium; ^9^ Department of Neurosurgery University Hospitals Leuven Leuven Belgium; ^10^ Humboldt University of Berlin Berlin Germany; ^11^ Center for Neuro‐oncology Dana‐Farber Cancer Institute Boston MA USA; ^12^ Department of Pathology Brigham and Women's Hospital Boston MA USA; ^13^ Department of Pathology Harvard Medical School Boston MA USA; ^14^ Laboratory of Translational Genetics, Department of Human Genetics KU Leuven Leuven Belgium; ^15^ Chan Zuckerberg Initiative Redwood City CA USA; ^16^ Laboratory of Neurobiology, Department of Neurosciences, Experimental Neurology & Leuven Brain Institute (LBI) KU Leuven Leuven Belgium; ^17^ VIB ‐ KU Leuven Center for Brain & Disease Research, Laboratory of Neurobiology VIB ‐ KU Leuven Leuven Belgium; ^18^ Berlin Institute of Health Berlin Germany

**Keywords:** glioblastoma, *LGALS1*, macrophages, scRNA‐seq, zebrafish avatars, Cancer, Immunology

## Abstract

Glioblastoma (GBM) remains the most malignant primary brain tumor, with a median survival rarely exceeding 2 years. Tumor heterogeneity and an immunosuppressive microenvironment are key factors contributing to the poor response rates of current therapeutic approaches. GBM‐associated macrophages (GAMs) often exhibit immunosuppressive features that promote tumor progression. However, their dynamic interactions with GBM tumor cells remain poorly understood. Here, we used patient‐derived GBM stem cell cultures and combined single‐cell RNA sequencing of GAM‐GBM co‐cultures and real‐time *in vivo* monitoring of GAM‐GBM interactions in orthotopic zebrafish xenograft models to provide insight into the cellular, molecular, and spatial heterogeneity. Our analyses revealed substantial heterogeneity across GBM patients in GBM‐induced GAM polarization and the ability to attract and activate GAMs—features that correlated with patient survival. Differential gene expression analysis, immunohistochemistry on original tumor samples, and knock‐out experiments in zebrafish subsequently identified *LGALS1* as a primary regulator of immunosuppression. Overall, our work highlights that GAM‐GBM interactions can be studied in a clinically relevant way using co‐cultures and avatar models, while offering new opportunities to identify promising immune‐modulating targets.

## Graphical abstract

scRNA‐seq and live‐imaging in zebrafish avatars were used to study the complex and dynamic interplay between GBM tumor cells and GAMs, and provide fresh perspectives and opportunities for discovering promising immune‐modulating targets in GBM.

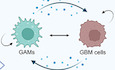

**The paper explained**

**Problem**

Glioblastoma (GBM) is the most common and aggressive type of brain tumors in adults. Despite significant scientific efforts, the treatment of GBM has not changed in over 15 years and GBM patients rarely survive more than 2 years. The major challenges of this disease include tumor heterogeneity and an immunosuppressive microenvironment, which contribute to the poor response rates of current therapeutic approaches. Macrophages are immune cells that normally protect the body against tumor formation, but GBM‐associated macrophages (GAMs) often exhibit immunosuppressive properties that promote tumor progression.

**Results**

To understand how GAMs interact with GBM tumor cells, we used patient‐derived GBM tumor cells for expression profiling and *in vivo* monitoring of GAM‐GBM interactions in zebrafish embryos. Our analyses revealed polarization of GAMs toward an immunosuppressive and tumor‐supportive phenotype. Interestingly, the degree of polarization was highly variable among patients and correlated with patient survival. Using expression profiling, we discovered heterogeneity in the molecular changes in GAMs induced by the different patient‐derived tumor cells, and we identified patient‐specific interaction patterns. We also performed high‐resolution live‐imaging in a zebrafish xenograft model to visualize the dynamic interactions between transplanted tumor cells and GAMs in real time. The time‐lapse movies illustrated tumor cell invasion and infiltration of reactive GAMs. We developed an image analysis pipeline to process the *in vivo* recordings, which was used to identify distinct behavioral patterns of tumor cells and GAMs. Ultimately, our work identified galectin‐1 (*LGALS1*) as a crucial regulator of immunosuppression, enabling GBM tumor cells to avoid being targeted by GAMs. Genetic depletion of *LGALS1* impaired GAMs to switch to an immunosuppressive phenotype in zebrafish, significantly reducing tumor invasiveness.

**Impact**

This study provides novel insights into the dynamic interactions between patient‐derived GBM tumor cells and GAMs and sheds light on potential approaches to repolarize GAMs toward a more anti‐tumorigenic state. Using our models, GAM‐GBM interactions can be studied in a clinically relevant manner, while providing new opportunities to discover promising immunomodulatory targets.

## Introduction

Glioblastoma (GBM) is the most common and aggressive type of malignant brain tumors in adults (Grochans *et al*, 2022). It is associated with a poor prognosis: a median survival of 15 months under standard of care (SoC) treatment, which comprises maximal safe surgical resection, followed by radiotherapy and chemotherapy (Stupp *et al*, 2005; Grochans *et al*, 2022). The main challenges presented by this malignancy are the cellular and molecular heterogeneity, the aggressive nature and invasive behavior, the inability to surgically resect the entire tumor, and the limitations of drug administration (Harder *et al*, 2018). Together, these problems invariably result in tumor progression or recurrence, demonstrating the urgent need for more effective treatments (Shergalis *et al*, 2018).

Cellular heterogeneity remains an important hallmark of GBM: it not only afflicts tumor cells, but is also present in cells of the tumor microenvironment (TME). GBM‐associated macrophages (GAMs) represent 30–50% of the tumor and consist of tissue‐resident microglia and tumor‐infiltrating macrophages. Nevertheless, it remains unclear how GAM behavior is affected by tumor cell heterogeneity and how this contributes to GBM progression (Reimunde *et al*, 2021). In general, immunosuppressive tumor‐associated macrophages (TAMs) are critical regulators of tumor progression, metastasis, and immune evasion, and therefore promising therapeutic targets (Buonfiglioli & Hambardzumyan, 2021; Wang *et al*, 2022a). In the last decade, therapeutic targeting of immune cells has gained great interest and impressive results have been achieved with immunotherapy in various types of cancer (Esfahani *et al*, 2020). However, GBM is a “cold” tumor and the limited success rate of immunotherapy relates to the highly immunosuppressive nature of the GBM microenvironment which is largely driven by GAMs and results in low levels of tumor‐infiltrating lymphocytes and T cell exhaustion (Yu & Quail, 2021). Despite significant scientific efforts, the SoC treatment for GBM has not changed for more than 15 years. Therefore, landscaping the levels and types of GAM polarization across the GBM patient population could shed light on novel approaches to repolarize GAMs to a more anti‐tumorigenic state, as such presenting an alternative approach to ultimately improve treatment for GBM patients (Wang *et al*, 2022b).

In recent years, the knowledge of GBM pathophysiology has advanced significantly and many promising research strategies have been pursued. The zebrafish (*Danio rerio*) xenograft model has proven to be a versatile animal model ideally suited for cancer research (Chen *et al*, 2021b). Furthermore, proof‐of‐concept studies have suggested that zebrafish xenograft models can serve as a preclinical drug screening platform and open the possibility to guide personalized treatments (Zon & Peterson, 2005; Veinotte *et al*, 2014; di Franco *et al*, 2022). Moreover, most relevant brain regions and the blood–brain barrier (BBB) are highly conserved and thus have a similar structure compared to humans. The high physiological conservation and the simplicity of xenotransplantation of human cancer cells into zebrafish embryos have recently enabled researchers to recapitulate the characteristics of GBM, including the TME (Reimunde *et al*, 2021). However, studies that investigate the dynamic interactions between patient‐derived GBM tumor cells and GAMs through long‐term real‐time recordings are currently lacking, though these insights are crucial to understand how GAMs influence disease progression.

In this work, we leverage a combination of single‐cell RNA sequencing (scRNA‐seq) of *in vitro* GAM‐GBM co‐cultures and real‐time *in vivo* monitoring of GAM‐GBM interactions in orthotopic zebrafish xenograft models to map cellular, molecular, and spatial heterogeneity of GBM and associated macrophages. We extensively characterize a set of eight patient‐derived GBM stem cell cultures (PD‐GSCCs, hereafter referred to as GSCC in short) with different genomic and transcriptomic profiles, in an *in vitro* co‐culture model with human macrophages. Using scRNA‐seq, we report the heterogeneity in molecular changes in the macrophages influenced by the various GSCCs and identify patient‐specific interaction patterns. We also show the use of high‐resolution live‐imaging in an orthotopic zebrafish xenograft model to visualize the dynamic interactions between transplanted tumor cells and GAMs in real time. We developed an image analysis pipeline to process *in vivo* recordings, which was used to identify distinct behavioral patterns of GSCCs and GAMs. The time‐lapse movies reveal tumor cell invasion and infiltration of reactive GAMs and how this varies across patients. Ultimately, using differential gene expression (DGE) analysis, immunohistochemistry (IHC) profiling of original tumor samples, and knock‐out (KO) experiments, our work identifies galectin‐1 (GAL1, *LGALS1*) as an important immunomodulating target that affects tumor growth.

## Results

### Experimental set‐up: studying the interaction between macrophages/GAMs and GSCCs in model systems

**Figure 1:**
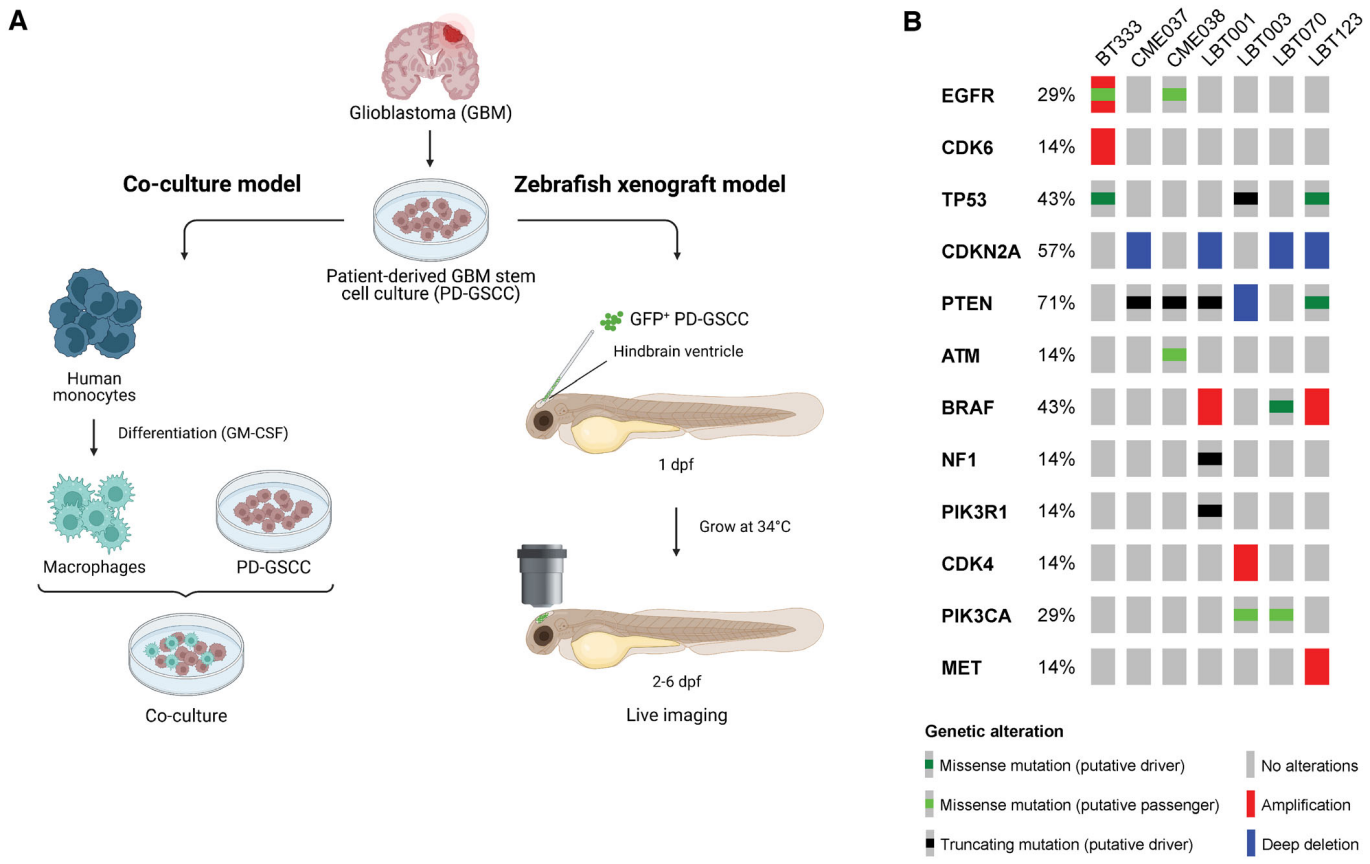
Overview experimental set‐up and included GSCCs Schematic overview of the study design. Eight different GSCCs were used in a co‐culture model with human monocyte‐derived macrophages (left) and in an orthotopic zebrafish xenograft model (right). dpf, days post fertilization.Oncoprint of included GSCCs: broad spectrum of common genetic aberrations in GBM were covered. Included GSCCs are mentioned on top of the figure.

To analyze the cellular and molecular interaction of macrophages/GAMs and GSCCs, we made use of both an *in vitro* and *in vivo* model (Fig 1A). First, cellular interactions were studied in a co‐culture model using scRNA‐seq. Human monocytes were isolated from the blood of healthy volunteers, differentiated into macrophages, and co‐cultured with eight different GSCCs, originating from seven different GBM patients. For one patient, we included two paired GSCCs (CME037/CME038), generated from a sample at initial diagnosis and recurrence. Importantly, the GSCCs used for these experiments cover a broad spectrum of genetic aberrations that are frequently present in GBM (Fig 1B): GSCCs were IDH1‐wildtype with mutations and/or copy number variations in common tumor suppressors and oncogenes such as EGFR^amp/mut^/Chr7^amp^, CDKN2A^del^, TP53^mut^, PTEN^mut/del^/Chr10^del^, CDK4^amp^, BRAF^amp/mut^ and NF1^mut^. We performed OneSeq analysis (Agilent) combining focused exome sequencing of the 43 most mutated genes in GBM with genome‐wide copy number variation analysis of the GSCCs, and identified both shared and GSCC‐specific mutations, consistent with previously reported intertumoral genomic heterogeneity of GBM (Rosenberg *et al*, 2017).

Next, we optimized an orthotopic zebrafish xenograft model (hereafter referred to as zebrafish avatar model) in which GFP^+^ GSCCs were engrafted into a macrophage reporter line (see Materials and Methods, Fig 1A). This was achieved by injecting GFP^+^ GSCCs in the hindbrain ventricle of zebrafish embryos at 30 h post fertilization (hpf). Zebrafish avatars were subsequently grown at an elevated temperature of 34°C (as compared to 28°C at which zebrafish are normally grown) to allow normal development of the embryos, while maintaining an environment that also supports tumor cell proliferation. Dynamic interactions between GAMs and GSCCs were eventually captured in time‐lapse movies by performing live‐imaging of the zebrafish embryos.

### Single‐cell profiling of co‐cultures of GSCCs and macrophages

**Figure 2:**
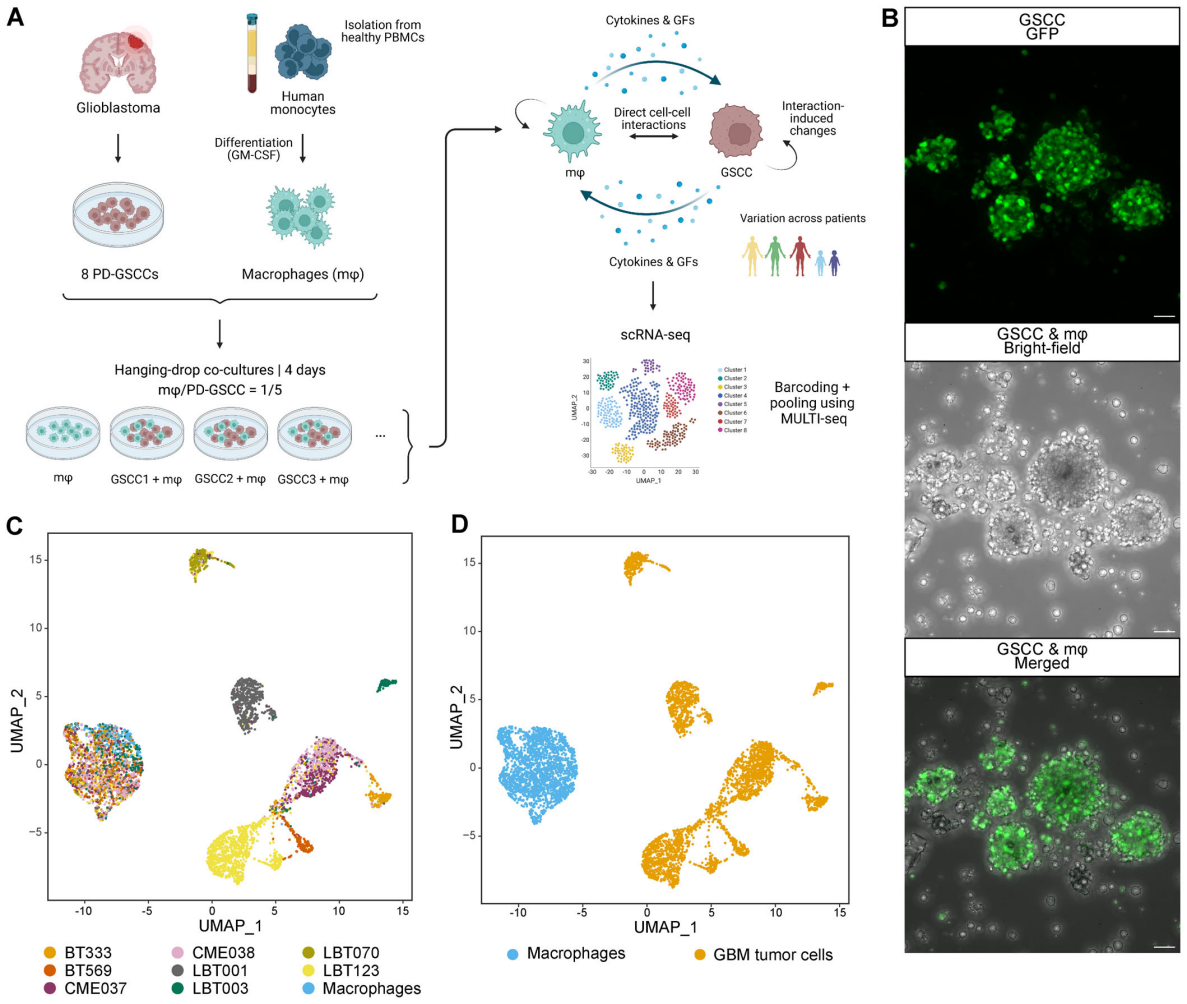
Single‐cell profiling of GSCC‐macrophage co‐cultures ASchematic overview of the experimental set‐up of the scRNA‐seq profiling assay. PBMCs, peripheral blood mononuclear cells; GFs, growth factors.BHigh‐resolution images of a GSCC‐macrophage co‐culture. GFP^+^ GSCC was used for visualization of the co‐culture with non‐labeled macrophages. Scale bars: 50 μm.C, DUniform Manifold Approximation and Projection (UMAP) plots of 5,320 cells from nine samples, annotated by sample name (C) and by cell type (D). Source data are available online for this figure.

**Figure EV1:**
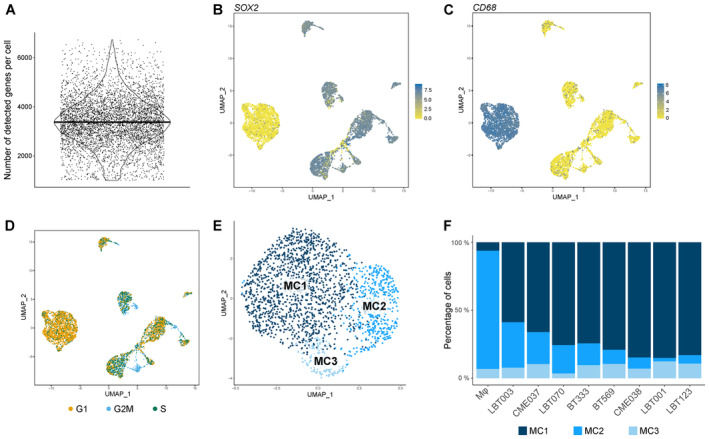
Single‐cell profiling of GSCC‐macrophage co‐cultures reveals molecular heterogeneity of GBM‐associated macrophages AViolin plot showing the number of detected genes per cell (*n* = 5,320 cells from nine samples with a median of 3,334 genes detected per cell).B, CUMAP plots showing *SOX2* (B) and *CD68* (C) expression.DUMAP plot showing cell cycle score. Proliferating GBM tumor cells are depicted in blue.EUMAP plot of macrophage population identified three distinct macrophage subclusters (MC1‐3).FMacrophage subcluster distribution for the different samples. m*φ*, macrophages. Source data are available online for this figure.

To investigate how GSCCs influence the phenotypic features of macrophages in a patient‐specific manner, we established an *in vitro* co‐culture system between GSCCs and human monocyte‐derived macrophages (Fig 2A). Macrophages and GSCCs were co‐cultured in a 1:5 ratio using the hanging‐drop method (Keller, 1995; Foty, 2011), while hanging drops of only macrophages were used as a control. After 4 days, cells of the various conditions were collected, dissociated, labeled using the MULTI‐seq methodology (McGinnis *et al*, 2019) and pooled for scRNA‐seq. Subsequent sequencing and demultiplexing yielded a total of 5,320 cells from nine samples (8 co‐cultures + 1 monoculture of macrophages, 218–1,182 cells per sample, Table EV1) that passed quality control thresholds (see Materials and Methods), with a median of 3,334 genes detected per cell (Fig EV1A). Dimensionality reduction and unsupervised clustering (see Materials and Methods) revealed a clear separation between GSCCs and macrophages, with sample‐specific clustering of the GSCCs and co‐clustering of all macrophages (Fig 2C and D). As anticipated, the paired GSCCs (CME037/CME038) clustered together. Each cluster was identified as GBM tumor cells or macrophages based on the expression of *SOX2* and *CD68*, respectively (Fig EV1B and C). Proliferating GBM tumor cells were identified based on their cell cycle score (Fig EV1D).

### Molecular heterogeneity of GBM‐associated macrophages

**Figure 3:**
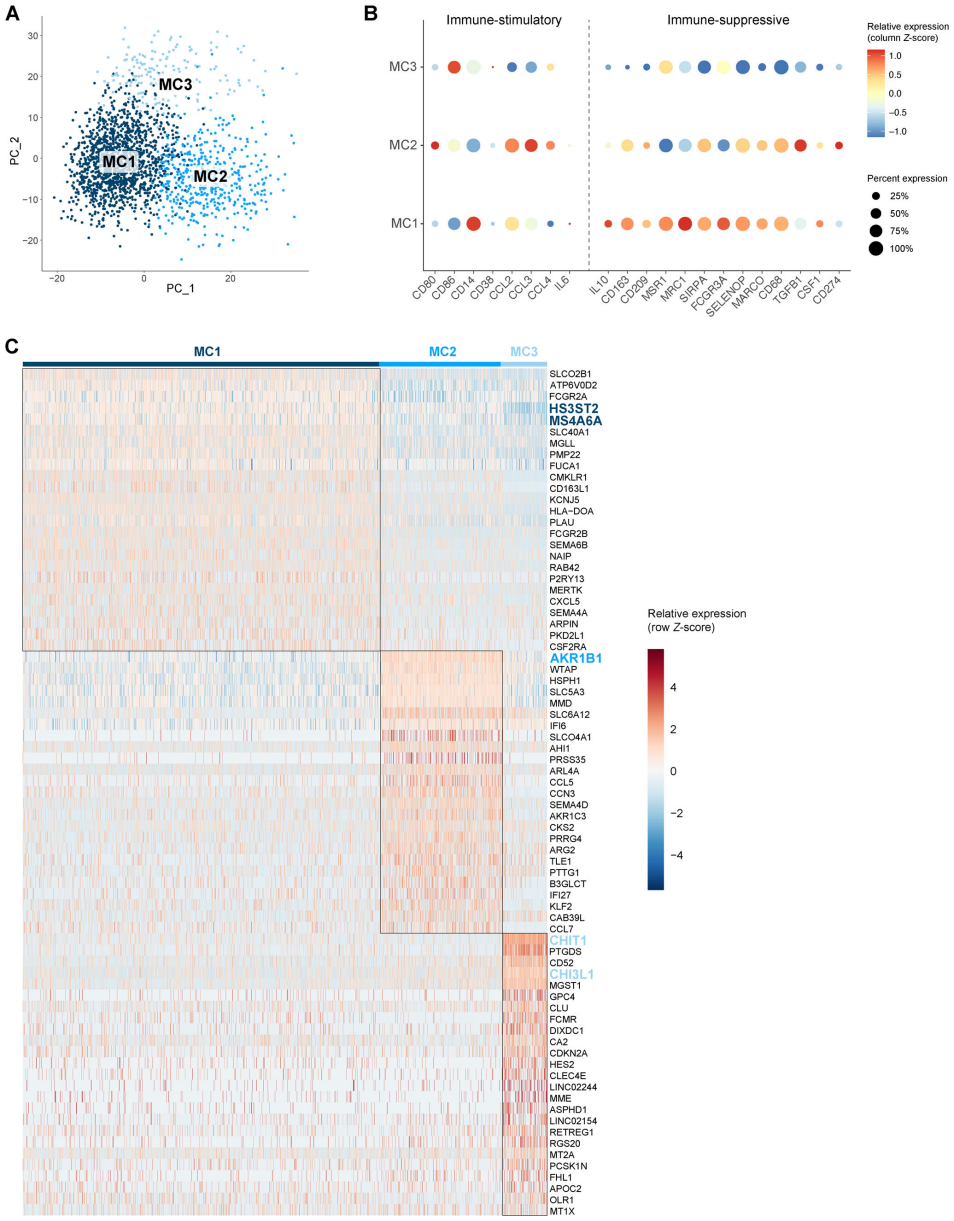
Molecular heterogeneity of GBM‐associated macrophages Principal component analysis (PCA) plot of macrophage population identified three distinct macrophage subclusters (MC1‐3). PCs were calculated using the 2,000 most variable genes. Plot shows PC1 and PC2.Dot plot showing marker gene expression for immune‐stimulation and ‐suppression. Dot size indicates the percentage of cells in each macrophage subcluster expressing the gene, and dot color indicates the relative expression level.Heatmap of top 25 differentially expressed genes in the macrophage subclusters, ranked by log_2_(FC). Genes discussed in the text are highlighted in the subcluster colors. Source data are available online for this figure.

Glioblastoma‐associated macrophages can represent up to 50% of GBM tumors. To elucidate the molecular heterogeneity of the monocyte‐derived macrophages in the co‐culture model, we performed unsupervised subclustering of the macrophages, yielding three clusters (MC1‐3) (Figs 3A and EV1E). All co‐cultures contributed to each macrophage cluster (Fig EV1F). As reported by others, the observed macrophage subtypes did not fit the classical, yet outdated, M1/M2 macrophage paradigm, introduced by Mills *et al* (2000) (Müller *et al*, 2017). There was limited analogy between well‐known M1 and M2 markers and macrophage cluster signature genes. However, the expression levels of well‐known markers for immune‐stimulation and ‐suppression were investigated, where we found that MC1 macrophages had a more immunosuppressive nature, while MC2 macrophages exhibited more immune‐stimulatory features (Fig 3B).

Next, we manually annotated the distinct macrophage subtypes using marker gene analysis (Fig 3C). MC1 consisted of macrophages that expressed high levels of immunosuppressive genes *HS3ST2* and *MS4A6A*. *HS3ST2* has previously been shown to be highly expressed upon alternative stimulation (M2 polarization), while the enzyme was not detected in pro‐inflammatory macrophages and primary monocytes (Martinez *et al*, 2015). Interestingly, *MS4A6A* is expressed in M2 macrophages and correlates with macrophage infiltration, unfavorable clinical outcome, and poor responses to adjuvant chemotherapy in glioma patients (Zhang *et al*, 2022). MC2 contained macrophages that express *AKR1B1* (Fig 3C), which is commonly associated with an inflammatory profile (Erbel *et al*, 2016; Cheng *et al*, 2021). Of note, cells in MC2 mainly originated from the macrophage monoculture and represent cells that were polarized toward an inflammatory subtype induced by treatment with granulocyte‐macrophage colony‐stimulating factor (GM‐CSF) (Lofti *et al*, 2020). MC3 macrophages expressed high levels of *CHIT1* (and *CHI3L1*) and lower levels of the mature macrophage markers *CD68*, *CD163*, *CD204/MSR1*, and *CD206/MRC1* (Fig 3B and C). As such, we identified MC3 as transitioning monocytes (TransMos) as it has been demonstrated that *CHIT1* expression increases exponentially over time in monocytes during the transition to macrophages (di Rosa *et al*, 2013).

### Macrophages shift toward an immunosuppressive phenotype upon co‐culture with patient‐derived GSCCs

**Figure 4:**
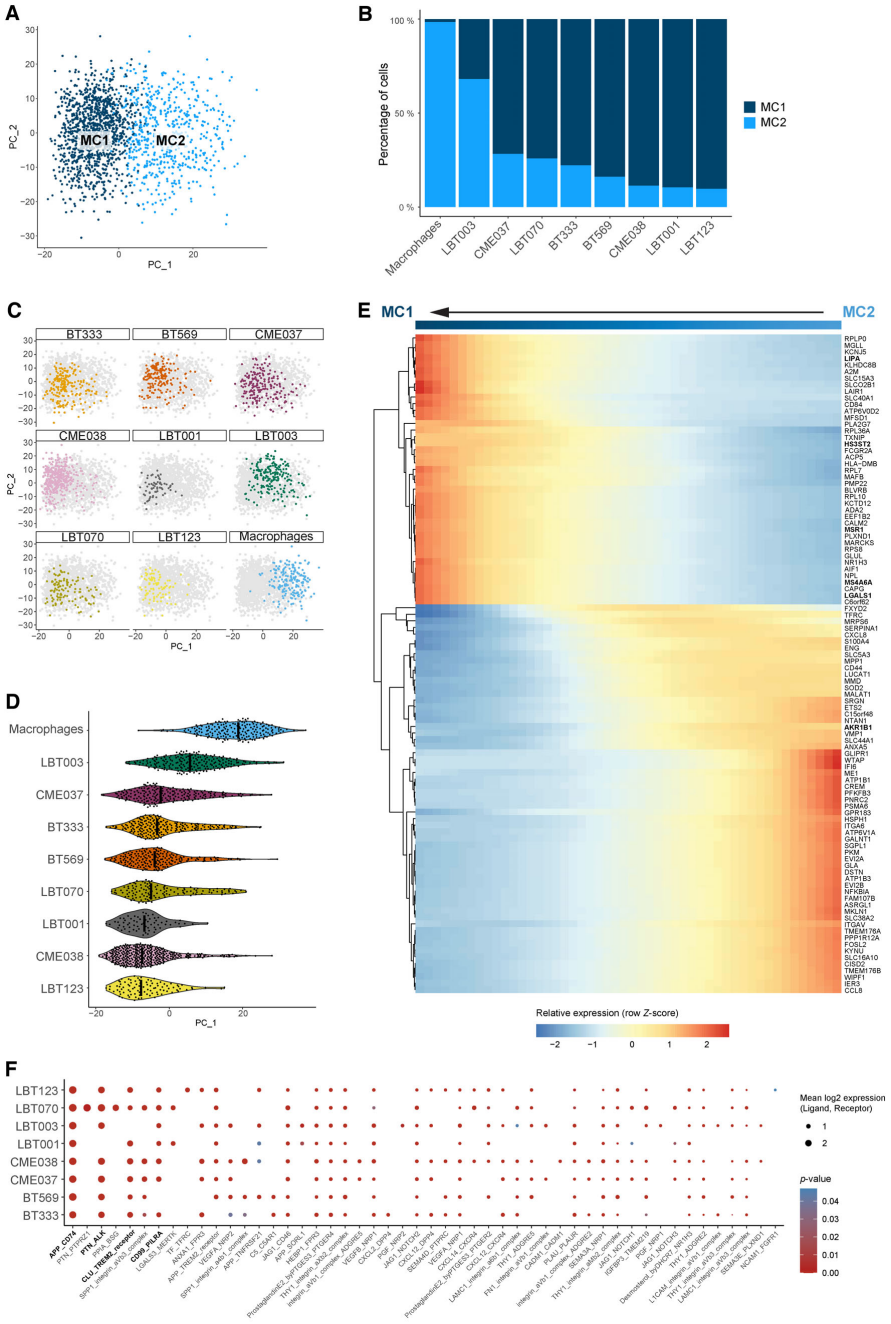
Macrophages shift toward an immunosuppressive phenotype upon co‐culture with patient‐derived GSCCs PCA plot of macrophage population without TransMos shows two distinct macrophage subclusters (MC1‐2). PCs were calculated using the 2,000 most variable genes. PC1 and PC2 are shown on the PCA plot.Macrophage subcluster distribution for the different samples.Representation of original samples on the PCA plot.Pseudotime analysis along PC1 axis. Violin plots depicting the PC1 values of each single cell, split up by sample.Heatmap of the 100 most significant temporally expressed genes along the PC1 axis, constructed by fitting a generalized additive model with the PC1 value as a LOESS term (see Materials and Methods).Dot plot of cell–cell communication analysis using CellPhoneDB. Depicted are L:R pairs for GSCC ‐ macrophage signaling across all GSCCs, ranked by mean log_2_ expression. Each dot size shows the log_2_ mean of expression values and dot color indicates the *P*‐value for the listed L:R pairs (*x*‐axis) in the respective GSCCs (*y*‐axis). Only top 50 significant L:R pairs, with cut‐offs of *P*‐value ≦ 0.05 are shown. The *P*‐values were generated by CellPhoneDB, which uses a one‐sided permutation test to compute significant interactions. Source data are available online for this figure.

**Figure EV2:**
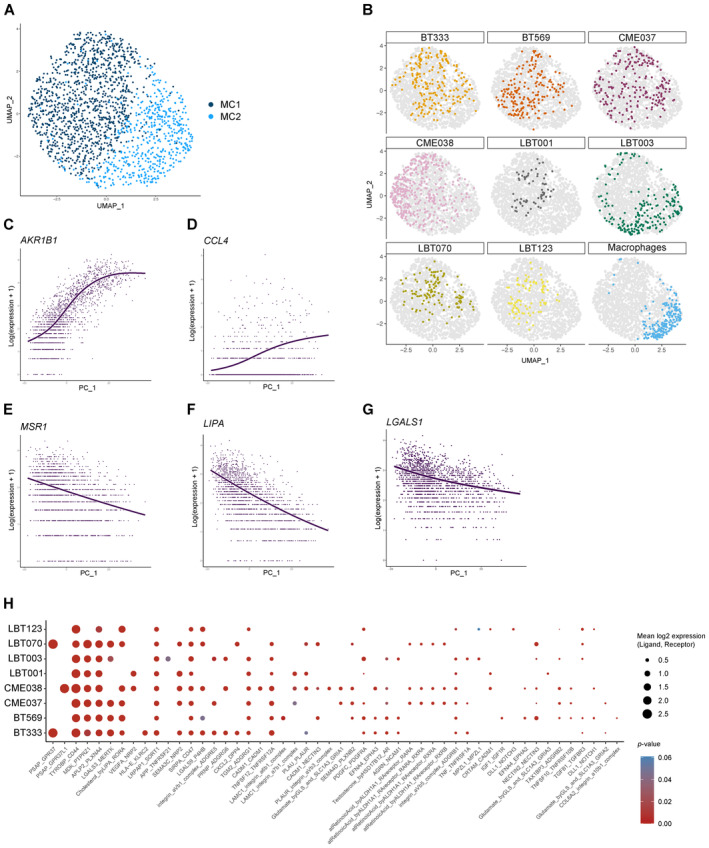
Macrophages shift toward an immunosuppressive phenotype upon co‐culture with patient‐derived GSCCs AUMAP plot of macrophage population without TransMos shows two distinct macrophage subclusters (MC1‐2).BRepresentation of original samples on the UMAP plot.C–GLOESS plots for *AKR1B1* (C), *CCL4* (D), *MSR1* (E), *LIPA* (F), and *LGALS1* (G).HDot plot of cell–cell communication analysis using CellPhoneDB. Depicted are L:R pairs for macrophage ‐ GSCC signaling across all GSCCs, ranked by mean log_2_ expression. Each dot size shows the log_2_ mean of expression values and dot color indicates the *P*‐value for the listed L:R pairs (*x*‐axis) in the respective GSCCs (*y*‐axis). Only top 50 significant L:R pairs, with cut‐offs of *P*‐value ≦ 0.05 are shown. The *P*‐values were generated by CellPhoneDB, which uses a one‐sided permutation test to compute significant interactions. Source data are available online for this figure.

We then sought to characterize the molecular switch in macrophages induced by interactions with GSCCs by performing pseudotime analysis. MC3 was not included in the analysis because we aimed to capture the interaction between mature macrophages and GSCCs, rather than the interaction between TransMos and GSCCs (Fig EV2A). The bulk of the variance between MC1 and MC2 is explained by principal component (PC) 1 (Fig 4A). The macrophage subcluster distribution across the GSCCs indicated that macrophages switch to an immunosuppressive state upon co‐culture with GSCCs (MC2 to MC1, Fig 4B). The macrophage monoculture mainly consisted of inflammatory MC2 macrophages, induced by treatment with GM‐CSF. Upon co‐culturing, the proportion of MC2 macrophages significantly decreased for all GSCCs (Chi‐square test, *P* < 1.0e‐15). Surprisingly, co‐cultures of LBT003 contained significantly more pro‐inflammatory MC2 macrophages and less immunosuppressive MC1 macrophages than co‐cultures of other GSCCs (Chi‐square test, *P* < 1.0e‐15) (Fig 4B).

Next, we used each cell's PC1 value as a measure for pseudotime along the MC2‐MC1 trajectory and considered MC2 the root of the trajectory (Fig 4C and D). Principal component analysis (PCA) and Uniform Manifold Approximation and Projection (UMAP) plots per sample showed a gradient in the macrophage population (Figs 4C and EV2B), indicating that macrophages were in the process of a phenotypic switch. Interestingly, of all samples, macrophages co‐cultured with LBT003 most closely resembled the control macrophages (Fig 4C and D). To study which genes were involved in the phenotypic shift, we identified temporally expressed genes along the PC1 axis (from MC2 to MC1) by fitting a generalized additive model with the PC1 value as a LOESS term (see Materials and Methods, Fig 4E). We found that the expression of immunosuppressive genes increased along the MC2‐MC1 trajectory, while the expression of immunostimulatory genes gradually decreased, thereby triggering a phenotypic change. For instance, immunostimulatory genes like *AKR1B1* and *CCL4* gradually decreased in expression along the trajectory (Fig EV2C and D). *MSR1*, *LIPA*, and *LGALS1*, genes that have previously been shown to be upregulated in immunosuppressive macrophages (Huang *et al*, 2014), were initially downregulated and increased in expression along the trajectory (Fig EV2E–G).

To investigate whether potential ligand:receptor (L:R) interactions between GSCCs and macrophages could explain the observed macrophage polarization, we performed CellPhoneDB analysis (Fig 4F) (Garcia‐Alonso *et al*, 2022). This revealed several strong putative interactions between GBM tumor cell ligands and macrophage receptors that were conserved over the different co‐cultures, including APP:CD74, CD99:PILRA, PTN:ALK, and CLU:TREM2. CD74 signaling, which was found to be significant in all co‐cultures, has previously been shown to induce an inflammatory macrophage phenotype (Zeiner *et al*, 2015). In contrast, TREM2 signaling induces a strong immunosuppressive phenotype and elevated TREM2 expression in macrophages is considered a bad prognostic marker in several pathologies (Katzenelenbogen *et al*, 2020; Yu *et al*, 2023). Interestingly, putative CLU:TREM2 signaling was not significant for LBT003, which induced the weakest macrophage polarization in the co‐culture model, suggesting that the lack of this interaction might in part explain the limited polarization. When investigating predicted macrophage to GSCC signaling, several conserved putative interactions were found, including APLP2:PLXNA4, LRPAP1:SORT1, MDK:PTPRZ1, SIRPA:CD47, and TYROBP:CD44 (Fig EV2H). Of note, CD47, which is known to be upregulated in glioma stem cells, binds to SIRPα on the surface of macrophages to exert a “don't eat me” signal, thus contributing to immune evasion of the tumor cells by preventing phagocytosis by macrophages (Gholamin *et al*, 2017; Hu *et al*, 2020).

In general, we found that the interaction between GSCCs and GAMs induced a shift to a more immunosuppressive phenotype, but the magnitude of this shift was variable across GSCCs. Strikingly, CME037 and CME038, which are derived from the same patient at initial diagnosis and recurrence, respectively, were positioned at opposite ends of the spectrum, with the recurrent GSCC exhibiting a more immunosuppressive polarization. Furthermore, we showed multiple predicted interactions between GSCCs and macrophages that could govern macrophage polarization or immune evasion, some of which were conserved over all co‐cultures, while others were specific to certain cultures.

### Mapping the real‐time evolution of GSCCs and GAMs in zebrafish avatars

**Figure 5:**
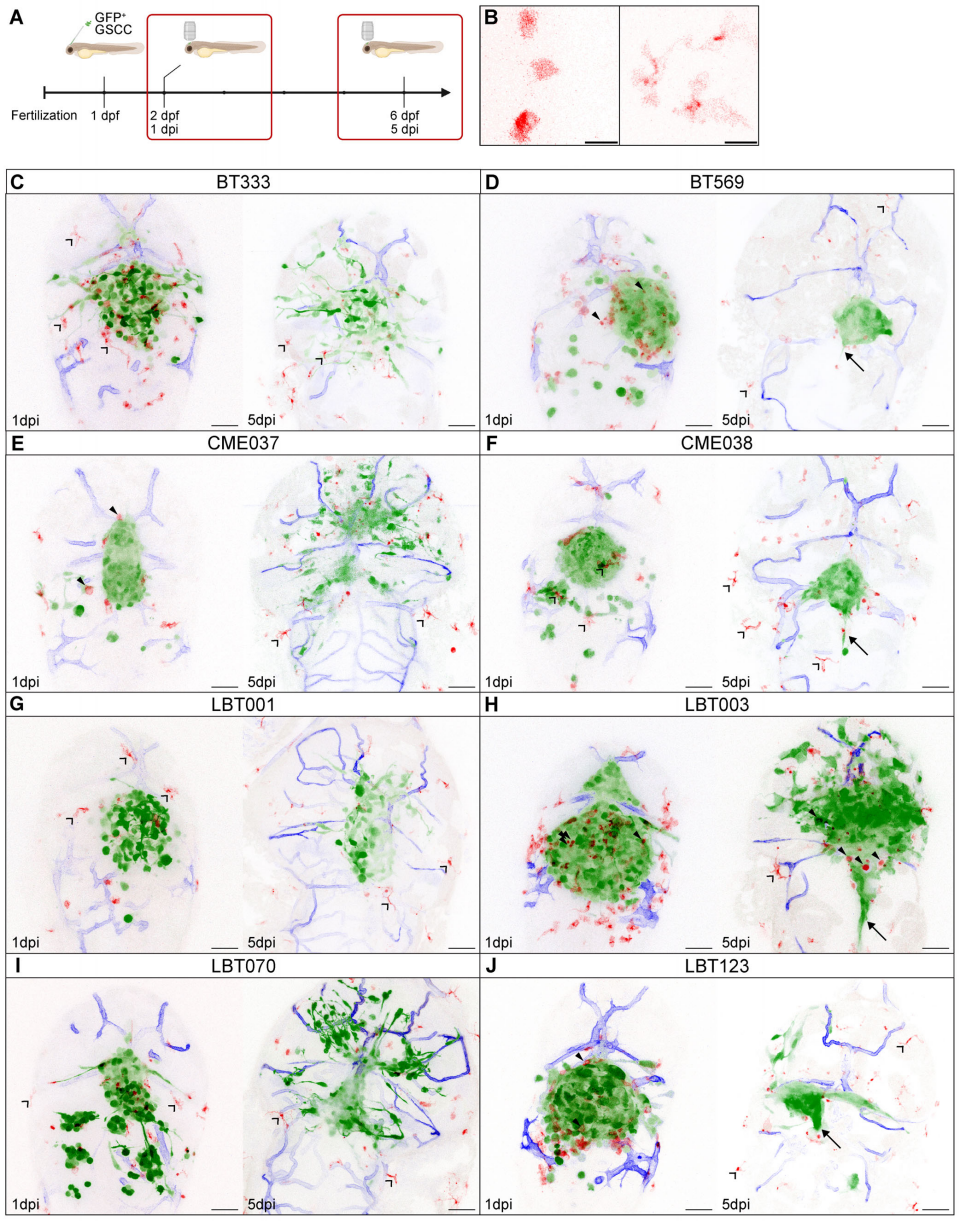
Overview of different zebrafish avatars ASchematic overview showing the timeline of the orthotopic zebrafish xenograft model.BZoomed‐in images of round (left) and ramified (right) GAMs in the recorded time‐lapse movies. Scale bars: 15 μm.C–JRepresentative maximum intensity projections of a z stack of the head region of *Tg(mpeg1:mCherryF)* ^*ump2*^; *Tg(kdrl:lynEYFP)* ^*md77*^ zebrafish embryos with different GFP‐labeled patient‐derived GSCC tumors, at 1 dpi (left panel) and 5 dpi (right panel): BT333 (C), BT569 (D), CME037 (E), CME038 (F), LBT001 (G), LBT003 (H), LBT070 (I), LBT123 (J). GBM tumor cells are shown in green, GAMs in red, and blood vessels in blue. Arrows indicate midline protrusion of the tumor. Filled arrowheads indicate round GAMs. Open arrowheads indicate ramified GAMs. Scale bars: 50 μm.

**Figure 6:**
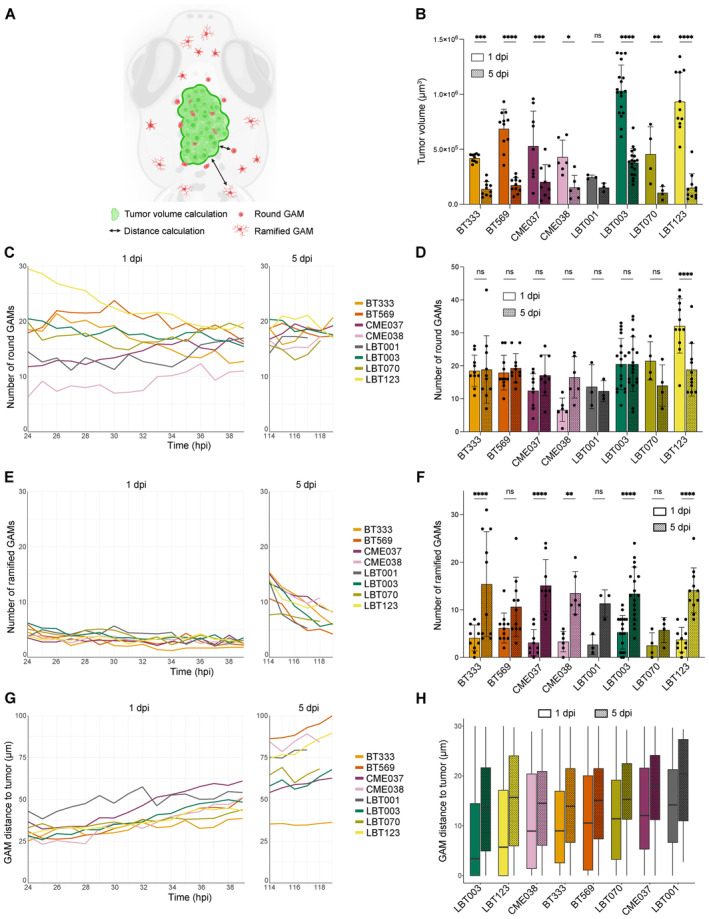
GSCC‐specific morphometrics and dynamics of the tumor and its microenvironment in 3D over time Schematic overview of features extracted and computed from the time‐lapse movies of the orthotopic zebrafish xenograft model.Tumor volume at the start of 1 and 5 dpi time‐lapse movies (*n* = 10 (BT333), 11 (BT569), 9 (CME037), 6 (CME038), 3 (LBT001), 18 (LBT003), 4 (LBT070), 11 (LBT123) zebrafish embryos; *P* = 0.0005 (BT333), 1.2e‐10 (BT569), 0.0001 (CME037), 0.0147 (CME038), 0.9905 (LBT001), < 1.0e‐15 (LBT003), 0.0099 (LBT070), < 1.0e‐15 (LBT123)).Mean number of round GAMs over time, during 1 dpi movies (left) and 5 dpi movies (right) (*n* = 10 (BT333), 12 and 11 (BT569; 1 and 5 dpi), 10 and 9 (CME037; 1 and 5 dpi), 6 (CME038), 8 and 4 (LBT001; 1 and 5 dpi), 22 and 18 (LBT003; 1 and 5 dpi), 12 and 9 (LBT070; 1 and 5 dpi), 15 and 11 (LBT123; 1 and 5 dpi) zebrafish embryos, see also Fig EV3A).Number of round GAMs at the start of 1 and 5 dpi time‐lapse movies (*n* = 10 (BT333), 11 (BT569), 9 (CME037), 6 (CME038), 3 (LBT001), 18 (LBT003), 4 (LBT070), 11 (LBT123) zebrafish embryos; *P* = 1.0000 (BT333), 0.9996 (BT569), 0.6715 (CME037), 0.0804 (CME038), 1.0000 (LBT001), 1.0000 (LBT003), 0.5888 (LBT070), 7.3e‐5 (LBT123)).Mean number of ramified GAMs over time, during 1 dpi movies (left) and 5 dpi movies (right) (*n* = 10 (BT333), 12 and 11 (BT569; 1 and 5 dpi), 10 and 9 (CME037; 1 and 5 dpi), 6 (CME038), 8 and 4 (LBT001; 1 and 5 dpi), 22 and 18 (LBT003; 1 and 5 dpi), 12 and 9 (LBT070; 1 and 5 dpi), 15 and 11 (LBT123; 1 and 5 dpi) zebrafish embryos, see also Fig EV3A).Number of ramified GAMs at the start of 1 and 5 dpi time‐lapse movies (*n* = 10 (BT333), 11 (BT569), 9 (CME037), 6 (CME038), 3 (LBT001), 18 (LBT003), 4 (LBT070), 11 (LBT123) zebrafish embryos; *P* = 4.7e‐5 (BT333), 0.3089 (BT569), 4.1e‐5 (CME037), 0.0081 (CME038), 0.2899 (LBT001), 9.2e‐5 (LBT003), 0.9758 (LBT070), 9.4e‐5 (LBT123)).Mean GAM distance to the tumor over time, during 1 dpi movies (left) and 5 dpi movies (right) (*n* = 388 and 494 (BT333; 1 and 5 dpi), 503 and 471 (BT569; 1 and 5 dpi), 234 and 455 (CME037; 1 and 5 dpi), 107 and 251 (CME038; 1 and 5 dpi), 226 and 169 (LBT001; 1 and 5 dpi), 945 and 934 (LBT003; 1 and 5 dpi), 453 and 338 (LBT070; 1 and 5 dpi), 774 and 543 (LBT123; 1 and 5 dpi) GAMs).Boxplot of GAM distance to the tumor of all GAMs within 30 μm of the tumor, at the start of 1 and 5 dpi time‐lapse movies, ranked by increasing median distance at 1 dpi (*n* = 634 and 302 (LBT003; 1 and 5 dpi), 458 and 115 (LBT123; 1 and 5 dpi), 66 and 48 (CME038; 1 and 5 dpi), 267 and 264 (BT333; 1 and 5 dpi), 309 and 98 (BT569; 1 and 5 dpi), 257 and 99 (LBT070; 1 and 5 dpi), 134 and 142 (CME037; 1 and 5 dpi), 109 and 26 (LBT001; 1 and 5 dpi) GAMs; boxes stand for 50% of the data and minima/maxima are indicated by the line ends). Data information: Data describe biological replicates, i.e. individual zebrafish embryos. The xenograft experiment was replicated once for CME038, twice for BT333, BT569, CME037, LBT001, three times for LBT070 and LBT123, and four times for LBT003. In (B, D and F), data are presented as mean ± SD. The *P*‐values were calculated by two‐way repeated measures ANOVA, followed by Šídák's multiple comparisons correction. ns ≥ 0.05, *P < 0.05, **P < 0.01, ***P < 0.001, ****P < 0.0001. Source data are available online for this figure.

**Figure EV3:**
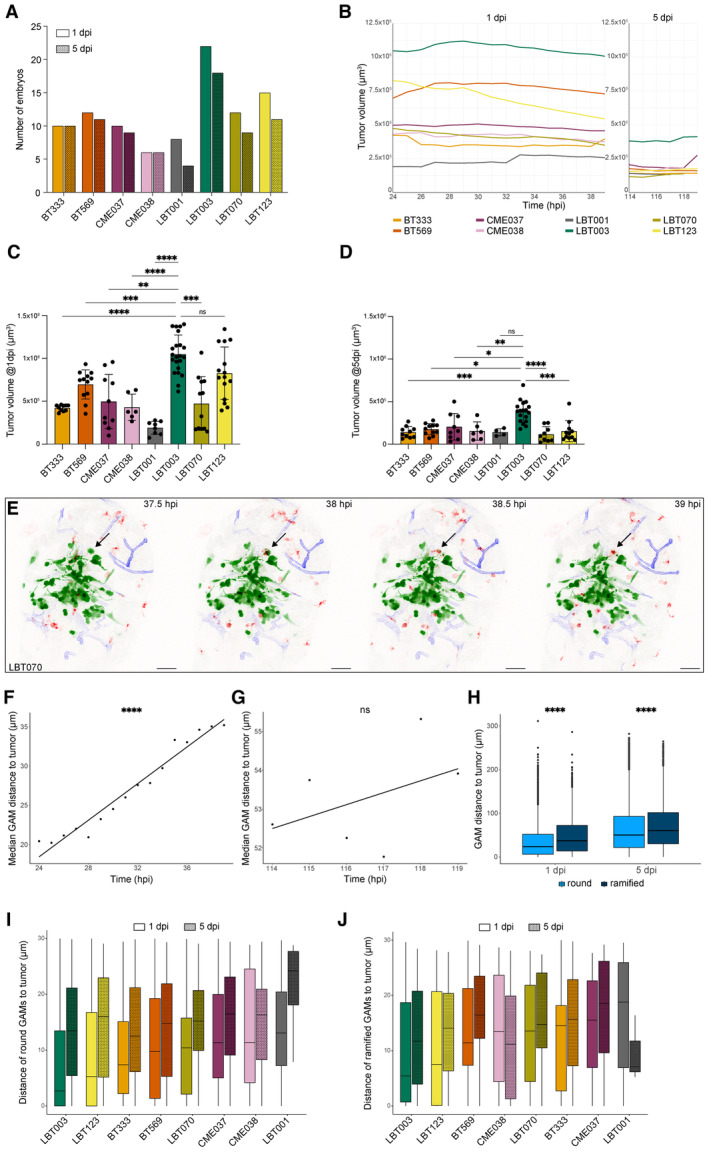
GSCC‐specific morphometrics and dynamics of the tumor and its microenvironment in 3D over time ANumber of zebrafish embryos used for the time‐lapse movies that were generated at 1 and 5 dpi.BMean tumor volume over time, during 1 dpi movies (left) and 5 dpi movies (right) (*n* = 10 (BT333), 12 and 11 (BT569; 1 and 5 dpi), 10 and 9 (CME037; 1 and 5 dpi), 6 (CME038), 8 and 4 (LBT001; 1 and 5 dpi), 22 and 18 (LBT003; 1 and 5 dpi), 12 and 9 (LBT070; 1 and 5 dpi), 15 and 11 (LBT123; 1 and 5 dpi) zebrafish embryos, see also panel A).CTumor volume at the start of 1 dpi time‐lapse movies (*n* = 10 (BT333), 12 (BT569), 10 (CME037), 6 (CME038), 8 (LBT001), 22 (LBT003), 12 (LBT070), 15 (LBT123); *P* = < 1.0e‐15 (BT333), 0.0001 (BT569), 0.0017 (CME037), 3.1e‐5 (CME038), < 1.0e‐15 (LBT001), 0.0002 (LBT070), 0.1542 (LBT123)).DTumor volume at the start of 5 dpi time‐lapse movies (*n* = 10 (BT333), 11 (BT569), 9 (CME037), 6 (CME038), 4 (LBT001), 18 (LBT003), 9 (LBT070), 11 (LBT123); *P* = 0.0009 (BT333), 0.0179 (BT569), 0.0237 (CME037), 0.0095 (CME038), 0.0538 (LBT001), 4.7e‐5 (LBT070), 0.0003 (LBT123)).ERepresentative maximum intensity projections of a z stack of the head region of a *Tg(mpeg1:mCherryF)* ^*ump2*^; *Tg(kdrl:lynEYFP)* ^*md77*^ zebrafish embryo with a GFP‐labeled LBT070 tumor, at 37.5, 38, 38.5 and 39 hpi to illustrate phagocytosis of a GBM tumor cell by a round GAM (indicated by arrows). GBM tumor cells are shown in green, GAMs in red, and blood vessels in blue. Scale bars: 50 μm.F, GTrend line of median GAM distance to the tumor over time in 1 dpi (F) and 5 dpi (G) movies for all GSCCs (*n* = 43,857 (F) and 16,639 (G) GAMs; *P* = 6.0e‐7 (F) and 0.7071 (G)).HBoxplot of distance of round and ramified GAMs to the tumor for all GSCCs, at the start of 1 and 5 dpi time‐lapse movies (*n* = 1,821 and 1,429 (round; 1 and 5 dpi), 420 and 1,008 (ramified; 1 and 5 dpi) GAMs; *P* < 6.4e‐7 (1 dpi) and < 2.4e‐5 (5 dpi); boxes stand for 50% of the data and minima/maxima are indicated by the line ends).IBoxplot of GAM distance to the tumor of round GAMs within 30 μm of the tumor, at the start of 1 and 5 dpi time‐lapse movies, ranked by increasing median distance at 1 dpi (*n* = 325 and 146 (LBT003; 1 and 5 dpi), 273 and 44 (LBT123; 1 and 5 dpi), 134 and 105 (BT333; 1 and 5 dpi), 141 and 49 (BT569; 1 and 5 dpi); 137 and 48 (LBT070; 1 and 5 dpi), 74 and 56 (CME037; 1 and 5 dpi), 26 and 26 (CME038; 1 and 5 dpi), 61 and 11 (LBT001; 1 and 5 dpi); boxes stand for 50% of the data and minima/maxima are indicated by the line ends).JBoxplot of GAM distance to the tumor of ramified GAMs within 30 μm of the tumor, at the start of 1 and 5 dpi time‐lapse movies, ranked by increasing median distance at 1 dpi (*n* = 63 and 51 (LBT003; 1 and 5 dpi), 26 and 26 (LBT123; 1 and 5 dpi), 38 and 21 (BT569; 1 and 5 dpi), 13 and 12 (CME038; 1 and 5 dpi); 29 and 13 (LBT070; 1 and 5 dpi), 25 and 86 (BT333; 1 and 5 dpi), 18 and 30 (CME037; 1 and 5 dpi), 15 and 3 (LBT001; 1 and 5 dpi); boxes stand for 50% of the data and minima/maxima are indicated by the line ends). Data information: Data describe biological replicates, i.e. individual zebrafish embryos. The xenograft experiment was replicated once for CME038, twice for BT333, BT569, CME037, LBT001, three times for LBT070 and LBT123, and four times for LBT003. In (C and D), data are presented as mean ± SD. In (C), the *P*‐values were calculated by Welch's ANOVA test, followed by Dunnett's T3 multiple comparisons correction. In (D), the *P*‐values were calculated by Kruskal–Wallis test, followed by Dunn's multiple comparisons correction. In (F and G), the *P*‐values were calculated by the Mann–Kendall trend test. In (H), the *P*‐values were calculated by Welch's ANOVA test, followed by Games‐Howell's multiple comparisons correction. ns ≥ 0.05, *P < 0.05, **P < 0.01, ***P < 0.001, ****P < 0.0001. Source data are available online for this figure.

To investigate the behavior of different GSCCs *in vivo*, we generated an orthotopic xenograft model in zebrafish embryos. The eight GSCCs that were used in the co‐culture assay were stably labeled with GFP using viral transduction and injected into *Tg(mpeg1:mCherryF)* ^*ump2*^; *Tg(kdrl:lynEYFP)* ^*md77*^ zebrafish embryos (Table EV2), characterized by mCherry‐expressing macrophages (and microglia) and YFP‐labeled vasculature, at 30 hpf (Ellett *et al*, 2011; Silva *et al*, 2021). Next, live‐imaging of these zebrafish avatars was performed to directly capture dynamic interactions between xenografted GSCCs and GAMs. Zebrafish avatars were typically imaged for 8–16 h at two different timepoints to follow tumor progression: at 1 day post injection (dpi) and again at 5 dpi (Fig 5A). The number of embryos used for the different GSCCs varied due to the challenging technique and suboptimal temperature for embryo development and tumor cell proliferation (Fig EV3A). Based on the morphology of the GAMs, a distinction was made between round and ramified GAMs (Fig 5B). Time‐lapse movies were generated for all GSCCs (Fig 5C–J). We developed an image analysis pipeline for image processing in 3D and over time to compute GSCC‐specific morphometrics and dynamics of the tumor and its microenvironment (Fig 6A).

Although for most GSCCs the tumor tended to decrease in size, we observed a clear difference in the way tumors evolved over time (Fig 5C–J, Movies [Link], [Link]). Some GSCCs (BT333, CME037, LBT001 and LBT070) were more invasive, while other GSCCs (BT569, CME038, LBT003 and LBT123) were more confined. Cell numbers of injected GSCCs typically declined from 1 dpi onwards, with significant reduction to about 30% of the original tumor volume at 5 dpi for all GSCCs, except for LBT001 (Figs 6B and EV3B), suggesting that cancer cell proliferation was slower than clearance by the embryos' immune system. Tumors of BT333, CME037, LBT001, and LBT070 showed a more invasive phenotype with infiltrating cells and shape changes over time (Fig 5C, E, G and I). In general, GSCCs displayed invasive protrusions at 1 dpi, and their number even increased toward 5 dpi. Other tumors like BT569, CME038, and LBT123 preferred to establish compact and round tumor masses, while forming a protrusion along the midline (Fig 5D, F and J, indicated with arrows). Interestingly, LBT003 tumors were significantly larger than most other tumors at 1 dpi, and this was even more pronounced at 5 dpi (Figs 5H and EV3C and D). LBT003 tumors also formed a protrusion along the midline (Fig 5H, arrow). Furthermore, we noticed two different trends in the 1 dpi movies: tumors that decreased in size (BT333, CME037, CME038, LBT003, LBT070, and LBT123), and tumors that slightly grew over time (BT569 and LBT001), while from 5 dpi onwards, tumor size remained roughly unchanged (Fig EV3B).

### Live recordings of dynamic GAM interactions reveal higher infiltration of reactive GAMs in LBT003 avatars

Next, we assessed the interaction between GAMs and engrafted GSCCs over time. We observed two types of GAMs: round GAMs that were compact in shape and located in proximity of the tumor, and ramified GAMs characterized by an elongated cell body and formation of filopodia‐like structures that were located further away from the tumor (Fig 5B and C–J). These morphological phenotypes were described previously and correlate to different functions: round GAMs exert antitumoral properties, while ramified GAMs are more homeostatic and immunosuppressive (McWhorter *et al*, 2013; Heindl *et al*, 2018). Interestingly, our high‐resolution time‐lapse recordings revealed close interaction between round GAMs and GBM tumor cells. Round GAMs appeared to attack and even phagocytize GBM tumor cells, shown by co‐localization of GBM tumor cells and GAMs (Fig EV3E, Movie EV17).

Generally, BT569, CME037, LBT123 and especially LBT003 tumors attracted a lot of round GAMs at 1 dpi (Fig 5D, E, H and J, filled arrowheads), while less GAMs were present around BT333, CME038, LBT001, and LBT070 tumors (Fig 5C, F, G and I, Movies [Link], [Link]). Nevertheless, some of the latter GSCCs attracted some ramified GAMs (Fig 5C, F, G and I, open arrowheads). At 5 dpi, we observed few GAMs overall, which were mostly ramified and did not interact with the GSCCs (Fig 5C–J, open arrowheads). LBT003 avatars were an exception, as round GAMs were present close to the tumor even at 5 dpi (Fig 5H, filled arrowheads).

Quantitative analysis demonstrated that the number of round GAMs decreased for most GSCCs during the time‐lapses starting at 1 dpi, while the number of ramified GAMs remained the same (Fig 6C–F). However, for the paired GSCCs (CME037/CME038) and LBT001, the number of round GAMs slightly increased over time in 1 dpi movies (Fig 6C). Remarkably, at the start of 5 dpi time‐lapses the number of ramified GAMs was significantly higher than at 1 dpi for most GSCCs (Fig 6E and F), while the number of round GAMs was roughly the same at 1 and 5 dpi (Fig 6C and D). This suggests that GAMs switch toward an immunosuppressive phenotype over time.

Next, we calculated the three‐dimensional distance of the GAMs to the tumor, and found that in general the distance of the GAMs significantly increased during the 1 dpi movies (*P* = 6.0e‐7), while this trend was not significant in the 5 dpi movies (*P* = 0.71) (Figs 6G and EV3F and G). Furthermore, for all GSCCs, the round GAMs were located significantly closer to the tumor than the ramified GAMs (*P* < 2.2e‐16) (Fig EV3H), indicating that the round phenotype correlates with reactive and antitumoral properties. Finally, we considered only the GAMs within 30 μm of the tumor, as those are the ones interacting most closely with the tumor. We found that within this distance, GAMs were located closer to LBT003 tumors at 1 and 5 dpi, compared to other tumors, although less pronounced at 5 dpi (Figs 6H and EV3I and J).

Taken together, these observations illustrate that LBT003 avatars have higher infiltration of reactive GAMs compared to other GSCCs.

### Macrophage/GAM—GSCC interactions correlate with clinical outcome in GBM patients

**Figure 7:**
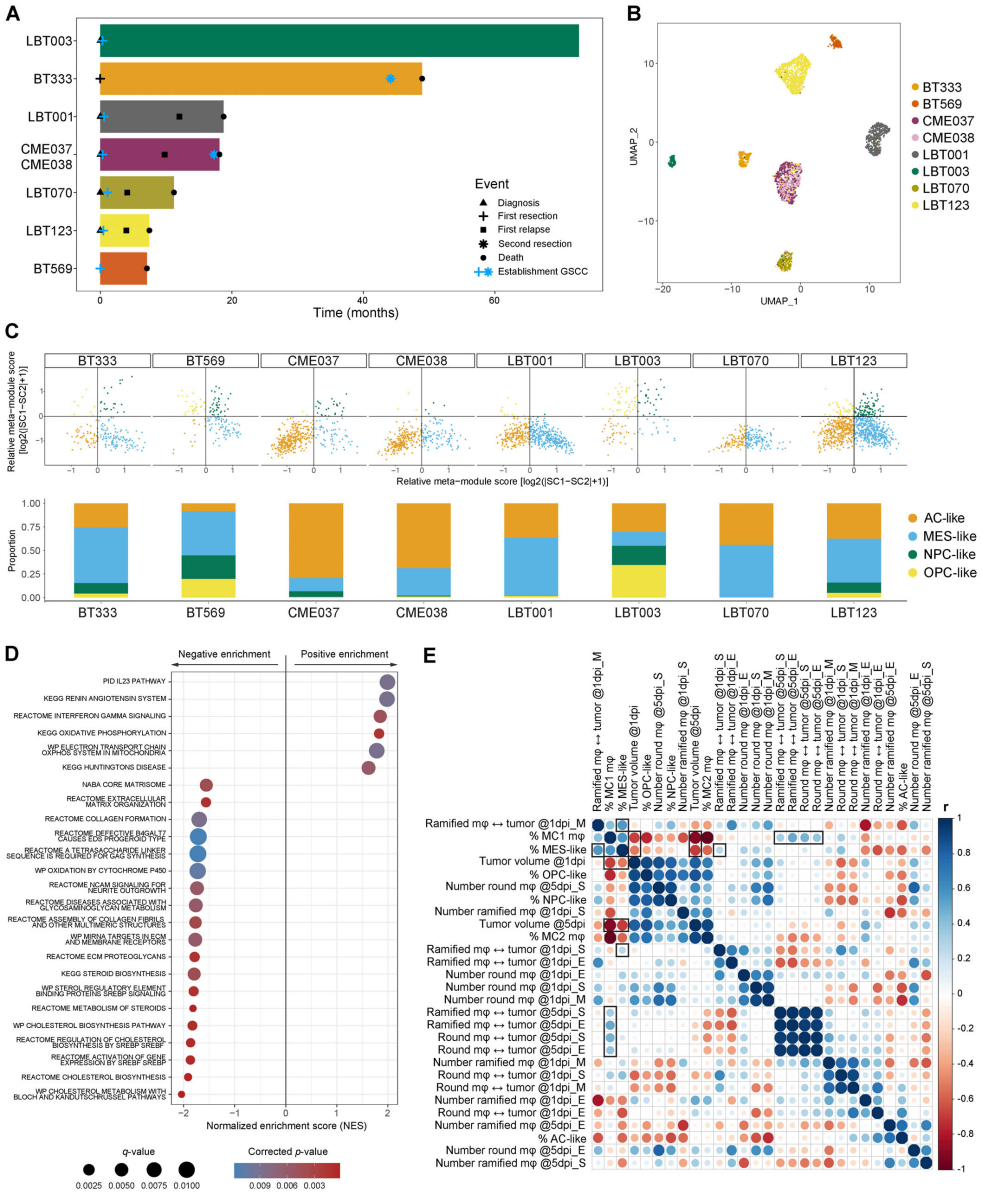
Macrophage/GAM‐GSCC interactions correlate to clinical outcome in GBM patients Swimmer plot of included patients with indication of important events.UMAP plot of GBM tumor cells annotated by sample name.Two‐dimensional butterfly plot visualization of molecular subtype signature scores according to Neftel *et al* (2019) (top). Each quadrant corresponds to a subtype, and the position of each cell reflects its relative signature scores. Colors represent different clusters. Neftel cluster distribution for the different samples (bottom).Dot plot showing the 25 most significant positive and negative enriched GSEA pathways using all curated gene sets of WikiPathways, Reactome, KEGG, PID and BioCarta databases for the group of invasive GSCCs (cut‐off corrected *P*‐value = 0.05).Correlogram of *in vitro* and *in vivo* data of the GSCCs (*n* = 8). Dot size and color indicate the Pearson correlation coefficient (*r*). m*φ*, macrophages/GAMs; ↔, distance to; S, start (24–26 or 114–116 hpi); M, mid (30–32 hpi); E, end (37–39 or 117–119 hpi) indicate timepoints of the time‐lapse movies. Source data are available online for this figure.

Since both the co‐culture model and the zebrafish avatar model identified LBT003 as distinct from the other GSCCs, we were interested in the clinical background of this patient. Interestingly, this patient was diagnosed in the beginning of 2017 with GBM, received SoC treatment, and is still alive today (Fig 7A). With a survival of 76 months (and still counting) this is five times longer than the median survival of a GBM patient receiving SoC treatment (i.e. 15 months) (Grochans *et al*, 2022), and this patient can be considered a “long‐term survivor” (Decraene *et al*, 2023). This suggests that this patient is not only an outlier at the molecular level, as our models have demonstrated, but also in a clinical context. Further research is required to discern whether one is causative of the other.

**Figure EV4:**
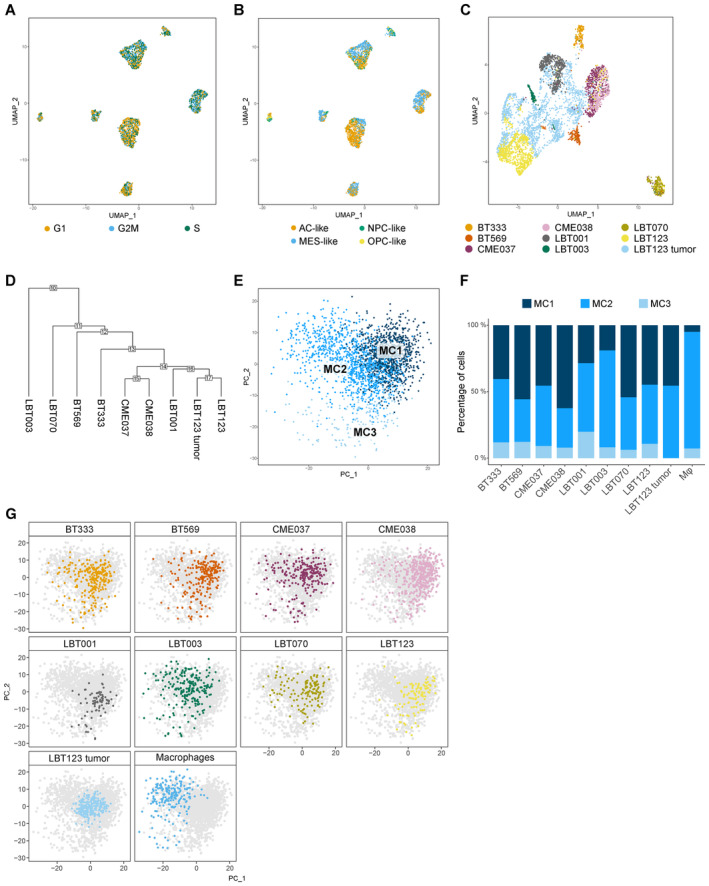
Macrophage/GAM‐GSCC interactions correlate to clinical outcome in GBM patients UMAP plot of GBM tumor cells showing cell cycle score.UMAP plot of GBM tumor cells showing Neftel subtypes.UMAP plot of GBM tumor cells, including cells from the original LBT123 tumor.Clustering tree of GBM tumor cells. The numbers in the boxes at the nodes indicate the order in which the clusters were merged during the hierarchical clustering process. The internal nodes, which start at the number of leaf nodes + 1, represent the nodes that separate groups at different levels of the hierarchical clustering.PCA plot of *CD68* ^+^ cells, including cells from the original LBT123 tumor.Macrophage/GAM subcluster distribution for the different samples, including cells from the original LBT123 tumor. m*φ*, macrophages.Representation of original samples on PCA plot, including the original LBT123 tumor. Source data are available online for this figure.

Next, we performed unsupervised subclustering of the GBM tumor cells, which again revealed patient‐specific clustering (Figs 7B and EV4A, Table EV1). Neftel *et al* (2019) previously reported that GBM tumor cells can be classified into oligodendrocyte‐progenitor (OPC)‐like, neural‐progenitor (NPC)‐like, astrocyte (AC)‐like, or mesenchymal (MES)‐like cell states. Stratifying single GBM tumor cells according to the gene sets described by Neftel *et al* (2019), we found that most GSCCs consisted of a mix of all four subtypes, though at different relative proportions, further illustrating the intra‐ and intertumoral heterogeneity captured within these cell models (Figs 7C and EV4B). Notably, LBT003 contained the smallest proportion of MES‐like cells, which have previously been shown to be more invasive and to be associated with a worse prognosis (Stead, 2022), and is in line with the clinical evolution of LBT003. Interestingly, there was an increase in MES‐like cells in CME038, the recurrent GSCC of CME037, consistent with a recently published study by Wang *et al* (2022a).

Second, we compared the scRNA‐seq data from the original LBT123 tumor (Data ref: Pombo Antunes *et al*, 2021b, sample #ND3) with the scRNA‐seq data from the co‐culture assay to examine how *in vitro* selection affects tumor biology and composition. When integrating the datasets and performing subclustering of the GBM tumor cells, we found that while some tumor heterogeneity was lost during *in vitro* culturing, tumor cells from the original LBT123 tumor clustered closest with the LBT123 GSCC (Fig EV4C), as also evidenced by the clustering tree (Fig EV4D). Interestingly, we found similar macrophage subtypes in the *CD68* ^+^ cells from the original tumor as in the co‐culture assay, except for MC3, the TransMo population that was artificially generated by GM‐CSF stimulation (Fig EV4E–G). Moreover, these cells were most similar to the macrophages co‐cultured with the LBT123 GSCC (Fig EV4G). Taken together, this suggests that our co‐culture model, despite the inherent limitations of *in vitro* models, faithfully reflects the situation of the original tumor.

As we were interested in the overlap of our findings from the scRNA‐seq data and the features extracted from the zebrafish avatar model, we first examined whether the transcriptomic profiles of the GSCCs could explain the differences in invasiveness observed in the avatar model. We previously observed two distinct patterns of tumor evolution in the zebrafish avatar model: invasive (BT333, CME037, LBT001, and LBT070) and non‐invasive (BT569, CME038, LBT003, and LBT123). Therefore, we performed DGE analysis between the invasive and the non‐invasive GSCCs on the scRNA‐seq data from the co‐culture experiments. Gene set enrichment analysis (GSEA) revealed an enrichment for oxidative phosphorylation (OXPHOS) in the invasive GSCCs and an enrichment for extracellular matrix (ECM) production and deposition in the non‐invasive GSCCs (Fig 7D).

Next, we performed a more general correlation analysis to correlate the features extracted from both model systems (Fig 7E). The percentage of immunosuppressive MC1 macrophages was correlated with the distance of GAMs to the tumor at 5 dpi, indicating that immunosuppressive GSCCs were also associated with surveilling GAMs *in vivo*. In addition, the percentage of MC1 macrophages was negatively correlated with the tumor volume at 1 and 5 dpi. Although this may seem contradictory, tumor size is not always related to invasiveness, aggressiveness and thus survival (Wang *et al*, 2019). A large, but well‐aligned tumor, which is the case for LBT003 tumors, improves surgical resection and thus patient's survival, compared to smaller invasive tumors (Mair *et al*, 2018). Finally, the percentage of MES‐like cells was correlated with the distance of ramified GAMs at 1 dpi, and negatively correlated with the tumor volume at 1 and 5 dpi, which is in line with our finding that in LBT003 tumors, which contained the smallest proportion of MES‐like cells, GAMs were located close to the tumor, and LBT003 tumors were bigger than other tumors.

### *LGALS1* is involved in suppression of the immune system

**Figure 8:**
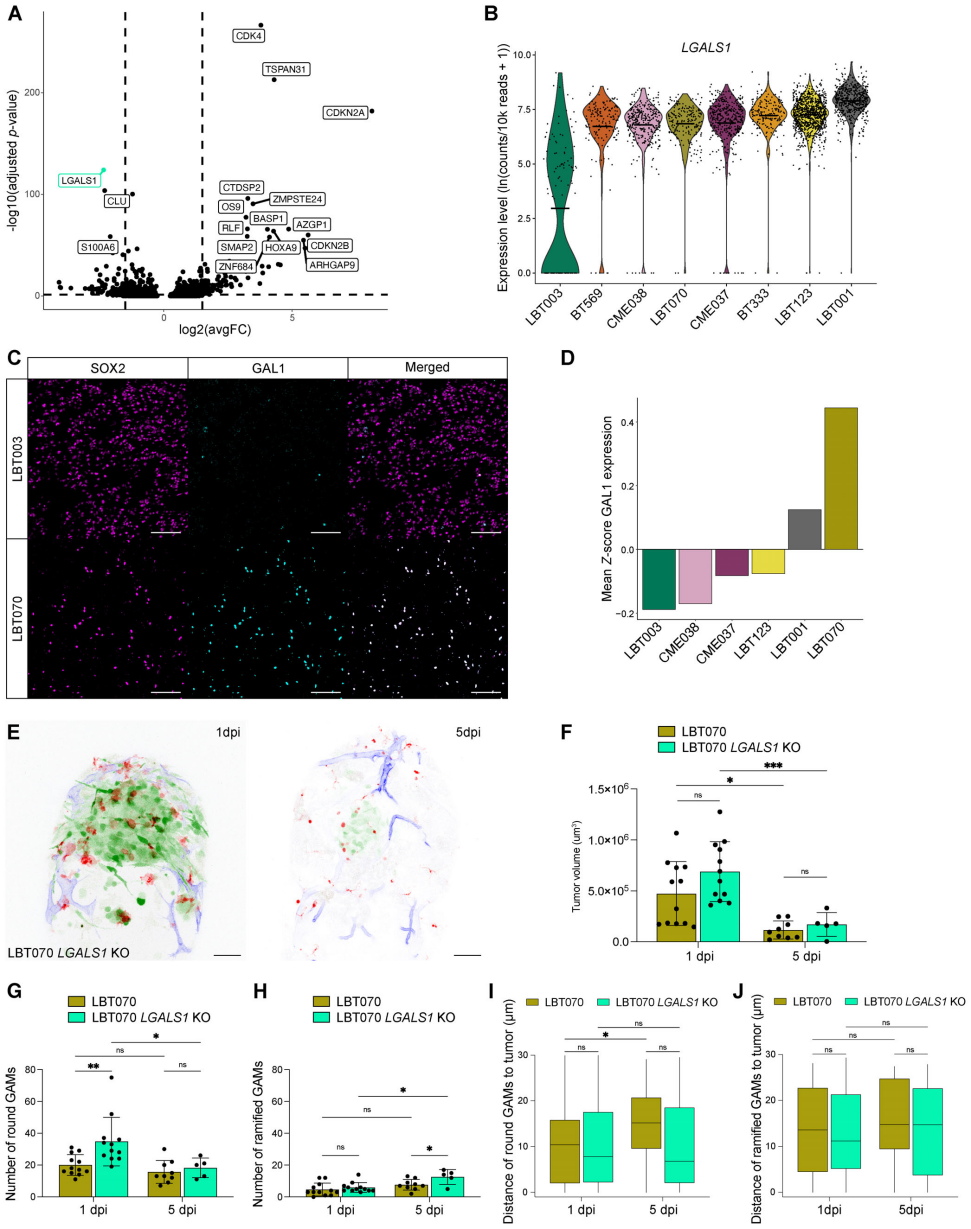
*LGALS1* is involved in suppression of the immune system Volcano plot depicting differentially expressed genes in LBT003 and all other GSCCs (Left: downregulated genes in LBT003; right: upregulated genes in LBT003). *y*‐axis denotes −log_10_(adjusted *P*‐value) while *x*‐axis shows log_2_(avgFC) values. Cut‐offs were set at −log_10_(adjusted *P*‐value) > 1.3 and abs(log_2_(avgFC)) > 1.5.Violin plot showing *LGALS1* expression levels in GSCCs.Representative double immunofluorescence images showing co‐expression of SOX2 (magenta) and GAL1 (cyan) in GBM tissue from LBT003 and LBT070. For enhanced visualization, a binary mask was generated from the SOX2^+^ cells and multiplied with the image of GAL1 staining in Fiji to exclude GAL1 staining in non‐tumor cells. Scale bars: 100 μm.Mean fluorescence intensity values for GAL1 staining in SOX2^+^ cells in GBM tissue samples, normalized using *Z*‐scores within each sample. *n* = 6 tumor samples derived from five different patients.Representative maximum intensity projections of a z stack of the head region of a *Tg(mpeg1:mCherryF)* ^*ump2*^; *Tg(kdrl:lynEYFP)* ^*md77*^ zebrafish embryo with a GFP‐labeled LBT070 *LGALS1* KO tumor, at 1 dpi (left panel) and 5 dpi (right panel). Corresponding control images: see Fig 5I. GBM tumor cells are shown in green, GAMs in red, and blood vessels in blue. Scale bars: 50 μm.Tumor volume at the start of 1 and 5 dpi time‐lapse movies (*n* = 12 and 9 (LBT070; 1 and 5 dpi), 12 and 5 (LBT070 *LGALS1* KO; 1 and 5 dpi) zebrafish embryos; *P* = 0.0118 (LBT070), 0.0008 (LBT070 *LGALS1* KO), 0.0927 (1 dpi), 0.9320 (5 dpi)).Number of round GAMs at the start of 1 and 5 dpi time‐lapse movies (*n* = 12 and 9 (LBT070; 1 and 5 dpi), 12 and 5 (LBT070 *LGALS1* KO; 1 and 5 dpi) zebrafish embryos; *P* = 0.1240 (LBT070), 0.0144 (LBT070 *LGALS1* KO), 0.0053 (1 dpi), 0.1174 (5 dpi)).Number of ramified GAMs at the start of 1 and 5 dpi time‐lapse movies (*n* = 12 and 9 (LBT070; 1 and 5 dpi), 12 and 5 (LBT070 *LGALS1* KO; 1 and 5 dpi) zebrafish embryos; *P* = 0.1816 (LBT070), 0.0187 (LBT070 *LGALS1* KO), 0.6851 (1 dpi), 0.0497 (5 dpi)).Boxplot of GAM distance to the tumor of round GAMs within 30 μm of the tumor, at the start of 1 and 5 dpi time‐lapse movies (*n* = 137 and 48 (LBT070; 1 and 5 dpi), 291 and 39 (LBT070 *LGALS1* KO; 1 and 5 dpi) GAMs; *P* = 0.0119 (LBT070), 0.9984 (LBT070 *LGALS1* KO), 0.9999 (1 dpi), 0.0531 (5 dpi); boxes stand for 50% of the data and minima/maxima are indicated by the line ends).Boxplot of GAM distance to the tumor of ramified GAMs within 30 μm of the tumor, at the start of 1 and 5 dpi time‐lapse movies (*n* = 29 and 13 (LBT070; 1 and 5 dpi), 40 and 20 (LBT070 *LGALS1* KO; 1 and 5 dpi) GAMs; *P* = 0.8012 (LBT070), 0.9647 (LBT070 *LGALS1* KO), 1.0000 (1 dpi), 0.9675 (5 dpi); boxes stand for 50% of the data and minima/maxima are indicated by the line ends). Data information: The zebrafish experiments describe biological replicates, i.e. individual zebrafish embryos. The xenograft experiment was replicated twice for LBT070 *LGALS1* KO, and three times for LBT070. In (F–H), data are presented as mean ± SD, and the *P*‐values were calculated by mixed‐effects model, followed by Šídák's multiple comparisons correction. In (I and J), the *P*‐values were calculated by two‐way ANOVA, followed by pairwise testing with Tukey's multiple comparisons correction. ns ≥ 0.05, *P < 0.05, **P < 0.01, ***P < 0.001. Source data are available online for this figure.

To investigate whether the phenotypic differences between LBT003 and the other GSCCs can be explained by transcriptomic differences, we performed DGE analysis between GBM tumor cells from LBT003 versus the other GSCCs. We found *LGALS1* to be significantly downregulated in LBT003 (Fig 8A and B). Interestingly, *LGALS1* has been shown to be highly expressed in GBM tumor cells and drives therapy resistance (Rorive *et al*, 2001; Chou *et al*, 2018). While GAL1 is also present in the tumor stroma (Chou *et al*, 2018), research has indicated that tumor‐derived, rather than TME‐derived GAL1, is involved in the aggressiveness of glioma progression (Verschuere *et al*, 2014). Moreover, *Lgals1* silencing in GBM reduced macrophages' polarization switch to an immunosuppressive state and was associated with increased survival (Van Woensel *et al*, 2017). The lower level of *LGALS1* expression in LBT003 could thus, at least in part, explain the phenotypic differences between LBT003 and the other GSCCs in the zebrafish avatar model (more GAMs situated closer to the tumor), as well as the general lack of macrophage polarization in the co‐culture model.

**Figure EV5:**
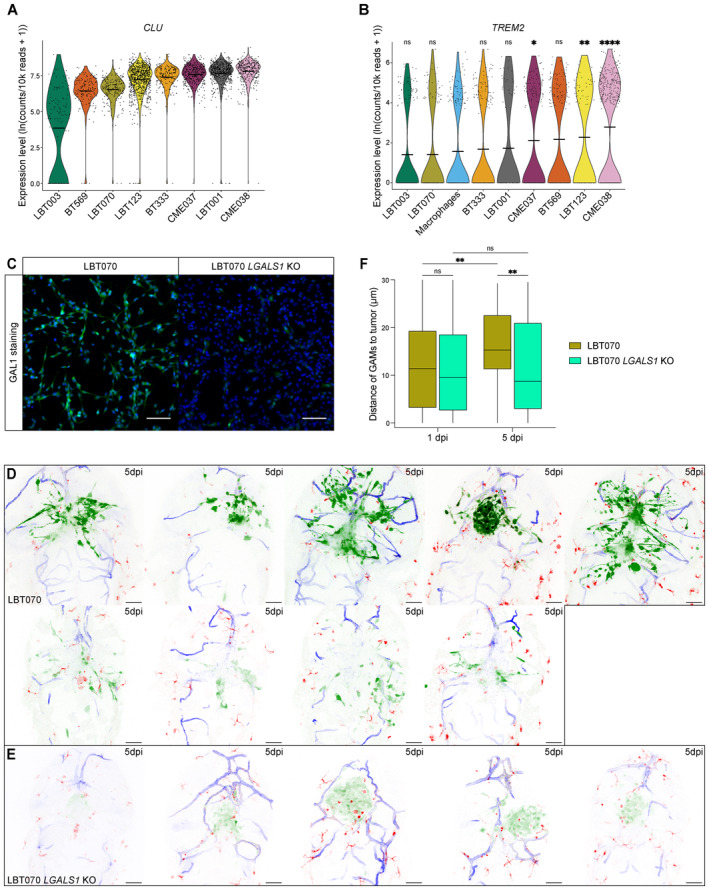
*LGALS1* is involved in suppression of the immune system AViolin plot showing *CLU* expression levels in GSCCs.BViolin plot showing *TREM2* expression levels in macrophages (*P* = 0.8611 (LBT003), 0.5895 (LBT070), 0.3979 (BT333), 0.6995 (LBT001), 0.0122 (CME037), 0.0526 (BT569), 0.0028 (LBT123), 2.8e‐8 (CME038)).CRepresentative immunofluorescence images of LBT070 *LGALS1* WT and LBT070 *LGALS1* KO cells showing expression of GAL1 (green), and cell nuclei stained by DAPI. Scale bars: 100 μm.D, EMaximum intensity projections of a z stack of the head region of *Tg(mpeg1:mCherryF)* ^*ump2*^; *Tg(kdrl:lynEYFP)* ^*md77*^ zebrafish embryos with GFP‐labeled LBT070 (D) and LBT070 *LGALS1* KO (E) tumors at 5 dpi. GBM tumor cells are shown in green, GAMs in red, and blood vessels in blue. Scale bars: 50 μm. *n* = 9 (D) and 5 (E).F Boxplot of GAM distance to the tumor of all GAMs within 30 μm of the tumor, at the start of 1 and 5 dpi time‐lapse movies (*n* = 257 and 99 (LBT070; 1 and 5 dpi), 481 and 83 (LBT070 *LGALS1* KO; 1 and 5 dpi); *P* = 0.0020 (LBT070), 0.9997 (LBT070 *LGALS1* KO), 0.8013 (1 dpi), 0.0067 (5 dpi); boxes stand for 50% of the data and minima/maxima are indicated by the line ends). Data information: The zebrafish experiments describe biological replicates, i.e. individual zebrafish embryos. The xenograft experiment was replicated twice for LBT070 *LGALS1* KO, and three times for LBT070. In (B), the *P*‐values were calculated by Kruskal–Wallis test (*P* = 2.5e‐10). In (F), the *P*‐values were calculated by two‐way ANOVA, followed by pairwise testing with Tukey's multiple comparisons correction. ns ≥ 0.05, *P < 0.05, **P < 0.01, ****P < 0.0001. Source data are available online for this figure.

Similarly, we found that *CLU* is expressed at significantly lower levels in LBT003 compared to the other GSCCs (Figs 8A and EV5A). As previously mentioned, CLU can act as a ligand of the TREM2 receptor on the surface of GAMs, causing polarization toward an immunosuppressive phenotype. Interestingly, we found that *TREM2* expression in macrophages from the LBT003 co‐culture does not significantly differ from the expression in the macrophage monoculture (*P* = 0.8611), while *TREM2* was significantly upregulated in macrophages from the CME037 (*P* = 0.0122), LBT123 (*P* = 0.0028) and CME038 (*P* = 2.8e‐8) co‐cultures (Fig EV5B). The latter two GSCCs were associated with the strongest macrophage polarization toward an immunosuppressive phenotype (see Fig 4D), suggesting that TREM2 signaling may be involved in the polarization process, in addition to *LGALS1*.

To confirm our findings about the top‐hit *LGALS1* at the protein level, we evaluated GAL1 expression in the formalin‐fixed paraffin‐embedded (FFPE) GBM resection specimens from which the GSCCs were derived. Tumor samples from corresponding patients were collected and IHC was used to evaluate the quantity and localization of the GBM tumor cell marker SOX2 and GAL1 (Fig 8C, Table EV2). We found that GAL1 expression in GBM tumor cells was the lowest for LBT003 compared to GBM tumor tissue from other patients (Fig 8C and D).

Next, we knocked out *LGALS1* in LBT070, the GSCC with the highest GAL1 protein level, using CRISPR‐Cas9 technology, and used LBT070 *LGALS1* KO cells in our zebrafish avatar model (Figs 8E, 5I and EV5C, Movies EV18 and EV19). Strikingly, although the reduction in tumor volume by 5 dpi was comparable for LBT070 and LBT070 *LGALS1* KO (both ±75% reduction) (Fig 8F), LBT070 *LGALS1* KO tumors were much more confined at 5 dpi than the invasive LBT070 tumors (Fig EV5D and E). Furthermore, LBT070 *LGALS1* KO tumors attracted significantly more round (*P* = 0.0053), but not ramified GAMs (*P* = 0.6851) at 1 dpi compared to LBT070 tumors (Fig 8G and H). Finally, round GAMs were located equally close to LBT070 and LBT070 *LGALS1* KO tumors at 1 dpi, and remained close to LBT070 *LGALS1* KO tumors at 5 dpi, while moving away from LBT070 tumors (*P* = 0.0119) (Fig 8I), suggesting that *LGALS1* KO prevented the switch to a more immunosuppressive phenotype in the GAMs. We observed a similar trend for all GAMs (Fig EV5F), but not for the ramified GAMs (Fig 8J). Taken together, these results provide direct evidence that *LGALS1* regulates immunosuppression, and this might be correlated with reduced survival in GBM patients.

## Discussion

Novel therapies for GBM are desperately needed to improve prognosis for this fatal disease. GAMs represent a significant proportion (30–50%) of the tumor and often contribute to immunosuppression and tumor progression, making it a valid target for new therapeutic approaches. In addition to treatments targeting tumor cells, novel immunotherapies have been evaluated and several clinical trials on immune checkpoint inhibitors are ongoing. Furthermore, therapeutic approaches for targeting GAMs, including blocking GAM recruitment, GAM reprogramming and facilitating GAM‐mediated phagocytosis are of particular interest. However, these strategies have shown limited efficacy in the clinic so far (Butowski *et al*, 2016). The results obtained in this study, combining scRNA‐seq, zebrafish avatars, and IHC, provide a better understanding of the phenotypic alterations and cellular, molecular, and spatial heterogeneity of GSCCs and associated macrophages. Our findings highlight the need for more precise and context‐specific interventions that selectively target detrimental immunosuppressive GAMs, while sparing beneficial inflammatory cells.

Using scRNA‐seq, we extensively characterized a set of eight diverging GSCCs in an *in vitro* co‐culture model with human monocyte‐derived macrophages. We reported the molecular characteristics of GAMs and detected patient‐specific cell–cell interaction patterns. Even though our findings are based on only eight GSCCs, we uncovered GAM heterogeneity at the molecular level and described a clear phenotype switch, both *in vitro* and *in vivo*. Currently, it is generally accepted that the M1/M2 macrophage terminology originally introduced by Mills *et al* (2000) undermines the complexity of the highly dynamic *in vivo* situation. While the M1/M2 paradigm has been convenient, it has outlived its usefulness in characterizing macrophage subtypes and adds more ambiguity than clarity. With the emergence of single‐cell technologies, various studies identified distinct GAM populations and showed that these highly plastic immune cells express a mix of M1 and M2 markers (Cui *et al*, 2021; Hara *et al*, 2021; Chen *et al*, 2021a; Abdelfattah *et al*, 2022; Karimi *et al*, 2023). Concurrent with these observations, we show that macrophages generally polarize toward a more immunosuppressive phenotype upon co‐culture with GSCCs, though this shift does not clearly follow the M1/M2 classification. Furthermore, we demonstrate that the degree of macrophage polarization in the co‐culture models correlates with GAM recruitment and activity in the corresponding zebrafish avatar models.

We also described the application of advanced live‐imaging techniques in an orthotopic zebrafish xenograft model to continuously monitor the dynamic interplay between transplanted GSCCs and GAMs in real time. In the search for new treatments for GBM, several preclinical models have been developed, including multiple mouse, cell, and organoid models (Zhu *et al*, 2009; Mathivet *et al*, 2017; Ogawa *et al*, 2018; Jacob *et al*, 2020). However, these models do not always accurately reflect the heterogeneity present in the patients, and specifically the complexity of the TME. The use of zebrafish models in cancer research is becoming increasingly popular, due to their unique features like rapid development, small size, transparency of the embryos, ease of genetic manipulation, and ethical and economic advantages (Chen *et al*, 2021b). They have proven highly suited for xenotransplantation of human tumor cells, and GBM tumor cells in particular, due to high similarity with the human brain structure (Reimunde *et al*, 2021). Zebrafish xenograft models are used extensively to study GBM initiation, progression, migration, vasculature and invasion (Vittori *et al*, 2016; Pudelko *et al*, 2018; Pan *et al*, 2020; Umans *et al*, 2021; Mazzolini *et al*, 2022). Hamilton *et al* (2016) studied the interaction between microglia and transplanted GBM cell lines U87 and U251. Concurrent with our results, they observed different growth patterns and microglial responses for both cell lines and confirmed that microglia play a prominent role in promoting GBM tumor cell growth (Hamilton *et al*, 2016). Using an advanced image processing pipeline that was developed in‐house, we were able to capture the complex spatiotemporal interaction between GSCCs and GAMs. Of note, the number of embryos for the different GSCCs was variable due to the challenging technique inherent to the orthotopic zebrafish xenograft model. First, it is difficult to inject the same number of tumor cells. Therefore, tumor size, proliferation, and survival (of both tumor cells and embryos) also vary. Second, zebrafish avatars were grown at 34°C, which is higher than optimal for zebrafish embryo development and lower than optimal for tumor cell proliferation. These aspects should be critically evaluated when designing similar experiments. Nevertheless, time‐lapse movies uncovered differential tumor cell invasion and infiltration of reactive GAMs across the different avatar models. Using GSEA, we also found that non‐invasive GSCCs in the zebrafish avatar model showed an enrichment for ECM production and deposition. This contrasts with the findings of Hoogstrate *et al* (2023), which indicated that an ECM gene signature was associated with significantly worse survival at recurrence, although mainly expressed by pericytes. The enrichment for OXPHOS in invasive GSCCs, on the other hand, is more evident as motile GBM tumor cells have higher energetic needs (Saurty‐Seerunghen *et al*, 2022).

Further, we showed that our models faithfully recapitulated the heterogeneity and clinical features of GBM patients. The rapidly growing field of personalized medicine relies heavily on the use of animal models. Zebrafish avatars of human tumors present a much faster model than murine models and thus hold enormous potential to offer personalized treatment in a clinically relevant time frame. In fact, it has already been demonstrated that zebrafish avatars of various cancer types can predict how individual patients respond to a certain treatment. An example is the study conducted by Fior *et al* (2017), which demonstrated the feasibility of using zebrafish avatars as a tool for testing the efficacy of FOLFOX adjuvant chemotherapy in colorectal cancer patients, as the avatars showed a comparable response to the treatment as their corresponding patients. Although we did not apply therapeutic interventions in this study, our models are ideally suited for pharmaceutical testing and drug discovery. Zebrafish xenograft models have been demonstrated to have clinical features and drug sensitivity similar to human cancers and can thus be used as a fast *in vivo* platform to test existing drugs and to identify relevant novel therapeutic approaches (MacRae & Peterson, 2015; Usai *et al*, 2020; Patton *et al*, 2021). In fact, the zebrafish model has already resulted in the identification of therapeutic targets that were successfully translated to human clinical studies (North *et al*, 2007; Owens *et al*, 2008; Goessling *et al*, 2009; White *et al*, 2011). In the context of GBM, zebrafish xenograft models have become particularly valuable as they allow researchers to assess the ability of drugs to cross the BBB and the presence of systemic toxicity (Zeng *et al*, 2017; Hu *et al*, 2019). Given the substantial heterogeneity and the consistent failure of current therapies in GBM, it is important to explore and develop more individualized GBM treatment strategies. Even though most clinical studies for GBM treatment have failed, there are often exceptional responders who benefit from a particular therapy (Decraene *et al*, 2023). The development and clinical validation of new preclinical models based on patient‐derived samples that allow for a more precise reproduction of the patient's tumor complexity will facilitate a more accurate assessment of whether patients will respond to a specific treatment.

This research identifies galectin‐1 (GAL1, *LGALS1*) as a potential target for immune modulation. We demonstrated that *LGALS1* is involved in immunosuppression, might be correlated with reduced survival in GBM patients, and that *LGALS1* KO in GBM tumor cells can transform the immune landscape. Several research groups have reported similar findings (Baker *et al*, 2014; Barnes *et al*, 2018; Chou *et al*, 2018; Chen *et al*, 2019; Sharanek *et al*, 2021; Guda *et al*, 2022; Zhu *et al*, 2022). *LGALS1* has been shown to drive resistance to chemo‐ and immunotherapy, and to be associated with poor survival. Moreover, chitosan nanoparticles loaded with siRNA targeting *Lgals1* have been developed for intranasal delivery to the tumor and its microenvironment (Van Woensel *et al*, 2016, 2017). Treatment with these nanoparticles reduced immunosuppression and increased survival in tumor‐bearing mice. The authors also described synergistic effects for the combination of anti‐*Lgals1* treatment with chemo‐ and immunotherapy, which indicates that anti‐*LGALS1* nanoparticles could be a valuable adjuvant treatment. In summary, these observations strongly suggest that incorporating *LGALS1*‐targeted therapeutics into the current treatment schedule of GBM patients could be an effective approach for enhancing the outcome for GBM patients.

Furthermore, this study highlights a potential role for TREM2 signaling in GBM disease progression by creating an immunosuppressive environment. Indeed, by predicting L:R interactions between GBM tumor cells and monocyte‐derived macrophages, we revealed that there was more putative TREM2 signaling in co‐cultures that exhibited strong macrophage polarization. TREM2 signaling is known to be involved in many different pathologies, including neurodegenerative disease (Li & Zhang, 2018), obesity (Reich *et al*, 2023), and several cancers (Deczkowska *et al*, 2020; Katzenelenbogen *et al*, 2020), where it plays an important role in immunosuppression. In the context of GBM, one study indicated that *TREM2* expression was correlated with poor tumor immunity and worse prognosis, and that knockdown of *TREM2* resulted in a decrease in M2 polarization (Yu *et al*, 2023). Further studies remain necessary to elucidate detailed mechanisms underlying the immunosuppressive role of both GAL1 and TREM2 signaling.

Taken together, our scRNA‐seq profiling assay complemented with our zebrafish avatar model provide remarkable ability to reveal heterogeneity in GBM tumor growth and interactions within the TME. They can be employed in preclinical research to better mimic GBM and the interactions of GAMs, which could be a crucial step toward improved GBM treatments. Furthermore, we envision our models to act as the foundation for developing a functional screening platform to identify promising immune‐modulating targets. Finally, our fast and sensitive models hold enormous potential to maximize therapy efficacy in a personalized fashion. They could be used to predict a patient's response to certain treatments, as well as to establish better inclusion criteria for clinical trials, resulting in higher success rates.

## Materials and Methods

### Patient‐derived GBM stem cell cultures

Fresh brain tumor tissues were obtained with informed consent from patients undergoing surgical resection. Samples were processed into single‐cell suspensions immediately after resection. The tumor tissue was cut into pieces of 1–2 mm that were mechanically and enzymatically dissociated with the MACS Brain Tumor Dissociation Kit, gentleMACS Dissociator and MACSmix Tube Rotator (Miltenyi Biotec) according to the manufacturer's protocol. The dissociated sample was passed through a 70 μm strainer to remove remaining larger particles. ACK lysing buffer was added to eliminate the red blood cells. After centrifugation, the cell pellet was resuspended in NeuroCult NS‐A Basal Medium (human, STEMCELL Technologies) supplemented with NeuroCult NS‐A Proliferation Supplement (human, STEMCELL Technologies), heparin solution (2 mg/ml, STEMCELL Technologies), recombinant bFGF (20 ng/ml, human, STEMCELL Technologies), recombinant EGF (20 ng/ml, human, STEMCELL Technologies) and antibiotic antimycotic solution (100×; 5 ml, Sigma‐Aldrich), hereafter referred to as complete GBM medium. Live cells were counted using an automated cell counter (Bio‐Rad). Part of the cells were frozen, and the rest was plated in an ultra‐low attachment flask (Corning) and/or a cell culture flask (SARSTEDT) coated with laminin (Sigma‐Aldrich). The medium was changed twice a week. Cells were split when 80–90% confluency was reached. Cell lines were authenticated by STR profiling and tested for mycoplasma contamination. This study and procedures have been evaluated and approved by the Ethical Committee Research of UZ Leuven/KU Leuven (S59804 & S61081). Generated GSCCs were characterized as previously described (Rosenberg *et al*, 2017). BT333 and BT569 were obtained from Dana‐Farber Cancer Institute (Boston, MA, USA) (IRB 10‐417). The experiments conformed to the principles set out in the WMA Declaration of Helsinki and the Department of Health and Human Services Belmont Report.

### Monocyte isolation

Blood was obtained from healthy donors with informed consent and with approval from the Ethical Committee Research of UZ Leuven/KU Leuven (S59804 & S61081) and collected in cell preparation tubes (CPT—Sodium Heparin, BD Biosciences). From the blood, peripheral blood mononuclear cells (PBMCs) were isolated according to the manufacturer's protocol (CPT, BD Biosciences). Next, monocytes were isolated from the PBMCs using a monocyte isolation kit (Pan Monocyte Isolation Kit, human, Miltenyi Biotec). Next, 1 × 10^7^ cells were plated in a T25 cell culture flask and cultured in RPMI 1640 medium with GlutaMAX (Gibco) supplemented with 50 ng/ml GM‐CSF (Recombinant human, R&D Systems), 10% heat‐inactivated FBS (Gibco), and antibiotic‐antimycotic solution (100×, 5 ml), hereafter referred to as complete mφ medium. The rest of the monocytes was frozen in aliquots of 10 × 10^6^ cells. Monocytes were cultured and differentiated into macrophages as previously described (Jin & Kruth, 2016). The experiments conformed to the principles set out in the WMA Declaration of Helsinki and the Department of Health and Human Services Belmont Report.

### Cell culture

All cells were maintained at 37°C and 5% CO_2_. GBM tumor cells and monocytes/macrophages were cultured in complete GBM medium and complete mφ medium, respectively. LBT001, LBT003, LBT070, LBT123, CME037 and CME038 were grown on laminin‐coated flasks. BT333 and BT569 were grown on ultra‐low attachment plates. The medium was changed two to three times a week and cells were passaged when 80–90% confluency was reached. For passaging, GBM tumor cells growing on laminin‐coated plates were collected using a cell scraper, GBM tumor cells growing on ultra‐low attachment plates were collected through centrifugation, and macrophages were harvested using 0.25% trypsin–EDTA (Gibco), after washing with PBS (Gibco) without CaCl_2_ and MgCl_2_. For cell counting, a single‐cell suspension was created by treating the GBM tumor cells for 3 min with accutase (StemPro Accutase Cell Dissociation Reagent, Gibco) at 37°C.

### Co‐culture model of human macrophages and GBM cells

To evaluate the cell–cell interaction between GBM tumor cells and macrophages, co‐culture experiments were carried out as follows: 1 × 10^4^ monocyte‐derived macrophages were seeded together with 4 × 10^4^ GBM tumor cells in hanging drops of 20 μl complete GBM medium (Keller, 1995; Foty, 2011). As a control, a macrophage monoculture was established by seeding 2.5 × 10^4^ macrophages in hanging drops of 20 μl complete GBM medium. The number of droplets per GSCC ranged from 115 to 139. The cells were co‐cultured for 4 days. At the end of the experiment, the cells were labeled with MULTI‐seq for scRNA‐seq.

### MULTI‐seq

MULTI‐seq was used to label cells with sample‐specific barcodes prior to pooling and scRNA‐seq (McGinnis *et al*, 2019). Cells were prepared as follows. Cells were washed with PBS. Macrophages were treated with 0.25% trypsin–EDTA for 10 min at 37°C. Co‐cultures were first treated with accutase for 3 min at 37°C to collect the GBM tumor cells and subsequently with 0.25% trypsin–EDTA for 15 min at 37°C to collect the macrophages. Single‐cell suspensions were then pelleted for 5 min at 300 *g* and washed twice with PBS. Cells were pelleted and resuspended in 200 ml of a 200 nM solution containing equimolar amounts of anchor LMO and sample barcode oligonucleotides in PBS and incubated on ice for 5 min. Next, 20 μl of a 2 μM co‐anchor LMO solution in PBS (for a final concentration of 200 nM) was added to each sample. Following gentle mixing, the labeling reaction was continued on ice for another 5 min. For the remaining procedure, cells were kept on ice. Then, 1 ml of ice cold 1% BSA in PBS was added and cells were pelleted at 4°C. Next, labeled cells were washed twice with ice cold 1% BSA in PBS, counted and pooled into a single aliquot. The pooled sample was concentrated to 10^6^ cells/ml in 1% BSA in PBS. Subsequently, the scRNA‐seq procedure was followed.

### Single‐cell RNA sequencing and analysis

The pooled cell suspension containing several samples individually barcoded by MULTI‐seq was processed with 10× Genomics Technology. The link between the 10× cell barcode and MULTI‐seq barcode was achieved using the deMULTIplex R package (https://github.com/chris‐mcginnis‐ucsf/MULTI‐seq). Cells without viable MULTI‐seq barcode or with ambiguous MULTI‐seq barcode were discarded. Cell‐barcoded 5′ gene expression libraries were sequenced on an Illumina NovaSeq6000 system and mapped to the GRCh38 human reference genome using CellRanger (10× Genomics). Raw gene expression matrices were generated using CellRanger. Downstream analysis was performed with the Seurat R package (Satija *et al*, 2015; Butler *et al*, 2018; Stuart *et al*, 2019; Hao *et al*, 2021). Cells with unambiguous MULTI‐seq barcode, expressing > 200 and < 6,000 genes, containing > 400 unique molecular identifiers (UMIs) and < 20% mitochondrial counts were retained in the analysis. After normalization and regression for the number of UMIs, percentage of mitochondrial genes and cell cycle (S and G2M scores were calculated by the *CellCycleScoring* function in Seurat), the PCs were calculated based on the 2,000 most variable genes. The PCs explaining the highest variance were used for clustering (SNN + Louvain) (Ertöz *et al*, 2003; Blondel *et al*, 2008) and visualization with UMAP (preprint: McInnes *et al*, 2018). Clusters in the resulting two‐dimensional UMAP representation consisted of distinct cell types, which were identified based on the expression of marker genes. Differential expressed genes were identified using the MAST test (Finak *et al*, 2015) with *FindMarkers* and *FindAllMarkers* functions in Seurat. Following manual annotation of GBM tumor cells and macrophages, based on expression of *SOX2* and *CD68*, respectively, subclustering of macrophages only and GBM tumor cells only was performed using the same procedures as described above (2,000 most variable genes, selection of most relevant PCs, UMAP).

Following SNN clustering and Louvain community detection, three distinct clusters were identified in the macrophage dataset. Following marker analysis using the *FindAllMarkers* functions in Seurat, one cluster was identified as TransMos (MC3). For subsequent pseudotime analyses, this cluster was removed, and subclustering was performed again with the remaining two clusters, following the same steps described above. PCA revealed that the bulk of the variance between the two remaining clusters (MC1/MC2) was defined by PC1.

As such, the PC1 value was used as a measure of pseudotime along the MC2‐MC1 axis. To identify temporally expressed genes, a general additive model was fitted for the 1,000 most variable genes with a locally estimated scatterplot smoothing (LOESS) term for the PC1 value, using the tradeSeq R package (*fitGAM* function, *n* = 4 knots) (Van den Berge *et al*, 2020). A heatmap of the 100 most significantly temporally expressed genes (as identified with the *associationTest* function) was generated using the *predictSmooth* function (*n*Points = 50) and the pheatmap R package (https://CRAN.R‐project.org/package=pheatmap).

CellPhoneDB analysis was performed to detect putative L:R interactions between GSCCs and macrophages (and vice versa) (Vento‐Tormo *et al*, 2018; Efremova *et al*, 2020). CellPhoneDB package v4.0.0 (Garcia‐Alonso *et al*, 2022) was used to perform pairwise comparison between GBM tumor cells and macrophages (and vice versa) by randomly permuting labels of the clusters 1,000 times (default setting), thus generating a null‐distribution of each L:R pair, and then determining the actual mean log_2_ expression of the L:R pair. Of note, only receptors and ligands that were detected in at least 10% of the cells in each respective cluster of interest were considered for the analysis (default setting). *P*‐values for each interaction were then determined by comparing the proportion of the means that were equal to or higher than the actual mean of a given L:R pair. Only interactions with a *P*‐value ≦ 0.05 were included in the figures.

Subclustering of GBM tumor cells was performed as described above (regression of cell cycle, selection of 2,000 most variable genes, selection of most relevant PCs, UMAP). Distinct markers for LBT003 were determined using the MAST test (Finak *et al*, 2015) with the *FindMarkers* function in Seurat. Stratification of the single GBM tumor cells into the Neftel subtypes was performed as described by Neftel *et al* (2019).

Single‐cell data integration was performed according to Stuart *et al* (2019). For Fig EV4C–G, a scRNA‐seq dataset containing cells from the primary tumor of patient LBT123 was integrated with the dataset containing the GSCCs from all co‐cultures, based on canonical correlation analysis using the *FindIntegrationAnchors* and *IntegrateData* functions in Seurat, using the 3,000 most variable features. A cluster tree (Fig EV4D) of the integrated dataset, representing the phylogenetic relationship between the different samples, was generated based on a distance matrix constructed in PCA space, using the *BuildClusterTree* function in Seurat.

Gene set enrichment analysis was performed with the *clusterProfiler* (version 3.16) package in R. Canonical pathway gene sets were downloaded from MSigDB (Subramanian *et al*, 2005), which combines gene sets from the WikiPathways, Reactome, KEGG, PID and BioCarta databases. Gene sets with a size < 15 or > 500 genes were excluded from the analysis. A ranked list of log_2_(FC) was used as an input of the *GSEA* function of the *clusterProfiler* package. The 25 most significant pathways (ranked by corrected *P*‐value) were subsequently visualized with the *dotplot* function.

### Transduction (GFP labeling & *LGALS1* KO)

For visualization in the zebrafish avatar model, GBM tumor cells were stably labeled with GFP using lentiviral transduction (PD‐18‐046: pCH‐EF1a‐eGFP‐IRES‐Puro, 1.18 × 10^8^ TU/ml, Leuven Viral Vector Core (LVVC)) (Table EV2). For *LGALS1* KO in GBM tumor cells, CRISPR/Cas9 technology was used. All‐in‐one plasmid was adapted from pXPR023 (Broad Institute, Table EV2) by replacing the puromycin resistance gene with eGFP. Expression of humanized *S. pyogenes* Cas9 was driven by the EFS‐NS promoter in a bicistronic cassette using eGFP as a reporter gene. Four guide RNAs (gRNAs) were designed using the online tool of Broad Institute (https://portals.broadinstitute.org/gpp/public/analysis‐tools/sgrna‐design) to maximize the chances of effective *LGALS1* KO (Table EV2). Single gRNA sequences were integrated in the plasmid under control of the human U6 promoter, yielding four different plasmids. Lentiviral particles were generated from an equimolar mixture of the plasmids by LVVC (PD‐22‐119: pXPR023‐eGFP, 1.03 × 10^7^ TU/ml). Cells with most optimal GFP labeling were grown and sorted using FACS to exclude non‐transduced cells. Cells were sorted with a Sony MA900 cell sorter from the KU Leuven Flow and Mass Cytometry Facility.

### Animal care and handling

*In vivo* experiments were conducted in zebrafish (*Danio rerio*). Adult fish were maintained under standard laboratory conditions (Nüsslein‐Volhard & Dahm, 2002). Zebrafish embryos were raised and staged as previously described (Kimmel *et al*, 1995). All zebrafish were maintained and handled in compliance with European Animal Welfare Legislation (2010/63/EU) and FELASA guidelines and recommendations concerning laboratory animal welfare and scientific use. All protocols involving work with live animals that are described below were reviewed and approved by the Animal Ethics Committee of the KU Leuven (P167/2021). The experiments of this project were performed by LF, who holds a FELASA B certificate, in the zebrafish facility and laboratory at VIB – KU Leuven.

### Transgenic and mutant zebrafish lines

*Tg(mpeg1:mCherryF)* ^*ump2*^ zebrafish embryos were used for the visualization of macrophages (and microglia) (Table EV2) (Ellett *et al*, 2011; Silva *et al*, 2021). The vascular reporter line *Tg(kdrl:lynEYFP)* ^*md77*^ was generated in the mutant transparent *casper* background for improved *in vivo* visualization (White *et al*, 2008). Growing and breeding of transgenic and mutant lines was done in accordance with the regulations of the Animal Ethics Committee of the KU Leuven.

### Zebrafish transgenesis

The construct for transgenesis of the *Tg(kdrl:lynEYFP)* ^*md77*^ line (Table EV2) was generated by replacing dTomato with the coding sequence of lynEYFP in the pTol2‐kdrl:dTomato plasmid, using the adjacent restriction sites present in the vector (Table EV2) (Jakobsson *et al*, 2010). Transgenesis was done via Tol2‐mediated recombination as previously described (Kawakami, 2004; Kwan *et al*, 2007). *Casper* embryos were co‐injected at one‐cell stage with 100 pg of Tol2 mRNA and 40 pg of pTol2‐kdrl:lynEYFP plasmid DNA. Embryos were raised at 28°C and screened for EYFP expression at ~72 hpf.

### Orthotopic zebrafish xenograft model

A clutch of eggs was produced by crossing multiple male and female zebrafish, making each embryo an independent biological replicate and mitigating batch effects. Heterozygous mutant *casper* embryos were treated with 0.003% 1‐phenyl‐2‐thiourea (PTU, Sigma‐Aldrich) from 24 hpf onwards to delay pigmentation. At 30 hpf, anesthetized (0.014% tricaine, MS‐222, Sigma‐Aldrich) *Tg(mpeg1:mCherryF)* ^*ump2*^; *Tg(kdrl:lynEYFP)* ^*md77*^ embryos were randomly selected from the batch to be microinjected with GFP‐expressing GBM tumor cells into the hindbrain ventricle. One GSCC was used per batch of embryos. Approximately 300–800 cells were injected per embryo and zebrafish avatars were grown at 34°C until the end of the experiment. At 1 dpi (54 hpf), zebrafish avatars were screened for the presence of GBM tumor cells and those with the largest tumors were selected for overnight imaging. At 5 dpi (144 hpf), the same avatars were imaged again to follow tumor progression.

ARRIVE guidelines were followed for zebrafish experiments. Due to the observational nature of our study and the lack of a pilot study or preliminary results, the expected effect size and standard deviation in the population were unknown. In addition, the dropout rate in the technically challenging zebrafish xenograft model was unpredictable. Therefore, sample size could not be calculated. Measurement order and other confounders were not controlled. However, time‐lapse imaging eliminates the potential effect of measurement order. No blinding was performed when conducting the experiments.

### Live‐imaging

Embryos were anesthetized in 0.014% tricaine, mounted in a 35 mm glass bottom petri dish (0.17 mm, MatTek) using 0.6% low‐melting‐point agarose (Lonza) containing 0.014% tricaine, and bathed in Danieau buffer containing 0.007% tricaine and 0.003% PTU. Time‐lapse imaging was performed using a Leica TCS SP8 upright microscope with a Leica HCX IRAPO L ×25/0.95 water‐dipping objective and heating chamber. Zebrafish avatars were typically imaged at 34°C at two different timepoints: 1 and 5 dpi. Time‐lapse images were acquired for up to 16 h at 1 dpi and up to 8 h at 5 dpi and time intervals ranged from 15 to 30 min.

### Image processing

The 3D time‐lapse movies were processed by image analysis and machine learning algorithms to faithfully segment tumor and GAMs over time and classify GSCC‐specific morphometrics and dynamics of the tumor and its microenvironment. Our dataset contains 190 3D time‐lapse movies consisting of three channels: GBM tumor cells, GAMs, and vasculature. If a zebrafish embryo moved out of focus, that timepoint and subsequent timepoints were removed from the dataset. Image pre‐processing was performed using Fiji (version 2.0.0) (Schindelin *et al*, 2012), including conversion to tiff image file format and separation of the channels. Bleed‐through of the tumor signal into the vascular channel was removed. Autofluorescence correction proved necessary especially for 5 dpi movies due to increased pigmentation of the zebrafish embryos and was achieved using ilastik's pixel classification algorithm (Berg *et al*, 2019), which was trained on annotations of tumor, GAMs, autofluorescence and background. After obtaining the classification, the pixels containing autofluorescence were extracted and removed. Following autofluorescence removal, the tumor and GAM channels were analyzed using a customized image analysis pipeline. The tumor channel was denoised using a Gaussian blur filter and segmented by Otsu thresholding (Otsu, 1979). For GAM segmentation, the GAM channel was initially max‐intensity projected in 2D. The 2D GAM images were segmented using the deep learning algorithm Cellpose (Pachitariu & Stringer, 2022). The Cellpose model was trained using more than 1,000 manually annotated GAMs on 42 2D images, equally distributed over movies starting at 1 and 5 dpi, and all GSCCs. Subsequently, the Cellpose model was applied to all movies to obtain a segmentation of the single GAMs. GAM segmentation was validated in a subset of movies by comparing Cellpose‐segmented GAM masks with manually annotated GAM masks. The quality of the segmentation was assessed by identifying correctly segmented GAMs versus false positives and false negatives, based on optimizing the intersection over union (IoU) metric. Subsequently, a 3D from 2D inference for the center positions of the GAMs was applied to identify 3D positions based on the GAM segmentations in 2D. This workflow is an Album solution (preprint: Albrecht *et al*, 2021) accessible on “Album solution”. To compute distances of the GAMs to the tumor, a Euclidean distance transform was applied on the 3D tumor masks. This allowed us to obtain the shortest distance of each GAM center position to the surface of the tumor. For GAMs in contact with or inside the tumor the distance is 0. Finally, automated quantitative analysis was performed whereby various metrics were obtained from the segmentation masks (tumor volume, GAM number, GAM shape, etc.) by using scikit‐image, a collection of image processing algorithms in Python (van der Walt *et al*, 2014). GAM circularity was computed as follows: 4*πA*/*p* ^2^, where *A* is the area of the GAM and *p* its perimeter. A circularity of 1 indicates a perfect circle, while circularity values close to 0 indicate highly non‐circular shapes. Round GAMs were defined as GAMs with a circularity larger than 0.6, while ramified GAMs were defined as cells with a circularity smaller than 0.35. The analysis scripts implemented in Python are available in a GitHub repository. For plotting time courses of different features, an R Shiny app was used, which was derived from PlotTwist (Goedhart, 2020) and can be found in the same repository.

### Immunohistochemistry

FFPE GBM tissue samples (collected at the UZ/KU Leuven biobank according to protocols S59804 and S61081) were used for IHC. Patient material was available for all patients, except for BT333 and BT569. If sufficient material was available, multiple regions were selected per tumor sample, and a tissue microarray (TMA) was created. Our TMA included 15 cores in total, originating from four patients (LBT001, LBT003, CME037 and CME038). For LBT070 and LBT123, whole‐slide (WS) sections were used due to limited tissue availability.

First, FFPE tissue slides were deparaffinized by sequentially placing them in xylene, 100% ethanol and 70% ethanol. Following dewaxing, antigen retrieval was performed with PT link (Agilent) using 10 mM EDTA in Tris‐buffer (pH 8). Next, slides were placed in bleaching buffer to lower the intrinsic autofluorescent background signal. Immunofluorescence staining was performed using Bond RX Fully Automated Research Stainer (Leica Biosystems) with anti‐GAL1 (Cell Signaling Technology, 13888, 1:375) and anti‐SOX2 (ThermoFisher Scientific, 14‐9811‐82, 1:150) antibodies (Table EV2). Tissue sections were incubated for 4 h with the previously validated primary antibodies, washed, and then incubated with fluorescently labeled secondary antibodies for 30 min. A coverslip was placed onto the slides with medium containing DAPI to stain cell nuclei. A high‐resolution image was generated at ×10 magnification using a Axio Scan.Z1 slide scanner (Zeiss).

Raw scans were transformed to grayscale 16‐bit tiff images using the developer's software (Zen). Image registration was performed by applying a homomorphic transformation over a set of matched descriptors using a Harris detector. Images were subsequently adjusted for background intensity variations using the rolling ball algorithm, and autofluorescence removal was performed by subtracting the pre‐stained image of the corresponding tissue section from the measured signal. Intensities for both images were normalized using quantile normalization. For cellular segmentation, first, a mask was generated from the DAPI channel of the pre‐stained image using a local thresholding approach. Second, a distance map was computed using the binary image and subsequently the distance map was divided into cellular objects using the watershed algorithm. DAPI‐positive objects were expanded by 5 pixels to capture the expression of markers present in the cell cytoplasm. For each cellular object, morphological (nuclear size) and functional (marker intensity) features were extracted. Since the entire cell surface does not express all markers, the expression of each cell was summarized by the 95% quantile of the expression of all its pixels. Mean fluorescence intensity values for GAL1 staining in SOX2^+^ cells were normalized using *Z*‐scores within each core/WS and averaged per patient, as previously described (Caicedo *et al*, 2017). To avoid a strong influence from outliers, *Z*‐scores were trimmed within the [−5, 5] range. For enhanced visualization, a binary mask was generated of the SOX2^+^ cells and multiplied with the image of GAL1 staining in Fiji (version 2.0.0) to exclude GAL1 staining in non‐tumor cells (see Fig 8C).

LBT070 cells and LBT070 *LGALS1* KO cells were stained with anti‐GAL1 antibody (Cell Signaling Technology, 13888, 1:375) and staining was evaluated using the Operetta High Content Imaging System (PerkinElmer) (See Fig EV5C).

### Statistics

Statistical details of all analyses are reported in the figure legends. All statistical tests used were two‐sided unless otherwise mentioned. All reported measurements were taken from distinct samples and not repeatedly measured on the same sample, unless at different timepoints (1 and 5 dpi). For Figs 6B, D and F and 8F–H, two‐way repeated measures ANOVA (or mixed‐effects model in case of missing values) was used, for which no non‐parametric equivalent exists. For all other statistical analyses of *in vivo* data, normality was either confirmed by Shapiro–Wilk and/or Kolmogorov–Smirnov normality tests or assumed according to the central limit theorem (*n* > 30). If the data were not normally distributed, a non‐parametric test was used. Subsequently, the Brown‐Forsythe test was used to test whether the variances of the statistically compared groups were equal. If the variances were similar, an appropriate parametric test was used, if the variances were significantly different, a non‐parametric alternative was used. All statistical analyses or graphical representations were executed using GraphPad Prism version 9.5.1, or R Studio version 2022.12.0+353.

## Author contributions

**Lise Finotto:** Conceptualization; data curation; formal analysis; funding acquisition; validation; investigation; visualization; methodology; writing – original draft; project administration; writing – review and editing. **Basiel Cole:** Data curation; formal analysis; validation; visualization; writing – review and editing. **Wolfgang Giese:** Data curation; software; formal analysis; validation; methodology. **Elisabeth Baumann:** Data curation; software; formal analysis; validation; methodology. **Annelies Claeys:** Validation; investigation. **Maxime Vanmechelen:** Investigation. **Brecht Decraene:** Investigation. **Marleen Derweduwe:** Investigation. **Nikolina Dubroja Lakic:** Investigation. **Gautam Shankar:** Software; formal analysis. **Madhu Nagathihalli Kantharaju:** Software. **Jan Philipp Albrecht:** Software. **Ilse Geudens:** Conceptualization; investigation. **Fabio Stanchi:** Investigation. **Keith L Ligon:** Resources. **Bram Boeckx:** Resources. **Diether Lambrechts:** Resources. **Kyle Harrington:** Software; methodology. **Ludo Van Den Bosch:** Resources. **Steven De Vleeschouwer:** Resources. **Frederik De Smet:** Conceptualization; supervision; funding acquisition; methodology; project administration; writing – review and editing. **Holger Gerhardt:** Conceptualization; supervision; funding acquisition; methodology; project administration; writing – review and editing.

## Disclosure and competing interests statement

SDV is patent co‐holder of GB1519841.9 (filed as international application N° PCT/EP2016/078547), initiated by KU Leuven (LRD) and ULB (TTO) in mutual agreement: “Treatment of central nervous tumors” (Patent N° WO2017089392‐A1 CA3005992‐A1 AU2016358718‐A1). The patent relates to intranasal delivery of nanoparticles encapsulating siRNA for gene silencing of galectin‐1 as treatment of nervous brain tumors, such as GBM. The other authors declare that they have no competing interests or disclosures.

## Supporting information



Expanded View Figures PDF

Table EV1

Table EV2

Movie EV1

Movie EV2

Movie EV3

Movie EV4

Movie EV5

Movie EV6

Movie EV7

Movie EV8

Movie EV9

Movie EV10

Movie EV11

Movie EV12

Movie EV13

Movie EV14

Movie EV15

Movie EV16

Movie EV17

Movie EV18

Movie EV19

Source Data for Expanded View

PDF+

Source Data for Figure 2

Source Data for Figure 3

Source Data for Figure 4

Source Data for Figure 6

Source Data for Figure 7

Source Data for Figure 8

## Data Availability

The plasmid used to generate the *Tg(kdrl:lynEYFP)* ^*md77*^ zebrafish line is available via European Zebrafish Resource Center (EZRC) (ZFIN ID: ZDB‐ALT‐230627‐2, Table EV2). The datasets and computer code produced in this study are available in the following databases: (i) Oncoprint data: European Genome‐phenome Archive EGAS00001007481 (https://ega‐archive.org/studies/EGAS00001007481), (ii) scRNA‐seq data: European Genome‐phenome Archive EGAS00001007482 (https://ega‐archive.org/studies/EGAS00001007482), (iii) Imaging dataset: BioImage Archive S‐BIAD770 (https://www.ebi.ac.uk/biostudies/bioimages/studies/S‐BIAD770), (iv) Image analysis pipeline (computer scripts): GitHub (https://github.com/wgiese/zebrafish_xenograft_analysis).
