# Supplementary material for: Single‐cell profiling and zebrafish avatars reveal LGALS1 as immunomodulating target in glioblastoma
Source: EMBO Mol Med. 2023 Oct 4;15(11):e18144. doi: 10.15252/emmm.202318144 (PMC10630887; doi:10.15252/emmm.202318144)
Supplement: Supplementary file 1 — Expanded View Figures PDF [file EMMM-15-e18144-s003.pdf]

# Expanded View Figures

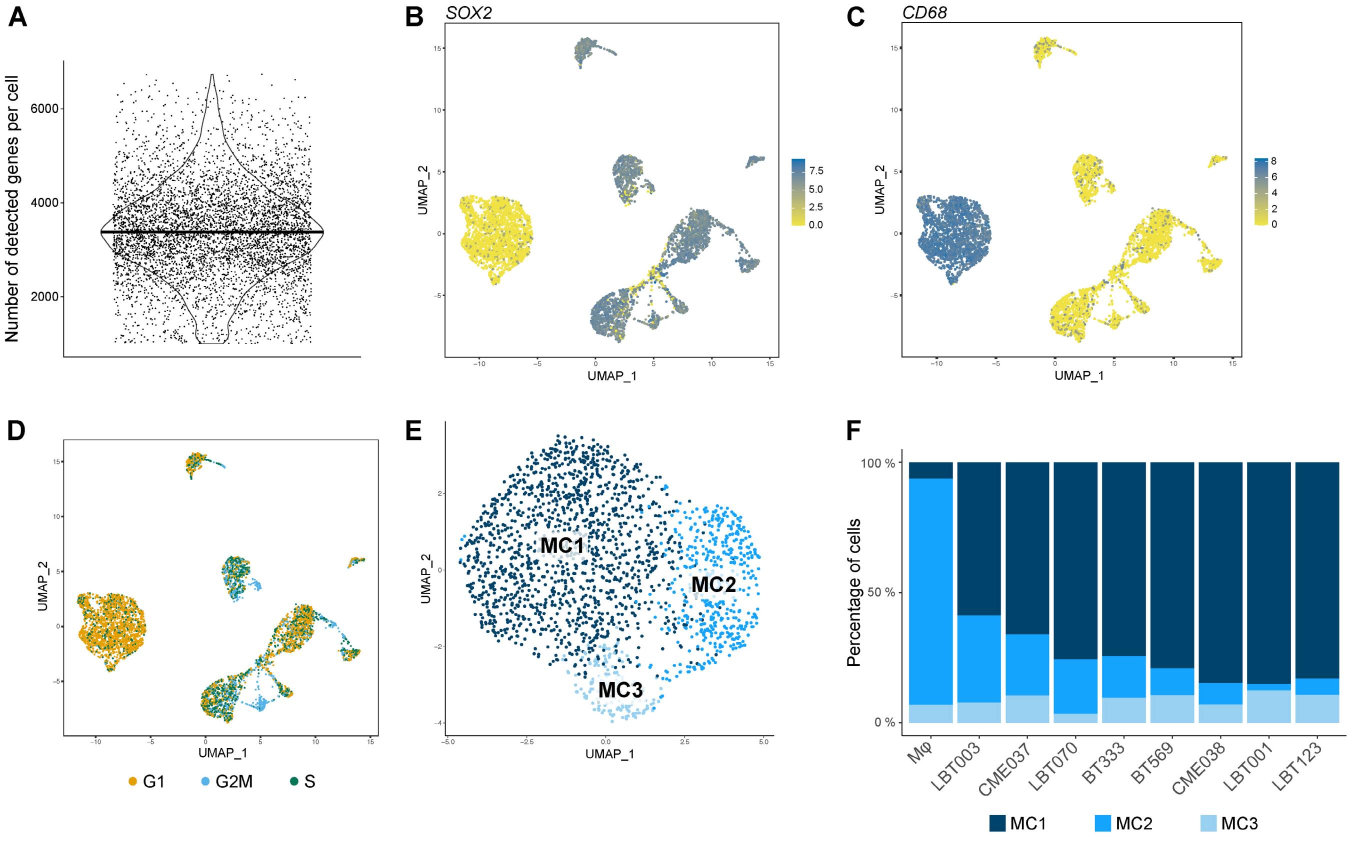


**Figure EV1. Single-cell profiling of GSCC-macrophage co-cultures reveals molecular heterogeneity of GBM-associated macrophages.**

A Violin plot showing the number of detected genes per cell ($n$ = 5,320 cells from nine samples with a median of 3,334 genes detected per cell).
B, C UMAP plots showing *SOX2* (B) and *CD68* (C) expression.
D UMAP plot showing cell cycle score. Proliferating GBM tumor cells are depicted in blue.
E UMAP plot of macrophage population identified three distinct macrophage subclusters (MC1-3).
F Macrophage subcluster distribution for the different samples. m$\varphi$, macrophages.

Source data are available online for this figure.

**Figure EV2. Macrophages shift toward an immunosuppressive phenotype upon co-culture with patient-derived GSCCs.**

A UMAP plot of macrophage population without TransMos shows two distinct macrophage subclusters (MC1-2).
B Representation of original samples on the UMAP plot.
C–G LOESS plots for *AKR1B1* (C), *CCL4* (D), *MSR1* (E), *LIPA* (F), and *LGALS1* (G).
H Dot plot of cell–cell communication analysis using CellPhoneDB. Depicted are L:R pairs for macrophage - GSCC signaling across all GSCCs, ranked by mean $\log_2$ expression. Each dot size shows the $\log_2$ mean of expression values and dot color indicates the *P*-value for the listed L:R pairs (*x*-axis) in the respective GSCCs (*y*-axis). Only top 50 significant L:R pairs, with cut-offs of *P*-value ≦ 0.05 are shown. The *P*-values were generated by CellPhoneDB, which uses a one-sided permutation test to compute significant interactions.

Source data are available online for this figure.

© 2023 The Authors

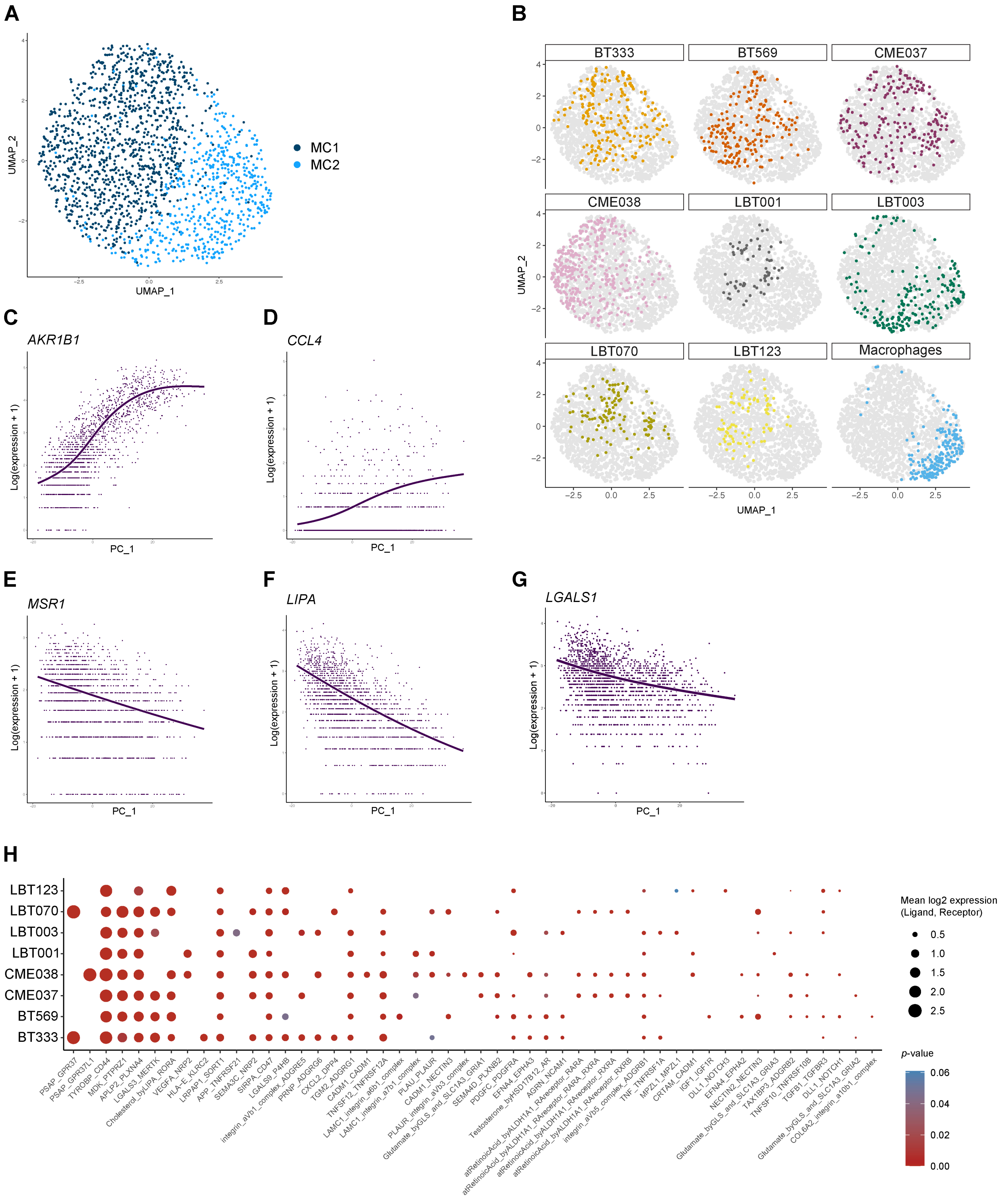


**Figure EV2.**

 © 2023 The Authors

**Figure EV3. GSCC-specific morphometrics and dynamics of the tumor and its microenvironment in 3D over time.**

A Number of zebrafish embryos used for the time-lapse movies that were generated at 1 and 5 dpi.

B Mean tumor volume over time, during 1 dpi movies (left) and 5 dpi movies (right) ($n$ = 10 (BT333), 12 and 11 (BT569; 1 and 5 dpi), 10 and 9 (CME037; 1 and 5 dpi), 6 (CME038), 8 and 4 (LBT001; 1 and 5 dpi), 22 and 18 (LBT003; 1 and 5 dpi), 12 and 9 (LBT070; 1 and 5 dpi), 15 and 11 (LBT123; 1 and 5 dpi) zebrafish embryos, see also panel A).

C Tumor volume at the start of 1 dpi time-lapse movies ($n$ = 10 (BT333), 12 (BT569), 10 (CME037), 6 (CME038), 8 (LBT001), 22 (LBT003), 12 (LBT070), 15 (LBT123); $P$ = < 1.0e-15 (BT333), 0.0001 (BT569), 0.0017 (CME037), 3.1e-5 (CME038), < 1.0e-15 (LBT001), 0.0002 (LBT070), 0.1542 (LBT123)).

D Tumor volume at the start of 5 dpi time-lapse movies ($n$ = 10 (BT333), 11 (BT569), 9 (CME037), 6 (CME038), 4 (LBT001), 18 (LBT003), 9 (LBT070), 11 (LBT123); $P$ = 0.0009 (BT333), 0.0179 (BT569), 0.0237 (CME037), 0.0095 (CME038), 0.0538 (LBT001), 4.7e-5 (LBT070), 0.0003 (LBT123)).

E Representative maximum intensity projections of a z stack of the head region of a *Tg(mpeg1:mCherryF)*$^{ump2}$; *Tg(kdrl:lynEYFP)*$^{md77}$ zebrafish embryo with a GFP-labeled LBT070 tumor, at 37.5, 38, 38.5 and 39 hpi to illustrate phagocytosis of a GBM tumor cell by a round GAM (indicated by arrows). GBM tumor cells are shown in green, GAMs in red, and blood vessels in blue. Scale bars: 50 μm.

F, G Trend line of median GAM distance to the tumor over time in 1 dpi (F) and 5 dpi (G) movies for all GSCCs ($n$ = 43,857 (F) and 16,639 (G) GAMs; $P$ = 6.0e-7 (F) and 0.7071 (G)).

H Boxplot of distance of round and ramified GAMs to the tumor for all GSCCs, at the start of 1 and 5 dpi time-lapse movies ($n$ = 1,821 and 1,429 (round; 1 and 5 dpi), 420 and 1,008 (ramified; 1 and 5 dpi) GAMs; $P$ < 6.4e-7 (1 dpi) and < 2.4e-5 (5 dpi); boxes stand for 50% of the data and minima/maxima are indicated by the line ends).

I Boxplot of GAM distance to the tumor of round GAMs within 30 μm of the tumor, at the start of 1 and 5 dpi time-lapse movies, ranked by increasing median distance at 1 dpi ($n$ = 325 and 146 (LBT003; 1 and 5 dpi), 273 and 44 (LBT123; 1 and 5 dpi), 134 and 105 (BT333; 1 and 5 dpi), 141 and 49 (BT569; 1 and 5 dpi); 137 and 48 (LBT070; 1 and 5 dpi), 74 and 56 (CME037; 1 and 5 dpi), 26 and 26 (CME038; 1 and 5 dpi), 61 and 11 (LBT001; 1 and 5 dpi); boxes stand for 50% of the data and minima/maxima are indicated by the line ends).

J Boxplot of GAM distance to the tumor of ramified GAMs within 30 μm of the tumor, at the start of 1 and 5 dpi time-lapse movies, ranked by increasing median distance at 1 dpi ($n$ = 63 and 51 (LBT003; 1 and 5 dpi), 26 and 26 (LBT123; 1 and 5 dpi), 38 and 21 (BT569; 1 and 5 dpi), 13 and 12 (CME038; 1 and 5 dpi); 29 and 13 (LBT070; 1 and 5 dpi), 25 and 86 (BT333; 1 and 5 dpi), 18 and 30 (CME037; 1 and 5 dpi), 15 and 3 (LBT001; 1 and 5 dpi); boxes stand for 50% of the data and minima/maxima are indicated by the line ends).

Data information: Data describe biological replicates, i.e. individual zebrafish embryos. The xenograft experiment was replicated once for CME038, twice for BT333, BT569, CME037, LBT001, three times for LBT070 and LBT123, and four times for LBT003. In (C and D), data are presented as mean ± SD. In (C), the $P$-values were calculated by Welch's ANOVA test, followed by Dunnett's T3 multiple comparisons correction. In (D), the $P$-values were calculated by Kruskal–Wallis test, followed by Dunn's multiple comparisons correction. In (F and G), the $P$-values were calculated by the Mann–Kendall trend test. In (H), the $P$-values were calculated by Welch's ANOVA test, followed by Games-Howell's multiple comparisons correction. ns ≥ 0.05, *$P$ < 0.05, **$P$ < 0.01, ***$P$ < 0.001, ****$P$ < 0.0001.
Source data are available online for this figure.

© 2023 The Authors

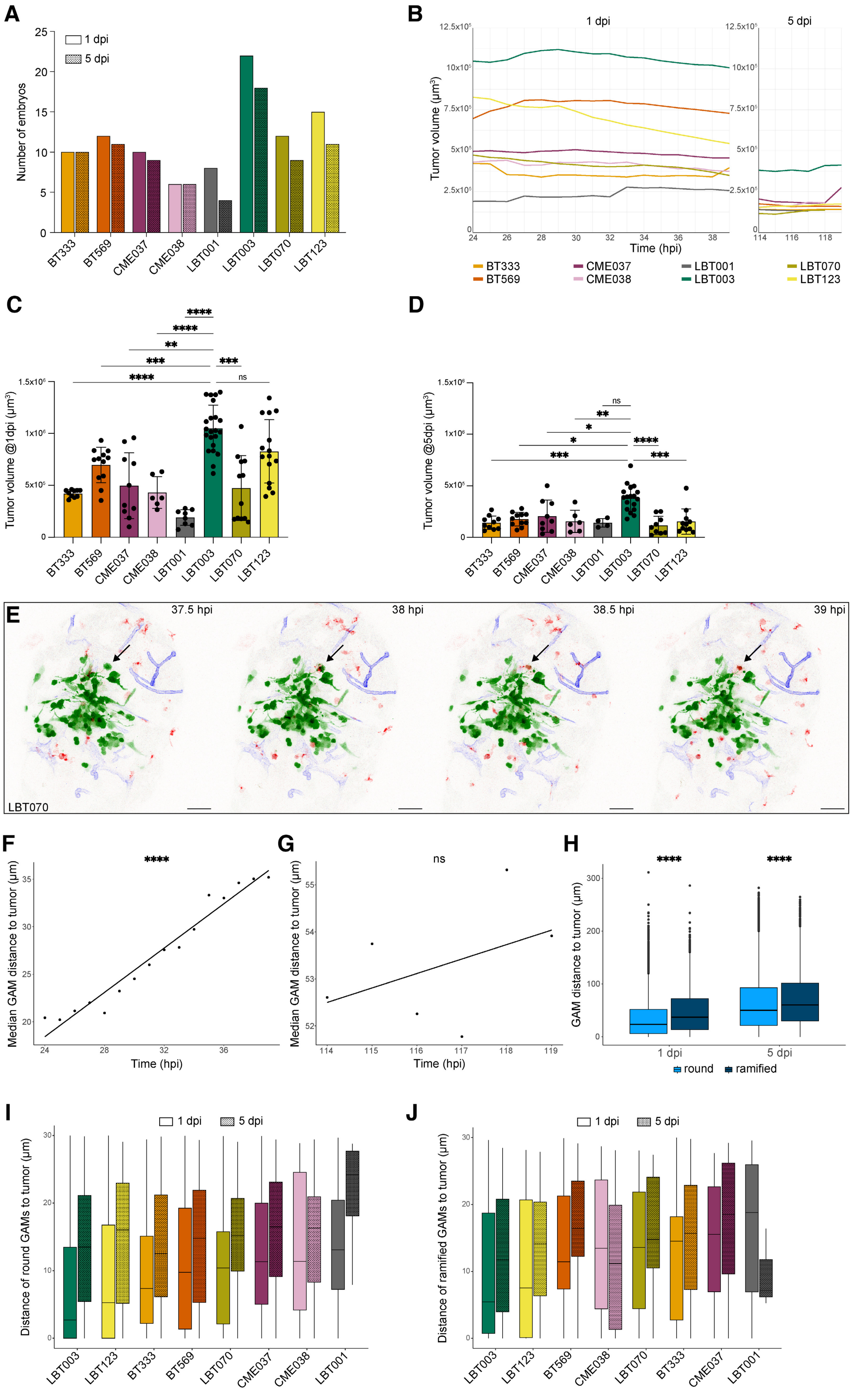


**Figure EV3.**

 © 2023 The Authors

**Figure EV4. Macrophage/GAM-GSCC interactions correlate to clinical outcome in GBM patients.**

A UMAP plot of GBM tumor cells showing cell cycle score.

B UMAP plot of GBM tumor cells showing Neftel subtypes.

C UMAP plot of GBM tumor cells, including cells from the original LBT123 tumor.

D Clustering tree of GBM tumor cells. The numbers in the boxes at the nodes indicate the order in which the clusters were merged during the hierarchical clustering process. The internal nodes, which start at the number of leaf nodes + 1, represent the nodes that separate groups at different levels of the hierarchical clustering.

E PCA plot of *CD68*$^{+}$ cells, including cells from the original LBT123 tumor.

F Macrophage/GAM subcluster distribution for the different samples, including cells from the original LBT123 tumor. m$\varphi$, macrophages.

G Representation of original samples on PCA plot, including the original LBT123 tumor.

Source data are available online for this figure.

© 2023 The Authors

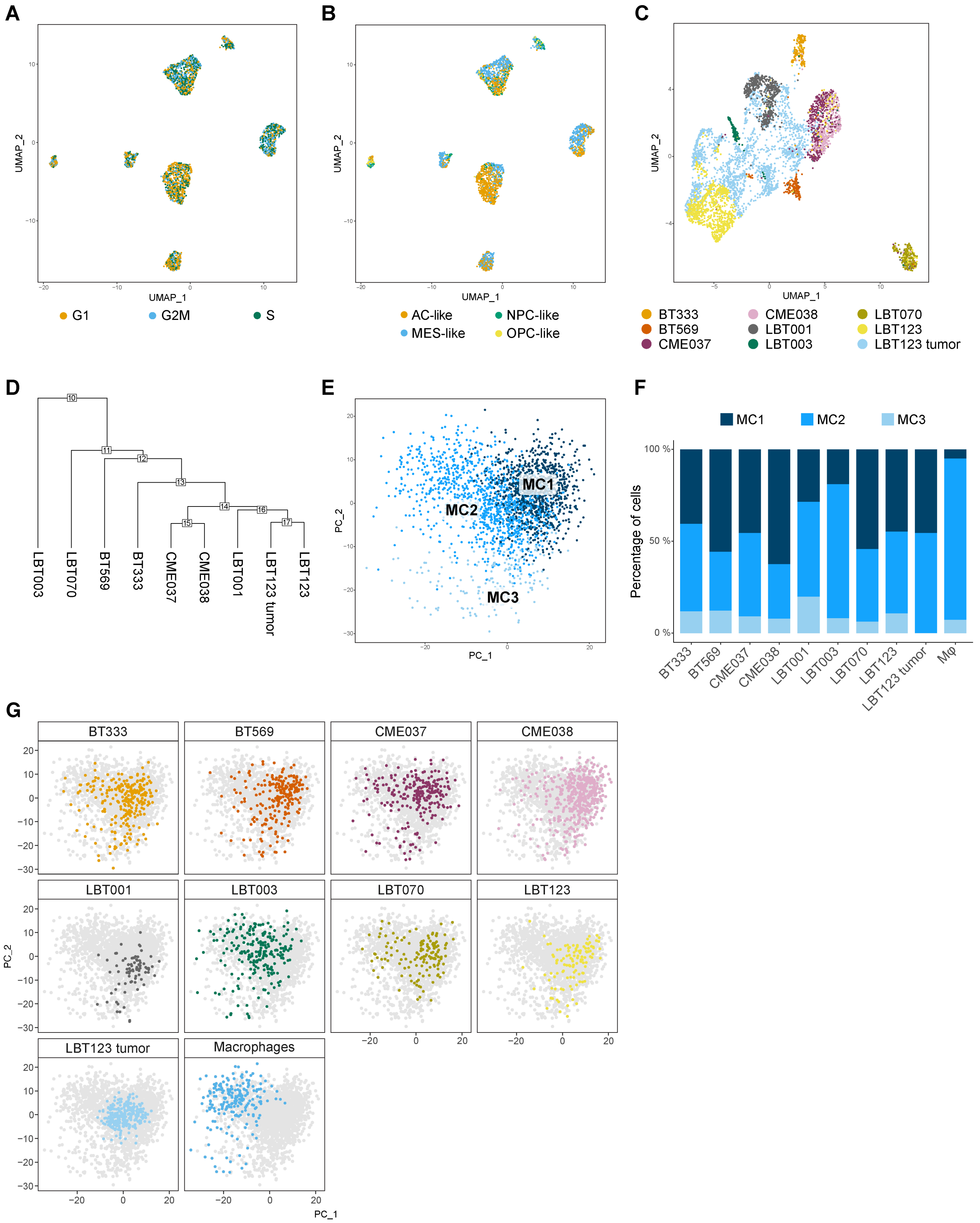


**Figure EV4.**

 © 2023 The Authors

**Figure EV5. *LGALS1* is involved in suppression of the immune system.**

A Violin plot showing *CLU* expression levels in GSCCs.

B Violin plot showing *TREM2* expression levels in macrophages ($P$ = 0.8611 (LBT003), 0.5895 (LBT070), 0.3979 (BT333), 0.6995 (LBT001), 0.0122 (CME037), 0.0526 (BT569), 0.0028 (LBT123), 2.8e-8 (CME038)).

C Representative immunofluorescence images of LBT070 *LGALS1* WT and LBT070 *LGALS1* KO cells showing expression of GAL1 (green), and cell nuclei stained by DAPI. Scale bars: 100 μm.

D, E Maximum intensity projections of a z stack of the head region of *Tg(mpeg1:mCherryF)*$^{ump2}$; *Tg(kdrl:lynEYFP)*$^{md77}$ zebrafish embryos with GFP-labeled LBT070 (D) and LBT070 *LGALS1* KO (E) tumors at 5 dpi. GBM tumor cells are shown in green, GAMs in red, and blood vessels in blue. Scale bars: 50 μm. $n$ = 9 (D) and 5 (E).

F Boxplot of GAM distance to the tumor of all GAMs within 30 μm of the tumor, at the start of 1 and 5 dpi time-lapse movies ($n$ = 257 and 99 (LBT070; 1 and 5 dpi), 481 and 83 (LBT070 *LGALS1* KO; 1 and 5 dpi); $P$ = 0.0020 (LBT070), 0.9997 (LBT070 *LGALS1* KO), 0.8013 (1 dpi), 0.0067 (5 dpi); boxes stand for 50% of the data and minima/maxima are indicated by the line ends).

Data information: The zebrafish experiments describe biological replicates, i.e. individual zebrafish embryos. The xenograft experiment was replicated twice for LBT070 *LGALS1* KO, and three times for LBT070. In (B), the $P$-values were calculated by Kruskal–Wallis test ($P$ = 2.5e-10). In (F), the $P$-values were calculated by two-way ANOVA, followed by pairwise testing with Tukey's multiple comparisons correction. ns ≥ 0.05, *$P < 0.05$, **$P < 0.01$, ****$P < 0.0001$.
Source data are available online for this figure.

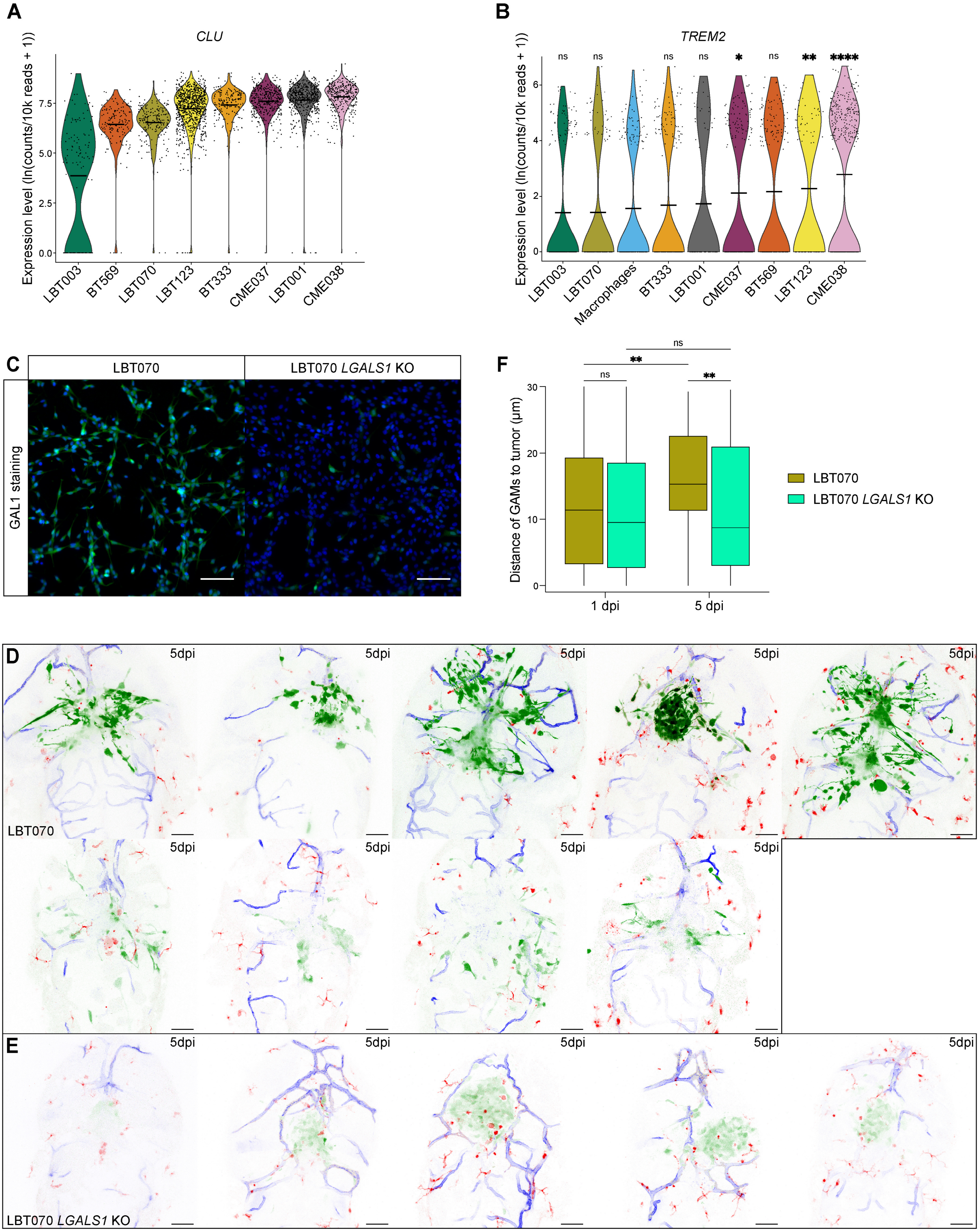


**Figure EV5.**



© 2023 The Authors
