# Supplementary figures and images for: Single‐cell profiling and zebrafish avatars reveal LGALS1 as immunomodulating target in glioblastoma
Source: EMBO Mol Med. 2023 Oct 4;15(11):e18144. doi: 10.15252/emmm.202318144 (PMC10630887; doi:10.15252/emmm.202318144)

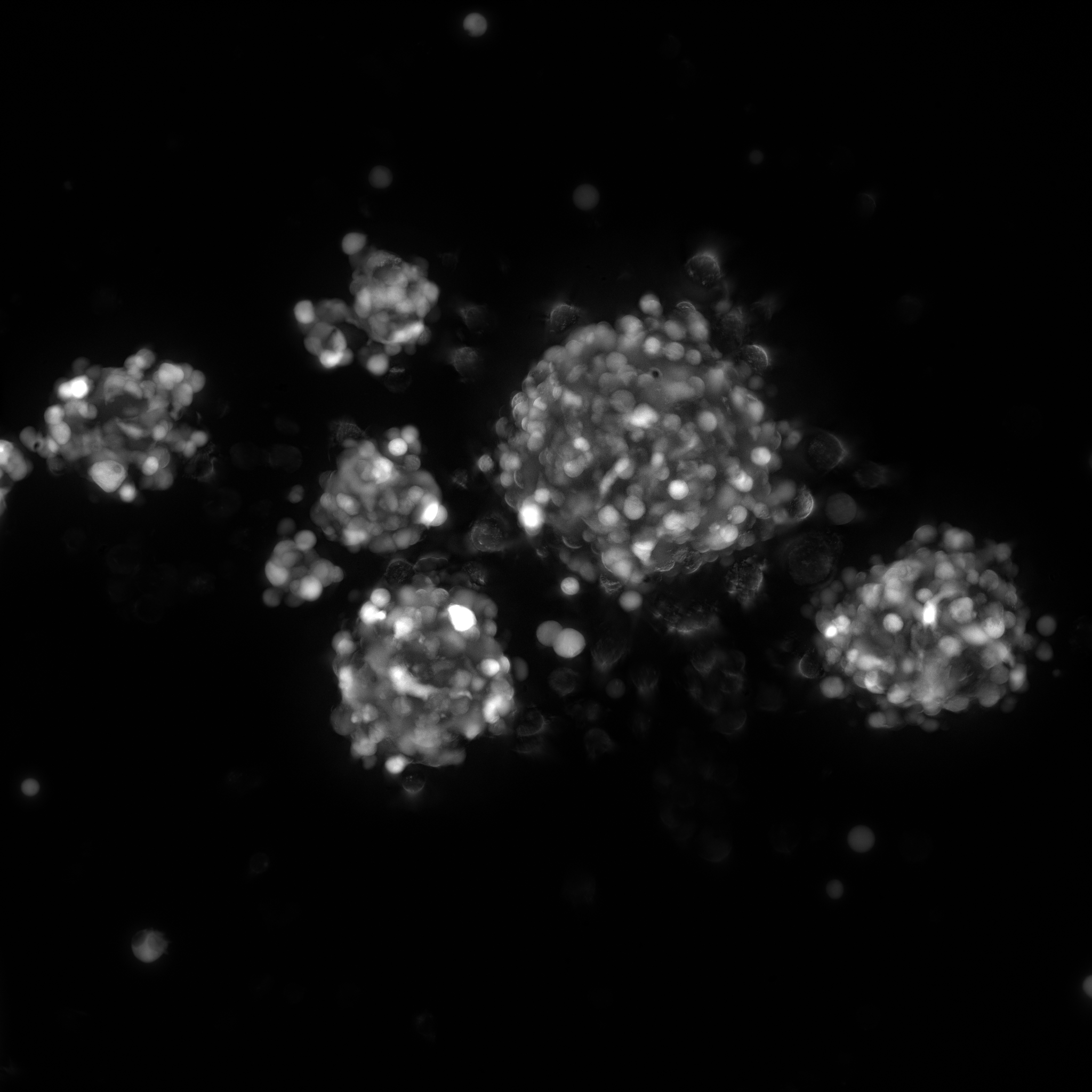

Supplement: Supplementary file 25 — Source Data for Figure 2 [file EMMM-15-e18144-s031.zip › Figure 2/2B/GSCC-macrophage co-culture.tif]

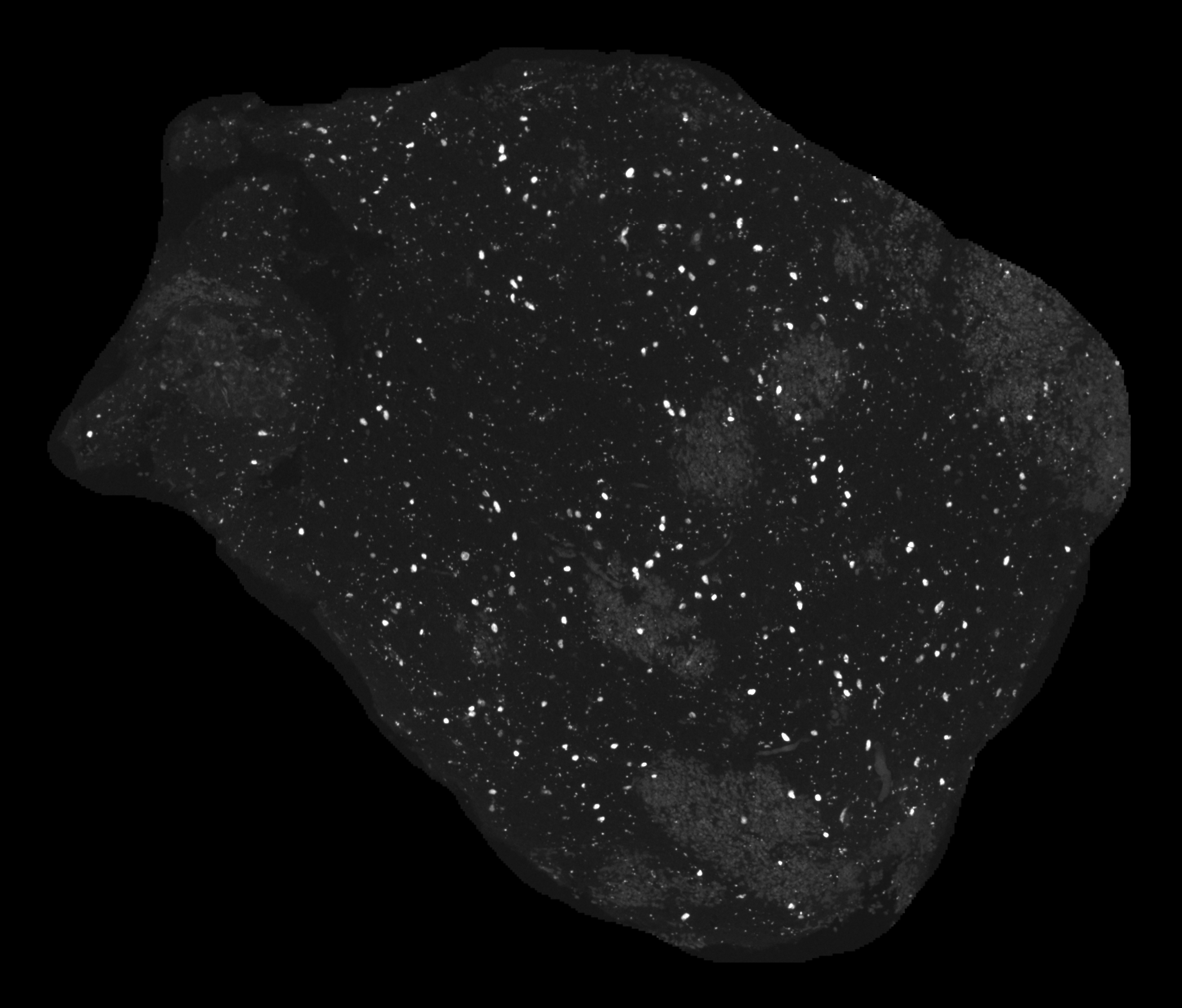

Supplement: Supplementary file 30 — Source Data for Figure 8 [file EMMM-15-e18144-s002.zip › Figure 8/8C/LBT070_selection.tif]

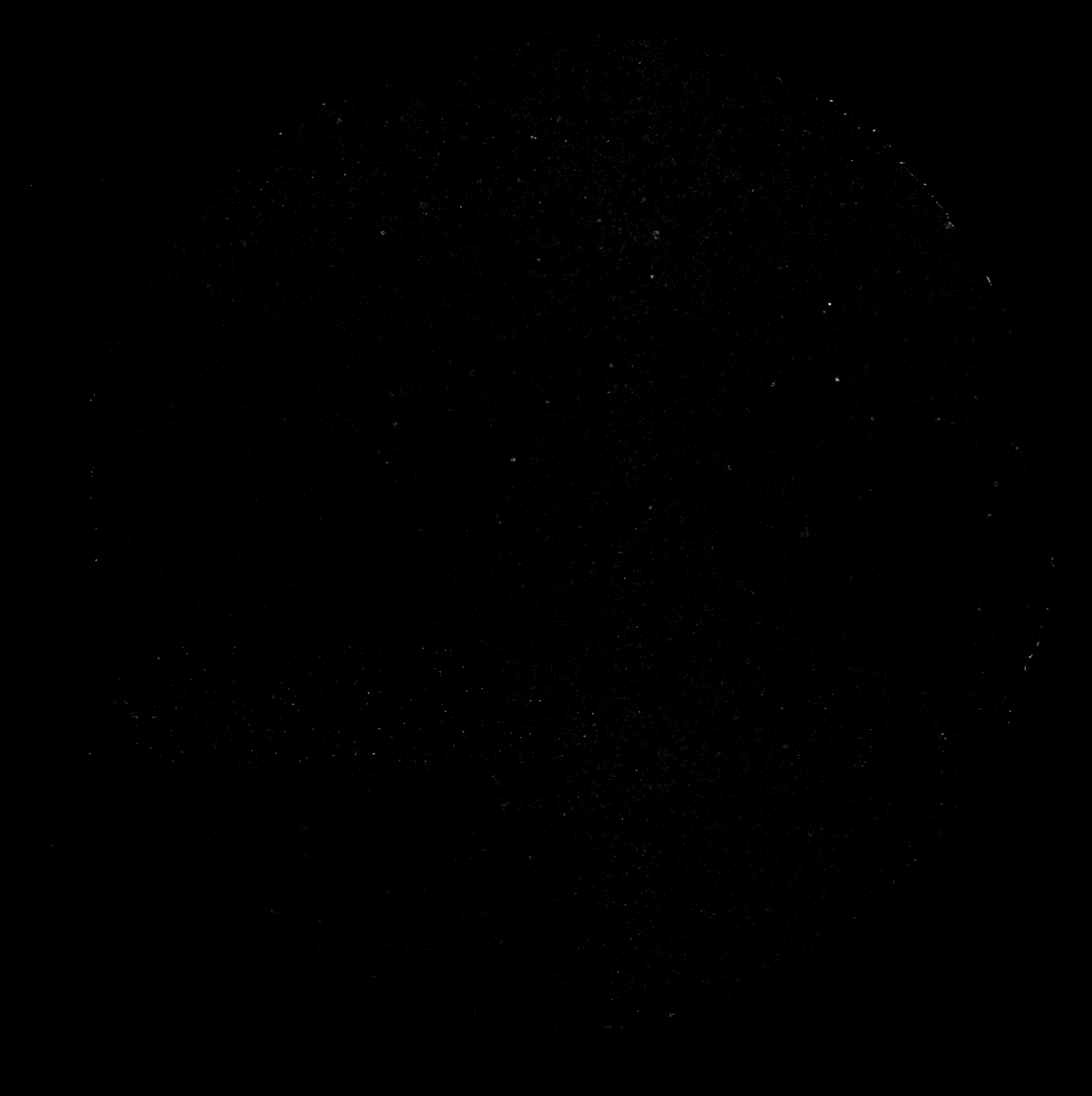

Supplement: Supplementary file 30 — Source Data for Figure 8 [file EMMM-15-e18144-s002.zip › Figure 8/8C/LBT003_processed_selection.tif]

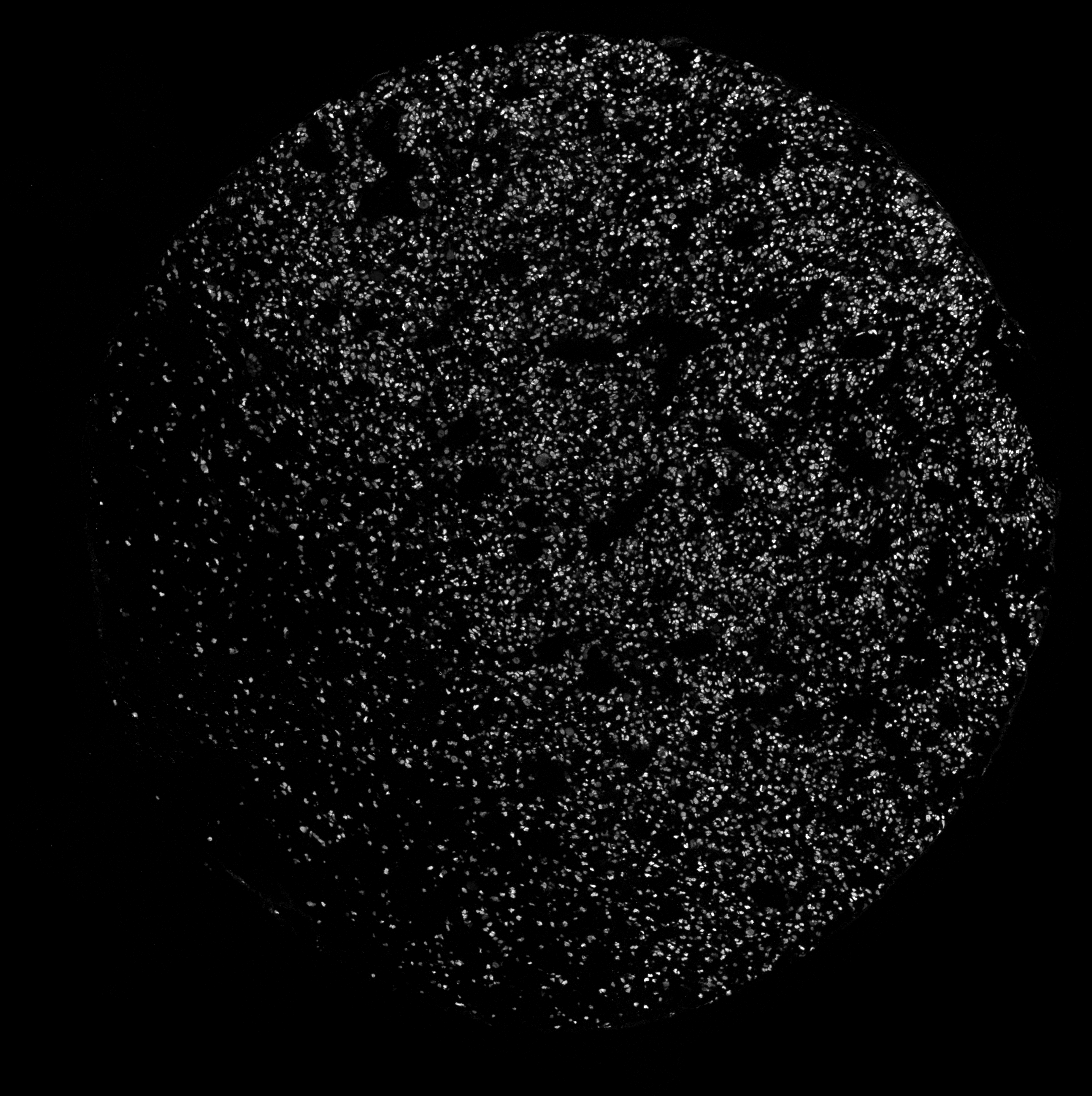

Supplement: Supplementary file 30 — Source Data for Figure 8 [file EMMM-15-e18144-s002.zip › Figure 8/8C/LBT003_selection.tif]

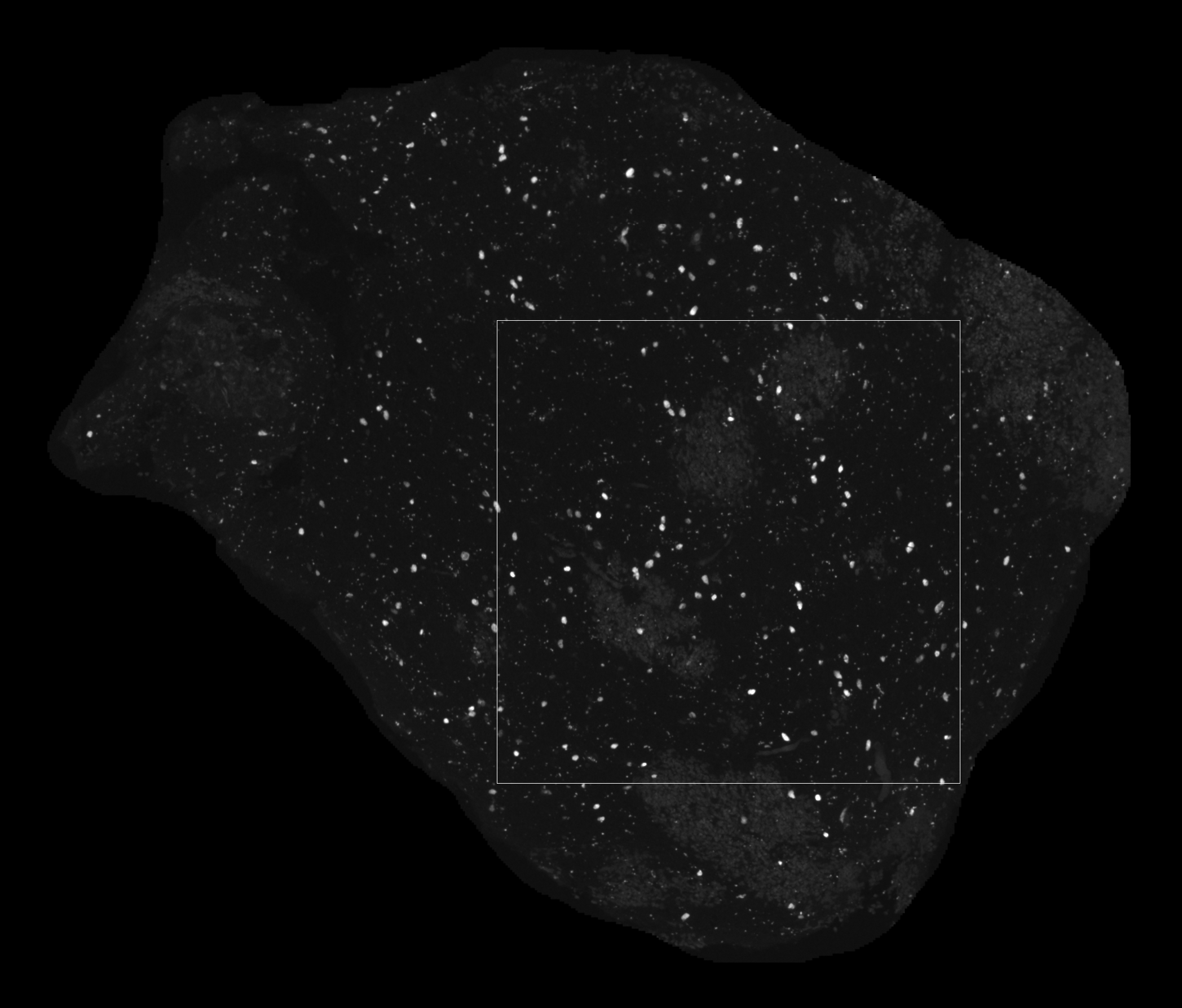

Supplement: Supplementary file 30 — Source Data for Figure 8 [file EMMM-15-e18144-s002.zip › Figure 8/8C/LBT070_processed_selection.tif]
