# Supplementary material for: Single‐cell profiling and zebrafish avatars reveal LGALS1 as immunomodulating target in glioblastoma
Source: EMBO Mol Med. 2023 Oct 4;15(11):e18144. doi: 10.15252/emmm.202318144 (PMC10630887; doi:10.15252/emmm.202318144)
Supplement: Supplementary file 31 [file EMMM-15-e18144-s022.pdf]

EMBO Molecular Medicine

Single-cell profiling and zebrafish avatars reveal LGALS1 as immunomodulating target in glioblastoma

Lise Finotto, Basiel Cole, Wolfgang Giese, Elisabeth Baumann, Annelies Claeys, Maxime Vanmechelen, Brecht Decraene, Marleen Derweduwe, Nikolina Dubroja Lakic, Gautam Shankar, Madhu Nagathihalli Kantharaju, Jan Albrecht, Ilse Geudens, Fabio Stanchi, Keith Ligon, Bram Boeckx, Diether Lambrechts, Kyle Harrington, Ludo Van Den Bosch, Steven De Vleeschouwer, Frederik De Smet, and Holger Gerhardt
DOI: 10.15252/emmm.202318144

Corresponding authors: Holger Gerhardt (Holger.Gerhardt@mdc-berlin.de) , Frederik De Smet (frederik.desmet@kuleuven.be)

Review Timeline:

Transfer from Review Commons:	9th Jun 23
Editorial Decision:	14th Jun 23
Revision Received:	1st Aug 23
Editorial Decision:	15th Aug 23
Revision Received:	29th Aug 23
Accepted:	4th Sep 23

Editor: Lise Roth

Transaction Report:

This manuscript was transferred to EMBO Molecular medicine following peer review at Review Commons.

(Note: With the exception of the correction of typographical or spelling errors that could be a source of ambiguity, letters and reports are not edited. Depending on transfer agreements, referee reports obtained elsewhere may or may not be included in this compilation. Referee reports are anonymous unless the Referee chooses to sign their reports.)

Review #1

1. Evidence, reproducibility and clarity:

Evidence, reproducibility and clarity (Required)

Summary: the paper suggested a new approach to study in vivo possible interaction between glioblastoma cells and glioblastoma associated macrophages. By using single cells transcriptome profiling and in vitro and in vivo functional experiments the authors also suggested LGALS1 as possible key factor in the suppression of the immune system and a new target for immune modulation in glioma patients. The experimental plan is well described, and the results are beautifully presented using images, clear drawings, and videos.

Major comments: none

Minor comments: The number of zebrafish embryos analyzed after the xenograft is highly variable (e.g. 3-18; 4-22 in Figure 6). These numbers can be reported in the results section (not only in the legends) and the authors may comment on them in the discussion. The reproducibility of the xenotransplant experiments is always challenging as it is quite difficult to inject the same number of cells in every embryo and to have the same survival rate of injected cells and of transplanted embryos. For these reasons the volume of each xenograft can vary significantly in different embryos and in different experimental session. Accordingly, the number of macrophages associated to the tumor can vary and the statistical analysis can be deeply influenced by the number of replicates for each experimental group (a group with 3 embryos is very different in term of quality and quantity of information in respect to a group of 18 embryos). It could be useful for the reader, who has no experience in this technique, be aware of the advantages and disadvantages of the procedure including the possible influence of the temperature (34{degree sign}C instead of 37{degree sign}C) on the embryo survival and the replication rate of glioma cells or macrophages behavior. Comment on these aspects does not weaken the power and the relevance of the model but unveil the critical aspects that every scientist has to evaluate before planning these kinds of experiments.
An aspect that could be interesting to address, to further validate the avatar model, is to monitor the level of pro-inflammatory cytokines (Tumor Necrosis Factor and Interleukin 1, 6, and 8) that are expressed at basal level in the early developing zebrafish embryos. Do their expression level increase after the xenotransplantation? Can the zebrafish cytokines affect the behavior of glioma associated macrophages (i.e. macrophages polarization)?

2. Significance:

Significance (Required)

Strengths and limitation. The manuscript is the result of a well-orchestrated effort to dissect a biological problem by complementary approaches and provide new data with high impact translational value. The image processing pipeline developed by the authors is a step forward in the in vivo analysis of cells interaction in living embryos. The identification of LGALS1 as a potential target for immune modulation can support the development of new therapeutical strategy implementing chemo- or immunotherapy protocols. The described

zebrafish avatar can represent a new tool for personalized drug testing recapitulating in a in vivo model the heterogeneity of GBM found in patients.

Audience: All the scientist interested in cell biology, cancer cell biology, imaging techniques, translational medicine, in vivo models for cancer research, precision medicine.

Reviewer expertise: applied developmental biology

3. How much time do you estimate the authors will need to complete the suggested revisions:

Estimated time to Complete Revisions (Required)

(Decision Recommendation)

Less than 1 month

4. *Review Commons* values the work of reviewers and encourages them to get credit for their work. Select 'Yes' below to register your reviewing activity at Web of Science Reviewer Recognition Service (formerly Publons); note that the content of your review will not be visible on Web of Science.

Web of Science Reviewer Recognition

Yes

Review #2

1. Evidence, reproducibility and clarity:

Evidence, reproducibility and clarity (Required)

Finotto et al aim to address the polarisation of macrophages within GBM in their study. To do this, they have developed two different models. The first model is an in-vitro co-culture model of patient derived GSC lines and human monocyte derived macrophages. This model was used for single cell sequencing to understand the transcriptomic changes of macrophages upon contact to GBM cells. The second model is a zebrafish xenograft model. Here GFP labeled GBM cells were transplanted into the larval zebrafish ventricle. These experiments were done in the transgenic mpeg zebrafish which allowed to monitor responses of macrophages in vivo.

In my opinion both models are not sophisticated enough to draw solid conclusions on macrophage polarisation in GBM. The in vitro model is highly artificial and is far from the complex situation in GBM. Within GBM the GAM population represents a heterogenous mix of resident microglia and infiltrating macrophages. These are influenced by the heterogeneous environment (which consists of tumour cells but also other host cells) and show diverse transcriptomic adaptations as shown in rodent models as well as sequencing studies of patient derived tumour samples. Studying monocyte derived macrophages in vitro does not provide any reliable insight.

Although the zebrafish can be a great model to understand the progression of tumours and the role of immune cells, I don't think that the model developed by the authors is suitable to address their questions. Transplantation of GBM cells into the the ventricle of larval zebrafish doesn't seem to be the right approach here. The poor survival of the transplanted cells is a clear indication of that. Many other groups have reported growth and proliferation of human cancer cells in the larval zebrafish. Direct transplantation into the brain parenchyma would be the better approach here. The brain parenchyma would provide the right environment for the GBM cells including a resident microglial population. This would also allow to study the complex mix of microglia and infiltrating macrophages in the context of GBM.

2. Significance:

Significance (Required)

In my opinion both models are not sophisticated enough to draw solid conclusions on macrophage polarisation in GBM.

3. How much time do you estimate the authors will need to complete the suggested revisions:

Estimated time to Complete Revisions (Required)

(Decision Recommendation)

More than 6 months

4. *Review Commons* values the work of reviewers and encourages them to get credit for their work. Select 'Yes' below to register your reviewing activity at [Web of Science Reviewer Recognition Service]() (formerly Publons); note that the content of your review will not be visible on Web of Science.

Web of Science Reviewer Recognition

No

Review #3

1. Evidence, reproducibility and clarity:

Evidence, reproducibility and clarity (Required)

In this study, Finoto and colleagues developed patient-derived Glioblastoma (GBM) stem cell cultures from 7 patients. These GBM stem cell cultures were either co-cultured in vitro with human macrophages combined with single-cell RNA sequencing or injected into the orthotopic zebrafish xenograft to study live GBM-macrophage/microglia interactions. Authors aimed at studying tumor heterogeneity and GBM-associated macrophages (GAMs) which often exhibit immunosuppressive features that promote tumor progression. Their analyses revealed substantial heterogeneity across GBM patients in GBM-induced macrophages polarization and the ability to attract and activate GAMs - features that correlated with patient survival. Also authors show 3 distinct macrophage subclusters (MC1-3), highlighting that the simple M1/M2 polarization phenotypes is too reductive and there are no clear "markers". Authors associate these profiles with morphology and macrophage behaviour.

Differential gene expression analysis, immunohistochemistry on original tumor samples, and knock-out experiments in zebrafish subsequently identified / confirmed that LGALS1 as a primary regulator of immunosuppression. Cheng et (DOI: 10.1002/ijc.32102) had previously shown the immunosuppression effect of LGALS1 - but this work shows as a proof of concept that the authors approach is a valuable and interesting approach to find immune regulators.

Major comments

In general claims are supported by date - very carefully presented and well characterized data with numbers, stats. It is an interesting descriptive study that illustrates the complexity and diversity of glioblastoma and the induced TME.
I just have a few comments or clarifications that I would like to have elucidated:
1. I did not understand why not single cell sequence the original tumor - without in vitro passaging and have the original patient population of MACs/microglia and monocytes sequenced? In other words why sequence the in vitro system-with its inherent caveats of in vitro culturing and not the original tumor? Can you please clarify
2. Mac signatures - out of curiosity- authors could not find TNFa and IFN signatures in any population?
3. Fig. 8 please show controls side by side with the KO
4. Figure 5 if each pair of images are separated and have the legend on top would be easier to read and follow.

2. Significance:

Significance (Required)

It is a very interesting study, carefully designed and performed that highlights the heterogeneity of glioblastoma and how GBM can modulate the macrophage population into 3 different subsets. This study constitutes a proof of concept of the combination of and in vitro approach and an in vivo approach to find new players and treatments in glioblastoma.

I believe that it would be important and interesting to have a the original tumor sequenced to compare to the in vitro platform and understand how the in vitro selection impacts on the tumor biology and even if it changes the heterogeneity and differential composition of the tumor and macrophage profiles.

3. How much time do you estimate the authors will need to complete the suggested revisions:

Estimated time to Complete Revisions (Required)

(Decision Recommendation)

Cannot tell / Not applicable

4. *Review Commons* values the work of reviewers and encourages them to get credit for their work. Select 'Yes' below to register your reviewing activity at Web of Science Reviewer Recognition Service (formerly Publons); note that the content of your review will not be visible on Web of Science.

Web of Science Reviewer Recognition

Yes

Revision Plan

Manuscript number: RC-2023-01982
Corresponding author(s): Holger, Gerhardt & Frederik, De Smet

1. General Statements

We believe that there is an urgent need for good preclinical models that are scalable, flexible and screenable, which could ultimately be used to test new drugs or guide patient treatment in a personalized manner. In this work, we have focused on the differences between several GBM patients in scalable and screenable models, where perturbations (CRISPR KO, compounds, etc.) can be applied. We agree that our models are simplified representations of the clinical situation, as are all preclinical models, and like all models, they have advantages and disadvantages. We deliberately use simplified models to achieve the goals of scalability and screenability, where, under controlled conditions, differential behavior of GBM tumor cells derived from various patients can be systematically compared in how they affect a particular environment (*i.e.* the zebrafish brain and cultured macrophages). Obviously, such observations require further validation in clinical samples, which we have done. Furthermore, other research groups have shown that preclinical models are very useful to gain new insights in the search for new GBM treatments. We would like to emphasize that we are not making any strong claims regarding translation to the clinic. We are simply showing that these models are very useful for GBM research, while providing new opportunities to identify promising immunomodulating targets. The role of the target we identified, *LGALS1*, has been previously demonstrated by others, validating our approach. Finally, we also confirm our findings in patient material, and our KO experiments show a clear and non-ignorable phenotype, proving that our models are indeed useful.

2. Description of the planned revisions

Reviewer #1: An aspect that could be interesting to address, to further validate the avatar model, is to monitor the level of pro-inflammatory cytokines (Tumor Necrosis Factor and Interleukin 1, 6, and 8) that are expressed at basal level in the early developing zebrafish embryos. Do their

expression level increase after the xenotransplantation? Can the zebrafish cytokines affect the behavior of glioma associated macrophages (i.e. macrophages polarization)?

Response: This is an interesting point, indeed. We have injected murine melanoma (B16) cells into *Tg(mpeg1:mCherry-F); Tg(TNFa:eGFP-F)* embryos, a TNFα reporter line. Some (but not all) macrophages expressed TNFa and their expression decreased over time, which is consistent with previous reports (Póvoa *et al*, 2021). We further observed that TNFα-expressing macrophages mostly had a round, "tumor-attacking" phenotype. This is in line with our hypothesis that the tumor induces a phenotype switch in GAMs. Of note, we did not see TNFα expression in the rest of the brain tissue. We would be happy to add this data if deemed useful.
We did not investigate other cytokines in the developing zebrafish, but we believe this is not essential for the following reasons: We are mainly interested in the differences between the patient-derived GBM stem cell cultures (GSSCs), and since they are all used in the same avatar model, we expect that if zebrafish cytokines would have an effect on GAMs and their polarization, this effect would be consistent in all avatars, and can thus be ignored when comparing different GSCCs. More importantly, our findings in the zebrafish avatar model were consistent with those in the *in vitro* model. We observed the same phenotype switch in the co-culture model, indicating that the key interaction is between tumor cells and macrophages.

<u>*Reviewer #3:*</u> *I did not understand why not single cell sequence the original tumor - without in vitro passaging and have the original patient population of MACs/microglia and monocytes sequenced? In other words, why sequence the in vitro system - with its inherent caveats of in vitro culturing and not the original tumor? Can you please clarify? I believe that it would be important and interesting to have the original tumor sequenced to compare to the in vitro platform and understand how the in vitro selection impacts on the tumor biology and even if it changes the heterogeneity and differential composition of the tumor and macrophage profiles.*

Response: We agree with Reviewer #3 that our *in vitro* model does indeed have caveats inherent to patient-derived cell culture models. However, we chose this model to specifically focus on the reciprocal interaction between GBM tumor cells and macrophages in a way that also allows us to investigate how perturbations affect these interactions. This is not possible when using original tumors (*e.g.* we cannot make KO cells, as we did for *LGALS1*, and study the effects of genes of interest). (See also the response to the comment of Reviewer #2 in Section 4.)
We do have scRNAseq data from one original tumor sample (LBT123) that is currently being analyzed. Unfortunately, scRNAseq is not available for the other tumor samples. Also, for some of the patients, there is no original material left to use for sequencing. For LBT123, we will compare the scRNAseq data from the original tumor with the *in vitro* data from the co-culture model.

<u>*Reviewer #3:*</u> *Mac signatures - out of curiosity - authors could not find TNFa and IFN signatures in any population?*

Response: Our analyses did not reveal TNF or IFN as cluster signature genes. However, we did find that TNF expression was slightly higher in MC2, the pro-inflammatory macrophages, although

still at low levels. We did not find IFN expression in the macrophage subclusters, but we did find low expression of some IFN receptors. We found a gradient for IFNGR1 with the highest expression in MC3, followed by MC1 and the lowest expression in MC2. IFNGR2 was expressed at slightly higher levels in MC1 compared to the other subclusters. IFNAR1 and IFNAR2 were expressed at comparable low levels in all subclusters. Finally, IFNLR1 expression was higher in MC3 compared to the other two macrophage subclusters. Considering the overall low expression of IFN receptors, we believe that the differences in expression are rather negligible. Furthermore, it has been previously shown that IFN exerts its anti-tumor effect primarily through the responsiveness of endothelial cells and not of myeloid cells, such as macrophages (Kammertoens *et al*, 2017). Since vascular cells were not present in the co-culture model, low IFN receptor expression is not surprising. We are happy to investigate this in more detail and include it if deemed useful.

3. Description of the revisions that have already been incorporated in the transferred manuscript

<u>Reviewer #1</u>: *The number of zebrafish embryos analyzed after the xenograft is highly variable (e.g. 3-18; 4-22 in Figure 6). These numbers can be reported in the results section (not only in the legends) and the authors may comment on them in the discussion. The reproducibility of the xenotransplant experiments is always challenging as it is quite difficult to inject the same number of cells in every embryo and to have the same survival rate of injected cells and of transplanted embryos. For these reasons the volume of each xenograft can vary significantly in different embryos and in different experimental session. Accordingly, the number of macrophages associated to the tumor can vary and the statistical analysis can be deeply influenced by the number of replicates for each experimental group (a group with 3 embryos is very different in term of quality and quantity of information in respect to a group of 18 embryos). It could be useful for the reader, who has no experience in this technique, be aware of the advantages and disadvantages of the procedure including the possible influence of the temperature (34°C instead of 37°C) on the embryo survival and the replication rate of glioma cells or macrophages behavior. Comment on these aspects does not weaken the power and the relevance of the model but unveil the critical aspects that every scientist has to evaluate before planning these kinds of experiments.*

Response: We agree with Reviewer #1 that the zebrafish avatar model is challenging, and it is difficult to obtain reproducible tumor sizes and survival rates. To be even more transparent about this, we have added a few sentences about the variable *n* number in the Results section and a critical comment about it in the Discussion section.

<u>Reviewer #3:</u> *Cheng et (DOI: 10.1002/ijc.32102) had previously shown the immunosuppression effect of LGALS1 - but this work shows as a proof of concept that the authors approach is a valuable and interesting approach to find immune regulators.*

Response: We fully agree with Reviewer #3. In fact, the immunosuppressive role of *LGALS1* has already been described by several research groups (Van Woensel *et al*, 2017; Verschuere *et al*,

2014), which indeed proves that our approach is valid. The reference cited by the reviewer was already included in the manuscript, along with other references.

<u>Reviewer #3:</u> *Fig. 8: please show controls side by side with the KO.*

Response: We thank Reviewer #3 for this comment. We are not quite sure which panel the reviewer is referring to. If it is panel F, we agree with Reviewer #3 and have changed the order of the bars in the revised version. If it is panel E, the corresponding control images are shown in Figure 5I. Since we believe that these images should not be repeated, we have added a figure reference to Figure 5I in the figure legend of Figure 8, in addition to the figure reference already provided in the text. Furthermore, images of all embryos are presented side by side in Figure S8D-E.

4. Description of analyses that authors prefer not to carry out

<u>Reviewer #2:</u> *In my opinion both models are not sophisticated enough to draw solid conclusions on macrophage polarisation in GBM. The in vitro model is highly artificial and is far from the complex situation in GBM. Within GBM the GAM population represents a heterogenous mix of resident microglia and infiltrating macrophages. These are influenced by the heterogeneous environment (which consists of tumour cells but also other host cells) and show diverse transcriptomic adaptations as shown in rodent models as well as sequencing studies of patient derived tumour samples. Studying monocyte derived macrophages in vitro does not provide any reliable insight.*

Response: We understand the reviewer's concern about the complexity of our *in vitro* model. However, as explained in "General Statements", these simple models are needed to gain more insight into the complex *in vivo* situation. Others have demonstrated their usefulness in the past (C. Jayakrishnan *et al*, 2019; Zhou *et al*, 2022; Hubert *et al*, 2016; Chen *et al*, 2020; Coniglio *et al*, 2016; Li *et al*, 2022). Moreover, it may be advantageous to look at only two different cell types and unravel their reciprocal interaction, without the influence of other cell types, making it too complex to draw conclusions. We acknowledge that GAMs are a heterogeneous mix of both microglia and bone marrow-derived macrophages. Considering that bone marrow-derived macrophages have been shown to play an important role in tumor progression and are by far the most abundant immune cell population in GBM tumors (which even increases in recurrent GBM) (Pombo Antunes *et al*, 2021; Abdelfattah *et al*, 2022), we chose to focus initially on bone marrow-derived macrophages. Notably, it has already been reported that microglia were associated with significantly better survival, suggesting that they are anti-tumorigenic, whereas macrophages were associated with worse survival, suggesting that they are pro-tumorigenic (Pombo Antunes *et al*, 2021; Abdelfattah *et al*, 2022). This justifies our approach to focus on this cell type. Furthermore, although this model may be rather simplistic, it allowed us to screen different GSCCs side by side in a standardized way, through which we found an apparent phenotype switch within the macrophages, even without the complex interplay with other cell types. Because the results obtained using the *in vitro* model were also confirmed in GBM patient material and KO

experiments in the zebrafish avatar model, our work shows that reliable and important insights can be derived. This, combined with its simplicity, makes our co-culture model an exceptionally relevant model that is scalable, screenable and allows us to study the effect of perturbations. Finally, the immunosuppressive role of the target we identified using this model, *LGALS1*, has been previously demonstrated by others (Verschuere *et al*, 2014; Van Woensel *et al*, 2017; Chen *et al*, 2019), which proves our approach is valid.

<u>Reviewer #2:</u> *Although the zebrafish can be a great model to understand the progression of tumours and the role of immune cells, I don't think that the model developed by the authors is suitable to address their questions. Transplantation of GBM cells into the the ventricle of larval zebrafish doesn't seem to be the right approach here. The poor survival of the transplanted cells is a clear indication of that. Many other groups have reported growth and proliferation of human cancer cells in the larval zebrafish. Direct transplantation into the brain parenchyma would be the better approach here. The brain parenchyma would provide the right environment for the GBM cells including a resident microglial population. This would also allow to study the complex mix of microglia and infiltrating macrophages in the context of GBM.*

Response: The reviewer does not specify which articles have reported growth and proliferation of human cancer cells in zebrafish larvae. Most research groups reporting this, did not follow tumor growth/proliferation over time or used immortalized cell lines (Vargas-Patron *et al*, 2019; Pan *et al*, 2020; Pudelko *et al*, 2018; Breznik *et al*, 2017; Vittori *et al*, 2017; Hamilton *et al*, 2016), which obviously have a much higher proliferation rate than the patient-derived cell lines used in this work. Second, although the number of patient-derived tumor cells decreases over time, we observed a clear invasive and migratory behavior, indicating that the human tumor cells reside well in the zebrafish microenvironment. Furthermore, it is important to note that the zebrafish avatars are grown at 34°C, a temperature that is suboptimal for tumor cell growth. The tumor cells still proliferate, albeit at a lower rate than at 37°C.
To our knowledge, there is only one publication that reports the growth of patient-derived GBM tumors over time (Almstedt *et al*, 2022). However, here, zebrafish embryos were grown at 33°C. Also, prior to injection, patient-derived GBM cells were resuspended in medium containing polyvinylpyrrolidone, a polymer that enhances extracellular matrix deposition and cell proliferation. Furthermore, the authors observed substantial differences in proliferative capacity, ranging from growth to decline of signal, and represented only two patient-derived cell lines with growing tumors. Similar to our findings, another article has demonstrated that injected patient-derived GBM tumor cells progressively underwent mitotic arrest, while maintaining an invasive and aggressive growth pattern (Rampazzo *et al*, 2013).
Although the tumor cells are injected into the hindbrain ventricle, they end up in the brain parenchyma, as evidenced by the presence of the typical brain vasculature of the zebrafish embryo. Notably, in *Tg(mpeg1:mCherryF)*^{ump2} zebrafish embryos, both macrophages and microglia are labeled with mCherry, meaning that we have studied both cell types in our zebrafish avatar model. Therefore, we consider the reviewer's comment to be unfounded.

<u>Reviewer #3:</u> *Figure 5: if each pair of images are separated and have the legend on top would be easier to read and follow.*

Response: We appreciate the comment that the figure should be intuitively easy to read and follow. However, we have chosen a compromise between overview and visibility of details (*e.g.* morphological features of GAMs). Since this figure already has the maximum width, the images would become smaller if they needed to be separated. Reducing the size would compromise the visibility of important details.

<u>References:</u>

Abdelfattah N, Kumar P, Wang C, Leu JS, Flynn WF, Gao R, Baskin DS, Pichumani K, Ijare OB, Wood SL, *et al* (2022) Single-cell analysis of human glioma and immune cells identifies S100A4 as an immunotherapy target. *Nat Commun* 13

Almstedt E, Rosen E, Gloger M, Stockgard R, Hekmati N, Koltowska K, Krona C & Nelander S (2022) Real-time evaluation of glioblastoma growth in patient-specific zebrafish xenografts. *Neuro Oncol* 24: 726–738

Breznik B, Motaln H, Vittori M, Rotter A & Turnšek TL (2017) Mesenchymal stem cells differentially affect the invasion of distinct glioblastoma cell lines. *Oncotarget* 8: 25482–25499

C. Jayakrishnan P, H. Venkat E, M. Ramachandran G, K. Kesavapisharady K, N. Nair S, Bharathan B, Radhakrishnan N & Gopala S (2019) In vitro neurosphere formation correlates with poor survival in glioma. *IUBMB Life* 71: 244–253

Chen JWE, Lumibao J, Leary S, Sarkaria JN, Steelman AJ, Gaskins HR & Harley BAC (2020) Crosstalk between microglia and patient-derived glioblastoma cells inhibit invasion in a three-dimensional gelatin hydrogel model. *J Neuroinflammation* 17

Chen Q, Han B, Meng X, Duan C, Yang C, Wu Z, Magafurov D, Zhao S, Safin S, Jiang C, *et al* (2019) Immunogenomic analysis reveals LGALS1 contributes to the immune heterogeneity and immunosuppression in glioma. *Int J Cancer* 145: 517–530

Coniglio S, Miller I, Symons M & Segall JE (2016) Coculture assays to study macrophage and microglia stimulation of glioblastoma invasion. *Journal of Visualized Experiments* 2016

Hamilton L, Astell KR, Velikova G & Sieger D (2016) A zebrafish live imaging model reveals differential responses of microglia toward glioblastoma cells in vivo. *Zebrafish* 13: 523–534

Hubert CG, Rivera M, Spangler LC, Wu Q, Mack SC, Prager BC, Couce M, McLendon RE, Sloan AE & Rich JN (2016) A three-dimensional organoid culture system derived from human glioblastomas recapitulates the hypoxic gradients and cancer stem cell heterogeneity of tumors found in vivo. *Cancer Res* 76: 2465–2477

Kammertoens T, Friese C, Arina A, Idel C, Briesemeister D, Rothe M, Ivanov A, Szymborska A, Patone G, Kunz S, *et al* (2017) Tumour ischaemia by interferon-γ resembles physiological blood vessel regression. *Nature* 545: 98–102

Li H, Yan X & Ou S (2022) Correlation of the prognostic value of FNDC4 in glioblastoma with macrophage polarization. *Cancer Cell Int* 22

Pan H, Xue W, Zhao W & Schachner M (2020) Expression and function of chondroitin 4-sulfate and chondroitin 6-sulfate in human glioma. *FASEB Journal* 34: 2853–2868

Pombo Antunes AR, Scheyltjens I, Lodi F, Messiaen J, Antoranz A, Duerinck J, Kancheva D, Martens L, De Vlaminck K, Van Hove H, *et al* (2021) Single-cell profiling of myeloid cells in glioblastoma across species and disease stage reveals macrophage competition and specialization. *Nat Neurosci* 24: 595–610

Póvoa V, Rebelo de Almeida C, Maia-Gil M, Sobral D, Domingues M, Martinez-Lopez M, de Almeida Fuzeta M, Silva C, Grosso AR & Fior R (2021) Innate immune evasion revealed in a colorectal zebrafish xenograft model. *Nat Commun* 12

Pudelko L, Edwards S, Balan M, Nyqvist D, Al-Saadi J, Dittmer J, Almlöf I, Helleday T & Bräutigam L (2018) An orthotopic glioblastoma animal model suitable for high-throughput screenings. *Neuro Oncol* 127: 415

Rampazzo E, Persano L, Pistollato F, Moro E, Frasson C, Porazzi P, Della Puppa A, Bresolin S, Battilana G, Indraccolo S, *et al* (2013) Wnt activation promotes neuronal differentiation of glioblastoma. *Cell Death Dis* 4

Van Woensel M, Mathivet T, Wauthoz N, Rosière R, Garg AD, Agostinis P, Mathieu V, Kiss R, Lefranc F, Boon L, *et al* (2017) Sensitization of glioblastoma tumor micro-environment to chemo- and immunotherapy by Galectin-1 intranasal knock-down strategy. *Sci Rep* 7: 1–14

Vargas-Patron LA, Agudelo-Dueñãs N, Madrid-Wolff J, Venegas JA, González JM, Forero-Shelton M & Akle V (2019) Xenotransplantation of human glioblastoma in zebrafish larvae: in vivo imaging and proliferation assessment. *Biol Open* 8

Verschuere T, Toelen J, Maes W, Poirier F, Boon L, Tousseyn T, Mathivet T, Gerhardt H, Mathieu V, Kiss R, *et al* (2014) Glioma-derived galectin-1 regulates innate and adaptive antitumor immunity. *Int J Cancer* 134: 873–884

Vittori M, Breznik B, Hrovat K, Kenig S & Lah TT (2017) RECQ1 helicase silencing decreases the tumour growth rate of U87 glioblastoma cell xenografts in zebrafish embryos. *Genes (Basel)* 8

Zhou F, Shi Q, Fan X, Yu R, Wu Z, Wang B, Tian W, Yu T, Pan M, You Y, *et al* (2022) Diverse macrophages constituted the glioma microenvironment and influenced by PTEN status. *Front Immunol* 13

14th Jun 2023

Dear Dr. Gerhardt,

Thank you for the submission of your manuscript to our editorial offices. I have now had the opportunity to read it, together with the referees' reports and your rebuttal letter, and to discuss them with the other members of our editorial team.

We agree that the study fits the scope of the journal, and we appreciate that you are willing to address most of the points raised by reviewers #1 and #3. We thus encourage you to submit a revised version of your manuscript, including the modifications and revisions described in your point-by-point letter. Regarding the concerns from reviewer #2 on the models used in the study, please discuss them in the point by point letter.

If you would like to discuss further the points raised by the referees, I am available to do so via email or video. Let me know if you are interested in this option.

Acceptance of the manuscript will entail a second round of review. EMBO Molecular Medicine encourages a single round of revision only and therefore, acceptance or rejection of the manuscript will depend on the completeness of your responses included in the next, final version of the manuscript. For this reason, and to save you from any frustrations in the end, I would strongly advise against returning an incomplete revision.

***

When submitting your revised manuscript, please carefully review the instructions that follow below. Failure to include requested items will delay the evaluation of your revision:

We require:

1) A .docx formatted version of the manuscript text (including legends for main figures, EV figures and tables). Please make sure that the changes are highlighted to be clearly visible.

2) Individual production quality figure files as .eps, .tif, .jpg (one file per figure). For guidance, download the 'Figure Guide PDF' (https://www.embopress.org/page/journal/17574684/authorguide#figureformat).

3) A .docx formatted letter INCLUDING the reviewers' reports and your detailed point-by-point responses to their comments. As part of the EMBO Press transparent editorial process, the point-by-point response is part of the Review Process File (RPF), which will be published alongside your paper.

4) A complete author checklist, which you can download from our author guidelines (https://www.embopress.org/page/journal/17574684/authorguide#submissionofrevisions). Please insert information in the checklist that is also reflected in the manuscript. The completed author checklist will also be part of the RPF.

5) It is mandatory to include a 'Data Availability' section after the Materials and Methods. Before submitting your revision, primary datasets produced in this study need to be deposited in an appropriate public database, and the accession numbers and database listed under 'Data Availability'. Please remember to provide a reviewer password if the datasets are not yet public (see https://www.embopress.org/page/journal/17574684/authorguide#dataavailability).
In case you have no data that requires deposition in a public database, please state so in this section. Note that the Data Availability Section is restricted to new primary data that are part of this study.

6) For data quantification: please specify the name of the statistical test used to generate error bars and P values, the number (n) of independent experiments (specify technical or biological replicates) underlying each data point and the test used to calculate p-values in each figure legend. The figure legends should contain a basic description of n, P and the test applied. Graphs must include a description of the bars and the error bars (s.d., s.e.m.).

7) We would also encourage you to include the source data for figure panels that show essential data. Numerical data should be provided as individual .xls or .csv files (including a tab describing the data). For blots or microscopy, uncropped images should be submitted (using a zip archive if multiple images need to be supplied for one panel). Additional information on source data and instruction on how to label the files are available at
.

8) Our journal encourages inclusion of *data citations in the reference list* to directly cite datasets that were re-used and obtained from public databases. Data citations in the article text are distinct from normal bibliographical citations and should

directly link to the database records from which the data can be accessed. In the main text, data citations are formatted as follows: "Data ref: Smith et al, 2001" or "Data ref: NCBI Sequence Read Archive PRJNA342805, 2017". In the Reference list, data citations must be labeled with "[DATASET]". A data reference must provide the database name, accession number/identifiers and a resolvable link to the landing page from which the data can be accessed at the end of the reference. Further instructions are available at .

9) We replaced Supplementary Information with Expanded View (EV) Figures and Tables that are collapsible/expandable online. A maximum of 5 EV Figures can be typeset. EV Figures should be cited as 'Figure EV1, Figure EV2" etc... in the text and their respective legends should be included in the main text after the legends of regular figures.
- For the figures that you do NOT wish to display as Expanded View figures, they should be bundled together with their legends in a single PDF file called *Appendix*, which should start with a short Table of Content. Appendix figures should be referred to in the main text as: "Appendix Figure S1, Appendix Figure S2" etc.
- Additional Tables/Datasets should be labeled and referred to as Table EV1, Dataset EV1, etc. Legends have to be provided in a separate tab in case of .xls files. Alternatively, the legend can be supplied as a separate text file (README) and zipped together with the Table/Dataset file.
See detailed instructions here:
.

10) The paper explained: EMBO Molecular Medicine articles are accompanied by a summary of the articles to emphasize the major findings in the paper and their medical implications for the non-specialist reader. Please provide a draft summary of your article highlighting
- the medical issue you are addressing,
- the results obtained and
- their clinical impact.
This may be edited to ensure that readers understand the significance and context of the research. Please refer to any of our published articles for an example.

11) For more information: There is space at the end of each article to list relevant web links for further consultation by our readers. Could you identify some relevant ones and provide such information as well? Some examples are patient associations, relevant databases, OMIM/proteins/genes links, author's websites, etc...

12) Author contributions: the contribution of every author must be detailed in a separate section (before the acknowledgments).

13) Conflict of interest: We updated our journal's competing interests policy in January 2022 and request authors to consider both actual and perceived competing interests. Please review the policy https://www.embopress.org/competing-interests and update your competing interests if necessary.

14) Every published paper now includes a 'Synopsis' to further enhance discoverability. Synopses are displayed on the journal webpage and are freely accessible to all readers. They include a short stand first (maximum of 300 characters, including space) as well as 2-5 one-sentences bullet points that summarizes the paper. Please write the bullet points to summarize the key NEW findings. They should be designed to be complementary to the abstract - i.e. not repeat the same text. We encourage inclusion of key acronyms and quantitative information (maximum of 30 words / bullet point). Please use the passive voice. Please attach these in a separate file or send them by email, we will incorporate them accordingly.
Please also suggest a striking image or visual abstract to illustrate your article as a PNG file 550 px wide x 300-600 px high.

15) As part of the EMBO Publications transparent editorial process initiative (see our Editorial at http://embomolmed.embopress.org/content/2/9/329), EMBO Molecular Medicine will publish online a Review Process File (RPF) to accompany accepted manuscripts.
In the event of acceptance, this file will be published in conjunction with your paper and will include the anonymous referee reports, your point-by-point response and all pertinent correspondence relating to the manuscript. Let us know whether you agree with the publication of the RPF and as here, if you want to remove or not any figures from it prior to publication.
Please note that the Authors checklist will be published at the end of the RPF.

EMBO Molecular Medicine has a "scooping protection" policy, whereby similar findings that are published by others during review or revision are not a criterion for rejection. Should you decide to submit a revised version, I do ask that you get in touch after three months if you have not completed it, to update us on the status.

I look forward to receiving your revised manuscript.

Yours sincerely,

Lise Roth

Lise Roth, PhD
Senior Editor
EMBO Molecular Medicine

***

Rev_Com_number: RC-2023-01982
New_manu_number: EMM-2023-18144
Corr_author: Gerhardt
Title: Single-cell profiling and zebrafish avatars reveal LGALS1 as immunomodulating target in glioblastoma

Reviewer #1

Evidence, reproducibility and clarity:

Summary: the paper suggested a new approach to study in vivo possible interaction between glioblastoma (GBM) cells and glioblastoma associated macrophages (GAMs). By using single cells transcriptome profiling and in vitro and in vivo functional experiments the authors also suggested LGALS1 as possible key factor in the suppression of the immune system and a new target for immune modulation in glioma patients. The experimental plan is well described, and the results are beautifully presented using images, clear drawings, and videos.

Major comments: none

Minor comments:

1) *The number of zebrafish embryos analyzed after the xenograft is highly variable (e.g. 3-18; 4-22 in Figure 6). These numbers can be reported in the results section (not only in the legends) and the authors may comment on them in the discussion. The reproducibility of the xenotransplant experiments is always challenging as it is quite difficult to inject the same number of cells in every embryo and to have the same survival rate of injected cells and of transplanted embryos. For these reasons the volume of each xenograft can vary significantly in different embryos and in different experimental sessions. Accordingly, the number of macrophages associated to the tumor can vary and the statistical analysis can be deeply influenced by the number of replicates for each experimental group (a group with 3 embryos is very different in terms of quality and quantity of information in respect to a group of 18 embryos). It could be useful for the reader, who has no experience in this technique, be aware of the advantages and disadvantages of the procedure including the possible influence of the temperature (34°C instead of 37°C) on the embryo survival and the replication rate of glioma cells or macrophages behavior. Comment on these aspects does not weaken the power and the relevance of the model but unveil the critical aspects that every scientist has to evaluate before planning these kinds of experiments.*

Response: We agree with Reviewer #1 that the zebrafish avatar model is challenging, and it is difficult to obtain reproducible tumor sizes and survival rates. To be even more transparent, we have added a few sentences about the variable *n* number in the Results section and a critical comment about it in the Discussion section.

2) *An aspect that could be interesting to address, to further validate the avatar model, is to monitor the level of pro-inflammatory cytokines (Tumor Necrosis Factor and Interleukin 1, 6, and 8) that are expressed at basal level in the early developing zebrafish embryos. Do their expression level increase after the xenotransplantation? Can the zebrafish cytokines affect the behavior of glioma associated macrophages (i.e. macrophages polarization)?*

Response: This is an interesting point, indeed. We have previously injected murine melanoma (B16) cells into *Tg(mpeg1:mCherry-F);Tg(TNFα:eGFP-F)* embryos, a TNFα reporter line. We did not see TNFα expression in the brain tissue (see Figure 1), neither in the uninjected control embryos, nor in the tumor-bearing embryos, suggesting that the expression level may be too low to affect macrophage polarization. Second, in the tumor-bearing embryos, some (but not all) macrophages expressed TNFα and their numbers seemed to decrease over time, as did the number of macrophages in general. This is consistent with previous reports (Póvoa *et al*, 2021), and with our hypothesis that the tumor induces a phenotype switch in GAMs. We further observed that TNFα-expressing macrophages mostly had a round, "tumor-attacking" phenotype, and some of them even phagocytized tumor cells, which can be appreciated from the cyan inside the macrophages. Notably, none of the macrophages in the control embryos expressed TNFα, indicating that the expression is induced by the tumor.

If we were to repeat this experiment with patient-derived GBM stem cell cultures (GSCCs), we would have to generate a new TNFα reporter line or label the GSCCs with a different fluorophore, since both use GFP for visualization. Thus, injecting GFP-labeled GSCCs into the TNFα reporter line available in the lab will not allow us to evaluate TNFα expression. Both strategies would be time consuming, and given that we did not detect TNFα expression in the brain tissue, zebrafish cytokines most likely do not affect GAMs.

We did not investigate other cytokines in the developing zebrafish, but we believe this is not essential for the following reasons: We are mainly interested in the differences between the patient-derived GSSCs, and since they are all used in the same avatar model, we expect that if zebrafish cytokines would have an effect on GAMs and their polarization, this effect would be present in all avatars, and can thus be ignored when comparing different GSCCs. More importantly, our findings in the zebrafish avatar model were consistent with those in the *in vitro* model. We observed the same phenotype switch in the co-culture model, indicating that the key interaction is between tumor cells and macrophages.

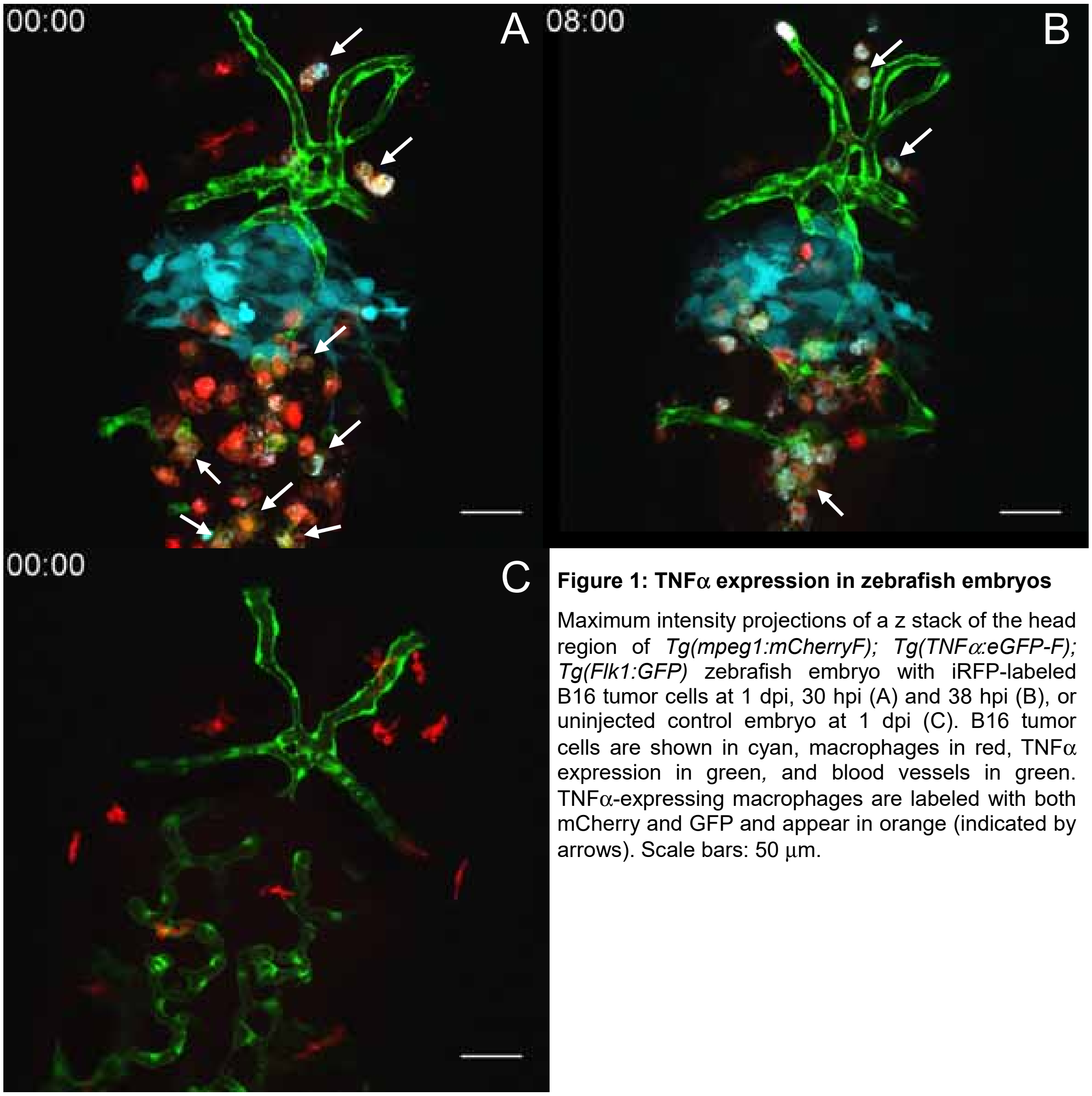

Figure 1: TNFα expression in zebrafish embryos

Maximum intensity projections of a z stack of the head region of *Tg(mpeg1:mCherryF); Tg(TNFα:eGFP-F); Tg(Flk1:GFP)* zebrafish embryo with iRFP-labeled B16 tumor cells at 1 dpi, 30 hpi (A) and 38 hpi (B), or uninjected control embryo at 1 dpi (C). B16 tumor cells are shown in cyan, macrophages in red, TNFα expression in green, and blood vessels in green. TNFα-expressing macrophages are labeled with both mCherry and GFP and appear in orange (indicated by arrows). Scale bars: 50 μm.

Significance:

Strengths and limitation. The manuscript is the result of a well-orchestrated effort to dissect a biological problem by complementary approaches and provide new data with high impact translational value. The image processing pipeline developed by the authors is a step forward in the in vivo analysis of cells interaction in living embryos. The identification of LGALS1 as a potential target for immune modulation can support the development of new therapeutical strategy implementing chemo- or immunotherapy protocols. The described zebrafish avatar can represent a new tool for personalized drug testing recapitulating in a in vivo model the heterogeneity of GBM found in patients.

Audience: All the scientist interested in cell biology, cancer cell biology, imaging techniques, translational medicine, in vivo models for cancer research, precision medicine.

Reviewer expertise: applied developmental biology

Reviewer #2

Evidence, reproducibility and clarity:

Finotto et al aim to address the polarisation of macrophages within GBM in their study. To do this, they have developed two different models. The first model is an in vitro co-culture model of patient derived GSC lines and human monocyte derived macrophages. This model was used for single cell sequencing to understand the transcriptomic changes of macrophages upon contact to GBM cells. The second model is a zebrafish xenograft model. Here GFP labeled GBM cells were transplanted into the larval zebrafish ventricle. These experiments were done in the transgenic mpeg zebrafish which allowed to monitor responses of macrophages in vivo.

In my opinion both models are not sophisticated enough to draw solid conclusions on macrophage polarisation in GBM. The in vitro model is highly artificial and is far from the complex situation in GBM. Within GBM the GAM population represents a heterogenous mix of resident microglia and infiltrating macrophages. These are influenced by the heterogeneous environment (which consists of tumour cells but also other host cells) and show diverse transcriptomic adaptations as shown in rodent models as well as sequencing studies of patient derived tumour samples. Studying monocyte derived macrophages in vitro does not provide any reliable insight.

Response: We understand the reviewer's concern about the complexity of our *in vitro* model. However, these simple models are needed to gain more insight into the complex *in vivo* situation. Others have demonstrated their usefulness in the past (C. Jayakrishnan *et al*, 2019; Zhou *et al*, 2022; Hubert *et al*, 2016; Chen *et al*, 2020; Coniglio *et al*, 2016; Li *et al*, 2022). Moreover, it may be advantageous to look at only two different cell types and unravel their reciprocal interaction, without the influence of other cell types, making it too complex to draw conclusions. We acknowledge that GAMs are a heterogeneous mix of both microglia and bone marrow-derived macrophages. Considering that bone marrow-derived macrophages have been shown to play an important role in tumor progression and are by far the most abundant immune cell population in GBM tumors (and their number even increases in recurrent GBM) (Pombo Antunes *et al*, 2021; Abdelfattah *et al*, 2022), we chose to focus initially on bone marrow-derived macrophages. Notably, it has already been reported that microglia were associated with significantly better survival, suggesting that they are anti-tumorigenic, whereas macrophages were associated with worse survival, suggesting that they are pro-tumorigenic ((Pombo Antunes *et al*, 2021; Abdelfattah *et al*, 2022). This justifies our approach to focus on this cell type. Furthermore, although this model may be rather simplistic, it allowed us to screen different GSCCs side by side in a standardized way, through which we found an apparent phenotype switch within the macrophages, even without the complex interplay with other cell types. Because the results obtained using the *in vitro* model were also confirmed in GBM patient material and KO experiments in the zebrafish avatar model, our work shows that reliable and important insights can be derived. This, combined with its simplicity, makes our co-culture model an exceptionally relevant model that is scalable, screenable and allows us to study the effect of perturbations. Finally, the immunosuppressive role of the target we identified using this model, *LGALS1*, has been previously demonstrated by others (Verschuere *et al*, 2014; Van Woensel *et al*, 2017; Chen *et al*, 2019), which proves our approach is valid.

Although the zebrafish can be a great model to understand the progression of tumours and the role of immune cells, I don't think that the model developed by the authors is suitable to address their questions. Transplantation of GBM cells into the the ventricle of larval zebrafish doesn't seem to be the right approach here. The poor survival of the transplanted cells is a clear indication of that. Many other groups have reported growth and proliferation of human cancer cells in the larval zebrafish. Direct transplantation into the brain parenchyma would be the better approach here. The brain parenchyma would provide the right environment for the GBM cells including a resident microglial population. This would also allow to study the complex mix of microglia and infiltrating macrophages in the context of GBM.

Response: The reviewer does not specify which articles have reported growth and proliferation of human cancer cells in zebrafish larvae. Most research groups describing this, either did not follow tumor growth/proliferation over time, or used immortalized cell lines (Vargas-Patron *et al*, 2019; Pan *et al*, 2020; Pudelko *et al*, 2018; Breznik *et al*, 2017; Vittori *et al*, 2017; Hamilton *et al*, 2016), which obviously have a much higher proliferation rate than the patient-derived cell lines used in this work. Second, although the number of patient-derived tumor cells decreases over time, we observed a clear invasive and migratory behavior, indicating that the human tumor cells reside well in the zebrafish brain microenvironment. Furthermore, it is important to note that the zebrafish avatars are grown at 34°C, a temperature that is suboptimal for tumor cell growth. The tumor cells still proliferate, albeit at a lower rate than at 37°C. To our knowledge, there is only one publication that reports the growth of patient-derived GBM tumors over time (Almstedt *et al*, 2022). However, prior to injection, patient-derived GBM cells were resuspended in medium containing polyvinylpyrrolidone, a polymer that enhances extracellular matrix deposition and cell proliferation. Furthermore, the authors observed substantial differences in proliferative capacity, ranging from growth to decline of signal, and displayed only two patient-derived cell lines with growing tumors. Similar to our findings, another article has demonstrated that injected patient-derived GBM tumor cells progressively underwent mitotic arrest, while maintaining an invasive and aggressive growth pattern (Rampazzo *et al*, 2013).

Although we have injected tumor cells into the hindbrain ventricle, they end up in the brain parenchyma, as evidenced by the presence of the typical brain vasculature of the zebrafish embryo. Notably, in *Tg(mpeg1:mCherryF)*^{ump2} zebrafish embryos, both macrophages and microglia are labeled with mCherry, meaning that we have studied both cell types in our zebrafish avatar model. Therefore, we consider the reviewer's comment to be unfounded.

Significance:

In my opinion both models are not sophisticated enough to draw solid conclusions on macrophage polarisation in GBM.

General response: We believe that there is an urgent need for good preclinical models that are scalable, flexible and screenable, which could ultimately be used to test new drugs or guide patient treatment in a personalized manner. In this work, we have focused on the differences between several GBM patients in scalable and screenable models, where perturbations (CRISPR KO, compounds, etc.) can be applied. We agree that our models are simplified representations of the clinical situation, as are all preclinical models, and like all models, they have advantages and disadvantages. We deliberately use simplified models to achieve the goals of scalability and screenability, where, under controlled conditions, differential behavior of GBM tumor cells derived from various patients can be systematically compared in how they affect a particular environment (*i.e.* the zebrafish brain and cultured macrophages). Obviously, such observations require further validation in clinical samples, which we have done. Furthermore, other research groups have shown that preclinical models are very useful to gain new insights in the search for new GBM treatments. We would like to emphasize that we are not making any strong claims regarding translation to the clinic. We are simply showing that these models are very useful for GBM research, while providing new opportunities to identify promising immunomodulating targets. The role of the target we identified, *LGALS1*, has been previously demonstrated by others, validating our approach. Finally, we also confirmed our findings in patient material, and our KO experiments show a clear and non-ignorable phenotype, proving that our models are indeed useful.

Reviewer #3

Evidence, reproducibility and clarity:

In this study, Finotto and colleagues developed patient-derived glioblastoma (GBM) stem cell cultures from 7 patients. These GBM stem cell cultures were either co-cultured in vitro with human macrophages combined with single-cell RNA sequencing or injected into the orthotopic zebrafish xenograft to study live GBM-macrophage/microglia interactions. Authors aimed at studying tumor heterogeneity and GBM-associated macrophages (GAMs) which often exhibit immunosuppressive features that promote tumor progression. Their analyses revealed substantial heterogeneity across GBM patients in GBM-induced macrophages polarization and the ability to attract and activate GAMs

- features that correlated with patient survival. Also authors show 3 distinct macrophage subclusters (MC1-3), highlighting that the simple M1/M2 polarization phenotypes is too reductive and there are no clear "markers". Authors associate these profiles with morphology and macrophage behaviour. Differential gene expression analysis, immunohistochemistry on original tumor samples, and knock-out experiments in zebrafish subsequently identified / confirmed that LGALS1 as a primary regulator of immunosuppression.

Cheng et (DOI: 10.1002/ijc.32102) had previously shown the immunosuppression effect of LGALS1 - but this work shows as a proof of concept that the authors approach is a valuable and interesting approach to find immune regulators.

Response: We fully agree with Reviewer #3. In fact, the immunosuppressive role of *LGALS1* has already been described by several research groups (Van Woensel *et al*, 2017; Verschuere *et al*, 2014), which indeed proves that our approach is valid. The reference cited by the reviewer was already included in the manuscript, along with other references.

Major comments:

In general claims are supported by date - very carefully presented and well characterized data with numbers, stats. It is an interesting descriptive study that illustrates the complexity and diversity of glioblastoma and the induced TME. I just have a few comments or clarifications that I would like to have elucidated:

1) *I did not understand why not single cell sequence the original tumor - without in vitro passaging and have the original patient population of MACs/microglia and monocytes sequenced? In other words why sequence the in vitro system - with its inherent caveats of in vitro culturing and not the original tumor? Can you please clarify. I believe that it would be important and interesting to have the original tumor sequenced to compare to the in vitro platform and understand how the in vitro selection impacts on the tumor biology and even if it changes the heterogeneity and differential composition of the tumor and macrophage profiles.*

Response: We agree with Reviewer #3 that our *in vitro* model does indeed have limitations inherent to patient-derived cell culture models. However, we chose this model to specifically focus on the reciprocal interaction between GBM tumor cells and macrophages in a way that also allows us to study how perturbations affect these interactions. This is not possible when using original tumors (*e.g.* we cannot make KO cells, as we did for *LGALS1*, and study the effects of genes of interest). Furthermore, the original tumor is only a snapshot of the real situation, whereas in the co-culture model we deliberately manipulated the polarization process (GM-CSF) and analyzed the cells at a well-defined timepoint. (See also the response to the comment of Reviewer #2.)

We do have scRNA-seq data from one original tumor sample (LBT123) and have therefore analyzed it. Unfortunately, scRNA-seq is not available for the other tumor samples. Also, for some of the patients, there is no original material left to use for sequencing. For LBT123, we compared the scRNA-seq data from the original tumor with the scRNA-seq data from the co-culture model and included our findings in the revised manuscript (Fig EV4C-G). When integrating the datasets and performing subclustering of the GBM tumor cells, we found that while some tumor heterogeneity was lost during *in vitro* culturing, tumor cells from the original LBT123 tumor clustered closest with the LBT123 GSCC (Fig EV4C), as also evidenced by the clustering tree (Fig EV4D). Interestingly, we found similar macrophage subtypes in the *CD68*$^+$ cells from the original tumor as in the co-culture assay, except for MC3, the TransMo population that was artificially generated by GM-CSF stimulation (Fig EV4E-G). Moreover, these cells were most similar to the macrophages co-cultured with the LBT123 GSCC (Fig EV4G).

2) *Mac signatures - out of curiosity - authors could not find TNFa and IFN signatures in any population?*

Response: Our analyses did not reveal TNF or IFN as cluster signature genes. It is well known that IFN and TNF expression is low in GAMs (Wang *et al*, 2022; Roesch *et al*, 2018; Poon *et al*, 2017). Nevertheless, we examined the expression of TNF- and IFN-related genes (see Figure 2). TNF expression was low in all subclusters, but slightly higher in MC3, the GM-CSF-stimulated TransMos. We did not find IFN expression in the macrophage subclusters, but we did find low expression of some IFN receptors. We found a gradient for IFNGR1 with the highest expression in MC3, followed by

MC1 and the lowest expression in MC2. IFNGR2 was expressed at slightly higher levels in MC1 and MC2 compared to MC3. IFNAR1 and IFNAR2 were expressed at comparable low levels in all subclusters. Finally, IFNLR1 expression was higher in MC3 compared to the other two macrophage subclusters. Considering the overall low expression of IFN receptors, we believe that the differences in expression are rather negligible. Furthermore, it has been previously shown that IFN exerts its anti-tumor effect primarily through the responsiveness of endothelial cells and not of myeloid cells, such as macrophages (Kammertoens *et al*, 2017). Since vascular cells were not present in the co-culture model, the low IFN receptor expression is not surprising.

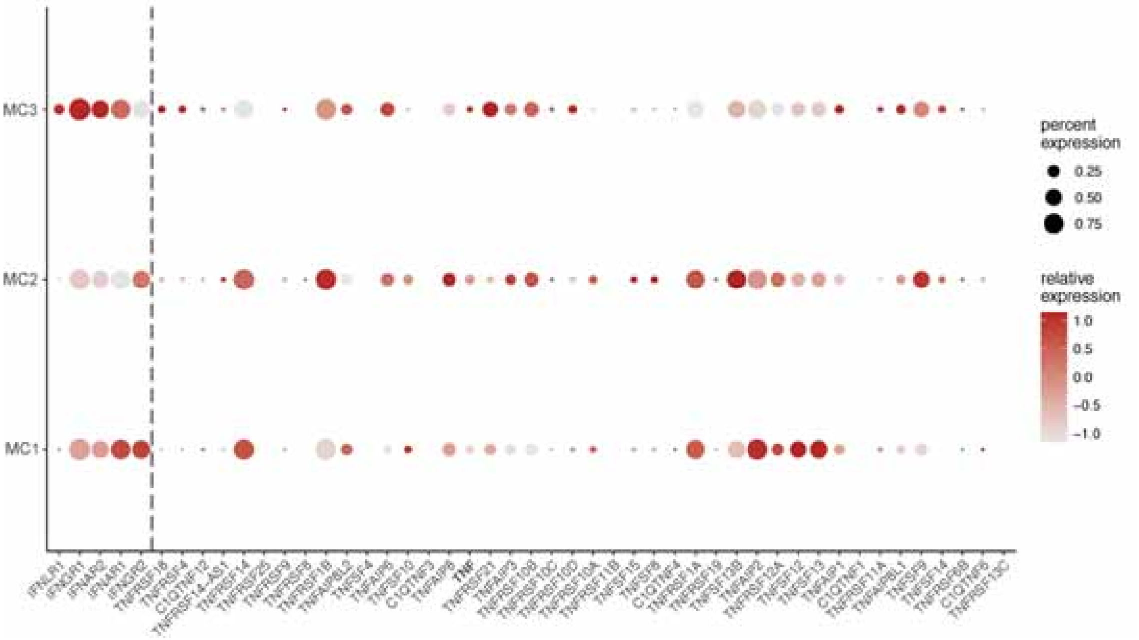

Figure 2: Dot plot showing expression of TNF- and IFN-related genes. Dot size indicates the percentage of cells in each macrophage subcluster expressing the gene, and dot color indicates the relative expression level.

3) Fig. 8 please show controls side by side with the KO

Response: We thank Reviewer #3 for this comment. We are not quite sure which panel the reviewer is referring to. If it is panel F, we agree with Reviewer #3 and have changed the order of the bars in the revised manuscript. If it is panel E, the corresponding control images are shown in Figure 5I. Since we believe that these images should not be repeated, we have added a figure reference to Figure 5I in the figure legend of Figure 8, in addition to the figure reference already provided in the text. Furthermore, images of all embryos are presented side by side in Figure EV5D-E.

4) *Figure 5: if each pair of images are separated and have the legend on top would be easier to read and follow.*

Response: We appreciate the comment that the figure should be intuitively easy to read and follow. However, we have chosen a compromise between overview and visibility of details (*e.g.* morphological features of GAMs). Since this figure already has the maximum width, the images would become smaller if they needed to be separated. Reducing the size would compromise the visibility of important details.

Significance:

It is a very interesting study, carefully designed and performed that highlights the heterogeneity of glioblastoma and how GBM can modulate the macrophage population into 3 different subsets. This study constitutes a proof of concept of the combination of and in vitro approach and an in vivo

approach to find new players and treatments in glioblastoma. I believe that it would be important and interesting to have a the original tumor sequenced to compare to the in vitro platform and understand how the in vitro selection impacts on the tumor biology and even if it changes the heterogeneity and differential composition of the tumor and macrophage profiles.

References:

Abdelfattah N, Kumar P, Wang C, Leu JS, Flynn WF, Gao R, Baskin DS, Pichumani K, Ijare OB, Wood SL, *et al* (2022) Single-cell analysis of human glioma and immune cells identifies S100A4 as an immunotherapy target. *Nat Commun* 13

Almstedt E, Rosen E, Gloger M, Stockgard R, Hekmati N, Koltowska K, Krona C & Nelander S (2022) Real-time evaluation of glioblastoma growth in patient-specific zebrafish xenografts. *Neuro Oncol* 24: 726–738

Breznik B, Motaln H, Vittori M, Rotter A & Turnšek TL (2017) Mesenchymal stem cells differentially affect the invasion of distinct glioblastoma cell lines. *Oncotarget* 8: 25482–25499

C. Jayakrishnan P, H. Venkat E, M. Ramachandran G, K. Kesavapisharady K, N. Nair S, Bharathan B, Radhakrishnan N & Gopala S (2019) In vitro neurosphere formation correlates with poor survival in glioma. *IUBMB Life* 71: 244–253

Chen JWE, Lumibao J, Leary S, Sarkaria JN, Steelman AJ, Gaskins HR & Harley BAC (2020) Crosstalk between microglia and patient-derived glioblastoma cells inhibit invasion in a three-dimensional gelatin hydrogel model. *J Neuroinflammation* 17

Chen Q, Han B, Meng X, Duan C, Yang C, Wu Z, Magafurov D, Zhao S, Safin S, Jiang C, *et al* (2019) Immunogenomic analysis reveals LGALS1 contributes to the immune heterogeneity and immunosuppression in glioma. *Int J Cancer* 145: 517–530

Coniglio S, Miller I, Symons M & Segall JE (2016) Coculture assays to study macrophage and microglia stimulation of glioblastoma invasion. *Journal of Visualized Experiments* 2016

Hamilton L, Astell KR, Velikova G & Sieger D (2016) A zebrafish live imaging model reveals differential responses of microglia toward glioblastoma cells in vivo. *Zebrafish* 13: 523–534

Hubert CG, Rivera M, Spangler LC, Wu Q, Mack SC, Prager BC, Couce M, McLendon RE, Sloan AE & Rich JN (2016) A three-dimensional organoid culture system derived from human glioblastomas recapitulates the hypoxic gradients and cancer stem cell heterogeneity of tumors found in vivo. *Cancer Res* 76: 2465–2477

Kammertoens T, Friese C, Arina A, Idel C, Briesemeister D, Rothe M, Ivanov A, Szymborska A, Patone G, Kunz S, *et al* (2017) Tumour ischaemia by interferon-γ resembles physiological blood vessel regression. *Nature* 545: 98–102

Li H, Yan X & Ou S (2022) Correlation of the prognostic value of FNDC4 in glioblastoma with macrophage polarization. *Cancer Cell Int* 22

Pan H, Xue W, Zhao W & Schachner M (2020) Expression and function of chondroitin 4-sulfate and chondroitin 6-sulfate in human glioma. *FASEB Journal* 34: 2853–2868

Pombo Antunes AR, Scheyltjens I, Lodi F, Messiaen J, Antoranz A, Duerinck J, Kancheva D, Martens L, De Vlaminck K, Van Hove H, *et al* (2021) Single-cell profiling of myeloid cells in glioblastoma across species and disease stage reveals macrophage competition and specialization. *Nat Neurosci* 24: 595–610

Poon CC, Sarkar S, Yong VW & Kelly JJP (2017) Glioblastoma-associated microglia and macrophages: targets for therapies to improve prognosis. *Brain* 140: 1548–1560

Póvoa V, Rebelo de Almeida C, Maia-Gil M, Sobral D, Domingues M, Martinez-Lopez M, de Almeida Fuzeta M, Silva C, Grosso AR & Fior R (2021) Innate immune evasion revealed in a colorectal zebrafish xenograft model. *Nat Commun* 12

Pudelko L, Edwards S, Balan M, Nyqvist D, Al-Saadi J, Dittmer J, Almlöf I, Helleday T & Bräutigam L (2018) An orthotopic glioblastoma animal model suitable for high-throughput screenings. *Neuro Oncol* 127: 415

Rampazzo E, Persano L, Pistollato F, Moro E, Frasson C, Porazzi P, Della Puppa A, Bresolin S, Battilana G, Indraccolo S, *et al* (2013) Wnt activation promotes neuronal differentiation of glioblastoma. *Cell Death Dis* 4

Roesch S, Rapp C, Dettling S & Herold-Mende C (2018) When immune cells turn bad - tumor-associated microglia/macrophages in glioma. *Int J Mol Sci* 19

Van Woensel M, Mathivet T, Wauthoz N, Rosière R, Garg AD, Agostinis P, Mathieu V, Kiss R, Lefranc F, Boon L, *et al* (2017) Sensitization of glioblastoma tumor micro-environment to chemo- and immunotherapy by Galectin-1 intranasal knock-down strategy. *Sci Rep* 7: 1–14

Vargas-Patron LA, Agudelo-Dueñãs N, Madrid-Wolff J, Venegas JA, González JM, Forero-Shelton M & Akle V (2019) Xenotransplantation of human glioblastoma in zebrafish larvae: in vivo imaging and proliferation assessment. *Biol Open* 8

Verschuere T, Toelen J, Maes W, Poirier F, Boon L, Tousseyn T, Mathivet T, Gerhardt H, Mathieu V, Kiss R, *et al* (2014) Glioma-derived galectin-1 regulates innate and adaptive antitumor immunity. *Int J Cancer* 134: 873–884

Vittori M, Breznik B, Hrovat K, Kenig S & Lah TT (2017) RECQ1 helicase silencing decreases the tumour growth rate of U87 glioblastoma cell xenografts in zebrafish embryos. *Genes (Basel)* 8

Wang G, Zhong K, Wang Z, Zhang Z, Tang X, Tong A & Zhou L (2022) Tumor-associated microglia and macrophages in glioblastoma: from basic insights to therapeutic opportunities. *Front Immunol* 13

Zhou F, Shi Q, Fan X, Yu R, Wu Z, Wang B, Tian W, Yu T, Pan M, You Y, *et al* (2022) Diverse macrophages constituted the glioma microenvironment and influenced by PTEN status. *Front Immunol* 13

15th Aug 2023

Dear Dr. Gerhardt,

Thank you for the submission of your revised manuscript to EMBO Molecular Medicine. We have now received the report from the referee who re-reviewed your manuscript (and had originally reviewed your manuscript for Review Commons as referee #3). As you will see below, this referee is supportive of publication pending minor revisions, and I am therefore pleased to inform you that we will be able to accept your manuscript once the following editorial points will be addressed:

1/ Referees comments and point-by-point rebuttal letter:
- Please address the remaining minor comment from the referee.

2/ Manuscript text:
- Please accept the previous changes, and only keep in track changes mode any new modification. Please check the related Data edited MS file from our data editors for potential queries or comments.
- Data Availability: as discussed previously, please note that we request an EGA accession number before online publication of your manuscript.
- Author contributions: CRediT has replaced the traditional author contributions section because it offers a systematic machine-readable author contributions format that allows for more effective research assessment. Please remove the Authors Contributions from the manuscript and use the free text boxes beneath each contributing author's name in our system to add specific details on the author's contribution. More information is available in our guide to authors.
- Acknowledgements: the complete funding information should be provided both in the acknowledgements section and in the submission system (currently, FWO PhD fellowship fundamental research (11L0822N and KOTK grant (KOTK/2018/11509/2) are not entered in the submission system. Please add the grant numbers)
- Disclosure statement and competing interests: We updated our journal's competing interests policy in January 2022 and request authors to consider both actual and perceived competing interests. Please review the policy https://www.embopress.org/competing-interests and update your competing interests if necessary. Please rename the section accordingly.

3/ Figures:
- EV tables: please remove the legends from the manuscript and add them to the files.
- Movies: please remove the legends from the manuscript and zip each with the corresponding movie file.
- Please make sure the figures are referenced in the right order in the manuscript (currently, Fig. 6A is called out before Fig. 5C). The movie callouts need to be corrected to Movie EV1, etc.

4/ Please note that all corresponding authors are required to supply an ORCID ID for their name upon submission of a revised manuscript. An ORCID identifier is currently missing for Frederik De Smet.

5/ I slightly edited your synopsis to fit our format and style, please let me know if you agree with the following or amend as you see fit:

scRNA-seq and live-imaging in zebrafish avatars were used to study the complex and dynamic interplay between GBM tumor cells and GAMs, and provide fresh perspectives and opportunities for discovering promising immune-modulating targets in GBM.

• GBM tumor cells display heterogeneity in GAM recruitment and activation.
• GAMs shift towards an immunosuppressive state, with the degree of GBM-induced GAM polarization varying significantly across GBM patients.
• GAM activation and polarization correlate with clinical outcome in GBM patients.
• LGALS1 plays a crucial role in regulating immunosuppression and affects tumor growth.
• Tumors that no longer express LGALS1 exhibit a less invasive phenotype.

Thanks for providing a synopsis image. While it is very nice, we rather request a scheme that summarizes the findings in the paper. Could you please provide such an image? (please note that it should be 550 px wide x 200-300 px high).

6/ As part of the EMBO Publications transparent editorial process initiative (see our Editorial at http://embomolmed.embopress.org/content/2/9/329), EMBO Molecular Medicine will publish online a Review Process File (RPF) to accompany accepted manuscripts.
This file will be published in conjunction with your paper and will include the anonymous referee reports, your point-by-point response and all pertinent correspondence relating to the manuscript. Let us know whether you agree with the publication of the RPF and as here, if you want to remove or not any figures from it prior to publication.
Please note that the Authors checklist will be published at the end of the RPF.

I look forward to receiving your revised manuscript.

With kind regards,

Lise

Lise Roth, PhD
Senior Editor
EMBO Molecular Medicine

***** Reviewer's comments *****

Referee #1 (Comments on Novelty/Model System for Author):

The are no perfect models - and all have their limitations and authors used 2 complementary models to get to their interesting findings.

Referee #1 (Remarks for Author):

I still believe the figures could improve to be more easy to follow with captions - on top and not hidden in the left corner- is really not easy to read the results- that is my only comment.
Otherwise authors answered to my concerns.

***
Rev_Com_number: RC-2023-01982
New_manu_number: EMM-2023-18144-V2
Corr_author: Gerhardt
Title: Single-cell profiling and zebrafish avatars reveal LGALS1 as immunomodulating target in glioblastoma

The authors addressed the remaining editorial issues.

4th Sep 2023

Dear Holger,

Thank you for submitting your revised files. I am pleased to inform you that your manuscript is accepted for publication and is now being sent to our publisher to be included in the next available issue of EMBO Molecular Medicine!

Congratulations on your interesting work,

With kind regards,

Lise

Lise Roth, Ph.D
Senior Editor
EMBO Molecular Medicine

Follow us on Twitter @EmboMolMed
Sign up for eTOCs at embopress.org/alertsfeeds

*** *** *** IMPORTANT INFORMATION *** *** ***

SPEED OF PUBLICATION
The journal aims for rapid publication of papers, using using the advance online publication "Early View" to expedite the process: A properly copy-edited and formatted version will be published as "Early View" after the proofs have been corrected. Please help the Editors and publisher avoid delays by providing e-mail address(es), telephone and fax numbers at which author(s) can be contacted.

Should you be planning a Press Release on your article, please get in contact with embomolmed@wiley.com as early as possible, in order to coordinate publication and release dates.

LICENSE AND PAYMENT:

All articles published in EMBO Molecular Medicine are fully open access: immediately and freely available to read, download and share.

EMBO Molecular Medicine charges an article processing charge (APC) to cover the publication costs. You, as the corresponding author for this manuscript, should have already received a quote with the article processing fee separately. Please let us know in case this quote has not been received.

Once your article is at Wiley for editorial production you will receive an email from Wiley's Author Services system, which will ask you to log in and will present you with the publication license form for completion. Within the same system the publication fee can be paid by credit card, an invoice, pro forma invoice or purchase order can be requested.

Payment of the publication charge and the signed Open Access Agreement form must be received before the article can be published online.

PROOFS

You will receive the proofs by e-mail approximately 2 weeks after all relevant files have been sent o our Production Office. Please return them within 48 hours and if there should be any problems, please contact the production office at embopressproduction@wiley.com.

Please inform us if there is likely to be any difficulty in reaching you at the above address at that time. Failure to meet our

deadlines may result in a delay of publication.

All further communications concerning your paper proofs should quote reference number EMM-2023-18144-V3 and be directed to the production office at embopressproduction@wiley.com.

EMBO Press Author Checklist

Corresponding Author Name: Holger Gerhardt & Frederik De Smet
Journal Submitted to: EMBO Molecular Medicine
Manuscript Number: EMM-2023-18144

USEFUL LINKS FOR COMPLETING THIS FORM
The EMBO Journal - Author Guidelines
EMBO Reports - Author Guidelines
Molecular Systems Biology - Author Guidelines
EMBO Molecular Medicine - Author Guidelines

Reporting Checklist for Life Science Articles (updated January
This checklist is adapted from Materials Design Analysis Reporting (MDAR) Checklist for Authors. MDAR establishes a minimum set of requirements in transparent reporting in the life sciences (see Statement of Task: 10.31222/osf.io/9sm4x). Please follow the journal's guidelines in preparing your
Please note that a copy of this checklist will be published alongside your article.

Abridged guidelines for figures
1. Data
The data shown in figures should satisfy the following conditions:
- the data were obtained and processed according to the field's best practice and are presented to reflect the results of the experiments in an accurate and unbiased manner.
- ideally, figure panels should include only measurements that are directly comparable to each other and obtained with the same assay.
- plots include clearly labeled error bars for independent experiments and sample sizes. Unless justified, error bars should not be shown for technical
- if n<5, the individual data points from each experiment should be plotted. Any statistical test employed should be justified.
- Source Data should be included to report the data underlying figures according to the guidelines set out in the authorship guidelines on Data

2. Captions
Each figure caption should contain the following information, for each panel where they are relevant:
- a specification of the experimental system investigated (eg cell line, species name).
- the assay(s) and method(s) used to carry out the reported observations and measurements.
- an explicit mention of the biological and chemical entity(ies) that are being measured.
- an explicit mention of the biological and chemical entity(ies) that are altered/varied/perturbed in a controlled manner.
- the exact sample size (n) for each experimental group/condition, given as a number, not a range;
- a description of the sample collection allowing the reader to understand whether the samples represent technical or biological replicates (including how many animals, litters, cultures, etc.).
- a statement of how many times the experiment shown was independently replicated in the laboratory.
- definitions of statistical methods and measures:
  - common tests, such as t-test (please specify whether paired vs. unpaired), simple χ2 tests, Wilcoxon and Mann-Whitney tests, can be unambiguously identified by name only, but more complex techniques should be described in the methods section;
  - are tests one-sided or two-sided?
  - are there adjustments for multiple comparisons?
  - exact statistical test results, e.g., P values = x but not P values < x;
  - definition of 'center values' as median or average;
  - definition of error bars as s.d. or s.e.m.

Please complete ALL of the questions below.
Select "Not Applicable" only when the requested information is not relevant for your study.

Materials

Newly Created Materials	Information included in the manuscript?	In which section is the information available? (Reagents and Tools Table, Materials and Methods, Figures, Data Availability Section)
New materials and reagents need to be available; do any restrictions apply?	Yes	Material and Methods + Data Availability section + Table EV2

Antibodies	Information included in the manuscript?	In which section is the information available? (Reagents and Tools Table, Materials and Methods, Figures, Data Availability Section)
For **antibodies** provide the following information:<br>- Commercial antibodies: RRID (if possible) or supplier name, catalogue number and or/clone number<br>- Non-commercial: RRID or citation	Yes	Table EV2

DNA and RNA sequences	Information included in the manuscript?	In which section is the information available? (Reagents and Tools Table, Materials and Methods, Figures, Data Availability Section)
**Short novel DNA or RNA including primers, probes**: provide the sequences.	Yes	Table EV2

Cell materials	Information included in the manuscript?	In which section is the information available? (Reagents and Tools Table, Materials and Methods, Figures, Data Availability Section)
**Cell lines:** Provide species information, strain. Provide accession number in repository **OR** supplier name, catalog number, clone number, and/**OR** RRID.	Not Applicable	
**Primary cultures:** Provide species, strain, sex of origin, genetic modification status.	Yes	Material and Methods + Fig 1B
Report if the cell lines were recently **authenticated** (e.g., by STR profiling) and tested for mycoplasma contamination.	Yes	Material and Methods

Experimental animals	Information included in the manuscript?	In which section is the information available? (Reagents and Tools Table, Materials and Methods, Figures, Data Availability Section)
**Laboratory animals or Model organisms:** Provide species, strain, sex, age, genetic modification status. Provide accession number in repository **OR** supplier name, catalog number, clone number, **OR** RRID.	Yes	Material and Methods + Data Availability section + Table EV2
**Animal observed in or captured from the field:** Provide species, sex, and age where possible.	Not Applicable	
Please detail **housing and husbandry conditions**.	Yes	Material and Methods

Plants and microbes	Information included in the manuscript?	In which section is the information available? (Reagents and Tools Table, Materials and Methods, Figures, Data Availability Section)
**Plants:** provide species and strain, ecotype and cultivar where relevant, unique accession number if available, and source (including location for collected wild specimens).	Not Applicable	
**Microbes:** provide species and strain, unique accession number if available, and source.	Not Applicable	

Human research participants	Information included in the manuscript?	In which section is the information available? (Reagents and Tools Table, Materials and Methods, Figures, Data Availability Section)
If collected and within the bounds of privacy constraints report on age, sex and gender or ethnicity for all study participants.	Not Applicable	

Core facilities	Information included in the manuscript?	In which section is the information available? (Reagents and Tools Table, Materials and Methods, Figures, Data Availability Section)
If your work benefited from core facilities, was their service mentioned in the acknowledgments section?	Yes	Acknowledgements section (Leuven Viral Vector Core)

Design

Study protocol	Information included in the manuscript?	In which section is the information available? (Reagents and Tools Table, Materials and Methods, Figures, Data Availability Section)
If study protocol has been **pre-registered, provide DOI in the manuscript**. For clinical trials, provide the trial registration number **OR** cite DOI.	Not Applicable	
Report the **clinical trial registration number** (at ClinicalTrials.gov or equivalent), where applicable.	Not Applicable	

Laboratory protocol	Information included in the manuscript?	In which section is the information available? (Reagents and Tools Table, Materials and Methods, Figures, Data Availability Section)
Provide DOI OR other citation details if **external detailed step-by-step protocols** are available.	Not Applicable	

Experimental study design and statistics	Information included in the manuscript?	In which section is the information available? (Reagents and Tools Table, Materials and Methods, Figures, Data Availability Section)
Include a statement about **sample size** estimate even if no statistical methods were used.	Yes	Materials and Methods
Were any steps taken to minimize the effects of subjective bias when allocating animals/samples to treatment (e.g. **randomization procedure**)? If yes, have they been described?	Yes	Material and Methods
Include a statement about **blinding** even if no blinding was done.	Yes	Material and Methods
Describe **inclusion/exclusion criteria** if samples or animals were excluded from the analysis. Were the criteria pre-established?<br><br>If sample or data points were omitted from analysis, report if this was due to attrition or intentional exclusion and provide justification.	Yes	Material and Methods
For every figure, are **statistical tests** justified as appropriate? Do the data meet the assumptions of the tests (e.g., normal distribution)? Describe any methods used to assess it. Is there an estimate of variation within each group of data? Is the variance similar between the groups that are being statistically compared?	Yes	Figure legends + Material and Methods

Sample definition and in-laboratory replication	Information included in the manuscript?	In which section is the information available? (Reagents and Tools Table, Materials and Methods, Figures, Data Availability Section)
In the figure legends: state number of times the experiment was **replicated** in laboratory.	Yes	Figure legends
In the figure legends: define whether data describe **technical or biological replicates**.	Yes	Figure legends

Ethics

Ethics	Information included in the manuscript?	In which section is the information available? (Reagents and Tools Table, Materials and Methods, Figures, Data Availability Section)
Studies involving **human participants**: State details of **authority granting ethics approval** (IRB or equivalent committee(s), provide reference number for approval.	Yes	Materials and Methods
Studies involving **human participants**: Include a statement confirming that **informed consent** was obtained from all subjects and that the experiments conformed to the principles set out in the WMA Declaration of Helsinki and the Department of Health and Human Services Belmont Report.	Yes	Materials and Methods
Studies involving **human participants: For publication of patient photos**, include a statement confirming that consent to publish was obtained.	Not Applicable	
Studies involving experimental **animals**: State details of **authority granting ethics approval** (IRB or equivalent committee(s), provide reference number for approval. Include a statement of compliance with ethical regulations.	Yes	Materials and Methods
Studies involving **specimen and field samples:** State if relevant **permits** obtained, provide details of authority approving study; if none were required, explain why.	Not Applicable	

Dual Use Research of Concern (DURC)	Information included in the manuscript?	In which section is the information available? (Reagents and Tools Table, Materials and Methods, Figures, Data Availability Section)
Could your study fall under dual use research restrictions? Please check biosecurity documents and list of **select agents and toxins** (CDC): https://www.selectagents.gov/sat/list.htm	Not Applicable	
If you used a select agent, is the security level of the lab appropriate and reported in the manuscript?	Not Applicable	
If a study is subject to dual use research of concern regulations, is the name of the **authority granting approval and reference number** for the regulatory approval provided in the manuscript?	Not Applicable	

Reporting

The MDAR framework recommends adoption of discipline-specific guidelines, established and endorsed through community initiatives. Journals have their own policy about requiring specific guidelines and recommendations to complement MDAR.

Adherence to community standards	Information included in the manuscript?	In which section is the information available? (Reagents and Tools Table, Materials and Methods, Figures, Data Availability Section)
State if relevant guidelines or checklists (e.g., **ICMJE, MIBBI, ARRIVE, PRISMA**) have been followed or provided.	Yes	Materials and Methods
For **tumor marker prognostic studies,** we recommend that you follow the **REMARK** reporting guidelines (see link list at top right). See author guidelines, under 'Reporting Guidelines'. Please confirm you have followed these guidelines.	Not Applicable	
For **phase II and III randomized controlled trials**, please refer to the **CONSORT** flow diagram (see link list at top right) and submit the CONSORT checklist (see link list at top right) with your submission. See author guidelines, under 'Reporting Guidelines'. Please confirm you have submitted this list.	Not Applicable	

Data Availability

Data availability	Information included in the manuscript?	In which section is the information available? (Reagents and Tools Table, Materials and Methods, Figures, Data Availability Section)
Have **primary datasets** been deposited according to the journal's guidelines (see 'Data Deposition' section) and the respective accession numbers provided in the Data Availability Section?	Yes	Data Availability section
Were **human clinical and genomic datasets** deposited in a public access-controlled repository in accordance to ethical obligations to the patients and to the applicable consent agreement?	Yes	Data Availability section
Are **computational models** that are central and integral to a study available without restrictions in a machine-readable form? Were the relevant accession numbers or links provided?	Yes	Data Availability section
If publicly available data were reused, provide the respective **data citations in the reference list**.	Yes	References + Data Availability section
